# Treewidth-based algorithms for the small parsimony problem on networks

**DOI:** 10.1186/s13015-022-00216-w

**Published:** 2022-08-20

**Authors:** Celine Scornavacca, Mathias Weller

**Affiliations:** 1 ISEM, Université de Montpellier, CNRS, IRD, EPHE, Montpellier, France; 2 LIGM, Université Gustave Eiffel, CNRS, Paris, France

**Keywords:** Phylogenetics, Parsimony, Phylogenetic networks, Parameterized complexity, Dynamic programming, Treewidth

## Abstract

**Background:**

Phylogenetic reconstruction is one of the paramount challenges of contemporary bioinformatics. A subtask of existing tree reconstruction algorithms is modeled by the Small Parsimony problem: given a tree *T* and an assignment of character-states to its leaves, assign states to the internal nodes of *T* such as to minimize the *parsimony score*, that is, the number of edges of *T* connecting nodes with different states. While this problem is polynomial-time solvable on trees, the matter is more complicated if *T* contains reticulate events such as hybridizations or recombinations, i.e. when *T* is a network. Indeed, three different versions of the parsimony score on networks have been proposed and each of them is NP-hard to decide. Existing parameterized algorithms focus on combining the number *c* of possible character-states with the number of reticulate events (per biconnected component).

**Results:**

We consider the parameter treewidth *t* of the underlying undirected graph of the input network, presenting dynamic programming algorithms for (slight generalizations of) all three versions of the parsimony problem on size-*n* networks running in times $c^t {n^{O(1)}}$, $(3c)^t {n^{O(1)}}$, and $6^{tc}n^{O(1)}$, respectively. Our algorithms use a formulation of the treewidth that may facilitate formalizing treewidth-based dynamic programming algorithms on phylogenetic networks for other problems.

**Conclusions:**

Our algorithms allow the computation of the three popular parsimony scores, modeling the evolutionary development of a (multistate) character on a given phylogenetic network of low treewidth. Our results subsume and improve previously known algorithm for all three variants. While our results rely on being given a “good” tree-decomposition of the input, encouraging theoretical results as well as practical implementations producing them are publicly available. We present a reformulation of tree decompositions in terms of “agreeing trees” on the same set of nodes. As this formulation may come more natural to researchers and engineers developing algorithms for phylogenetic networks, we hope to render exploiting the input network’s treewidth as parameter more accessible to this audience.

## Introduction

Molecular phylogenetic reconstruction consists in inferring a well-founded evolutionary scenario of a set of species from molecular data [1]. An evolutionary scenario, also called a *phylogeny*, is usually represented by a directed tree with a unique source called *root*. In a phylogeny, the tips of the tree are associated to extant species for which we have data, and each internal node represents an extinct species giving rise to new species—a *speciation*. Therefore, each internal node represents the hypothetical ancestor of all species below it, and the root models the lowest common ancestor of all the species at the tips.

### Parsimony on trees

In this paper, molecular data consists of a set of molecular sequences (e.g. DNA or protein sequences) of the same length (one sequence per species). This kind of data can be seen as a matrix *M* of *n* sequences, each having *m* characters (exhibiting one of *c* possible states) where $M_{i,j}$ corresponds to the state of the *j*th character exhibited by the *i*th species. There are several methods to reconstruct well-founded phylogenies from matrices of characters [1]. They are all based on the idea of retrieving similarities among species by comparing the states taken by these species at the different characters of *M*. Here, we will focus on *parsimony methods*. The main hypothesis of these methods is that character changes are not frequent. Thus, the phylogenies that best explain the data are those requiring the fewest evolutionary changes, i.e. the ones having the optimal *parsimony score*, formally defined in “Parsimony”. The problem of finding the optimal parsimony score for a given phylogeny *T* with respect to an $n\times m$ matrix on a finite set of *c* character states is called the Small Parsimony problem and can be solved in $O(n\cdot m\cdot c)$ time [2] since each column in the matrix can be analyzed independently in linear time. When *T* is unknown, the problem of finding the phylogeny minimizing the parsimony score is called the Big Parsimony problem. This latter is known to be NP-hard and numerous heuristic techniques for it are known [1].

### Parsimony on networks

**Fig. 1:**
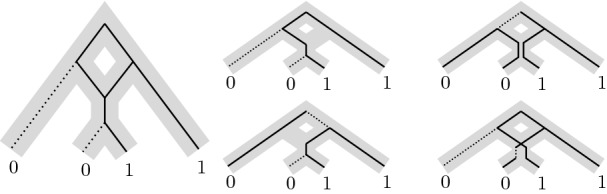
Example for parsimony scores of a network (in gray). Black edges participate in the score (solid = score 0, dotted = score 1). For the hardwired score (left), all edges of the network are considered. For the softwired score (2 possible trees: middle), only edges of any switching are considered. For the parental score (4 possible trees: middle & right), a tree is inscribed in the network

When the evolution of the species of interest include, in addition to speciations, reticulate events such as *hybridizations* or *recombinations*, a single species may inherit from multiple direct ancestors. In this case, the phylogenies are no longer represented by rooted trees but by rooted DAGs [3] called *networks*. When scoring a given network, three very different definitions of the parsimony score have been proposed: the *hardwired* [4], the *softwired* [5, 6], and the *parental* parsimony score [7]. Roughly, the hardwired score takes into account all edges of the given network (characters are inherited from all parents), the softwired score takes only the edges of any “switching” (each character is inherited from one parent), and the parental score allows embedding lineages into the network (each allele of a character is inherited from one parent). See “Parsimony” for details and Fig. 1 for an example. While these definitions coincide for trees, they give rise to three different small parsimony problems for networks.

When tracing mutually dependent characters (e.g. different genomic locations in a same non-recombinant region) on networks, we also have to make sure that dependent characters are inherited from the same parent (some columns of the matrix have to use the same “switching”/“embedding”). To avoid dealing with this problem, the small parsimony problems on networks have been studied predominantly under the assumption of independent genomic locations. This boils down to having $m=1$ since each column of the matrix can be analyzed independently (as is the case for the small parsimony problem on trees). Another popular restriction is to consider *binary* networks, in which the root has outdegree 2, tips have indegree 1, and internal nodes have either indegree 1 and outdegree 2 (speciations) or indegree 2 and outdegree 1 (reticulations).

The hardwired small parsimony problem has been proven NP-hard and APX-hard whenever the number of states that a character can take, denoted *c*, is strictly greater than 2, and polynomial-time solvable for binary characters [8]. A polynomial-time 1.35-approximation for all *c* and a $\frac {12}{11}$-approximation for $c=3$ have been proposed [8]. Additionally, the problem has been shown fixed-parameter tractable (FPT) in the parsimony score [8, $2^p \cdot O(\mathrm {min}(q^{\frac {2}{3}},\sqrt{z})\cdot q)$ time], and in $c+r$ [9, $O(n\cdot c^{r+2})$ time], where *n*, *q*, *z* are the number of leaves, vertices and edges in the phylogenetic network and *p* and *r* are the hardwired parsimony score and the number of reticulate events in the network.

The softwired small parsimony problem is also NP-hard and APX-hard [8, 10] for binary characters, and not FPT in the parsimony score (it is NP-hard to decide if the softwired parsimony score is 1). Also, it has been shown that, for any constant $\epsilon >0$, no $n^{1-\epsilon }$ approximation can be computed in polynomial time, unless $\text {P} = \text {NP}$. On the positive side, the problem is FPT in $c+r$ [6, 8, $O(2^r \cdot n \cdot c)$ time] and $c+\ell$ [8, 11, $O(2^\ell \cdot c^2 \cdot q \cdot z)$ time], where $\ell$ is the maximum number of reticulations over all biconnected components of the network (also called the *level* of the network).

Unsurprisingly, the parental small parsimony problem has also been proven NP-hard, even for very restricted classes of networks, but it is FPT both with respect to $c+r$ and with respect to $c+\ell$ [12, $O((2^c)^{r+2} \cdot q)$ and $O((2^c)^{\ell +3} \cdot q)$ time].

In this paper, we consider the case of independent characters, showing that the three variants of the small parsimony problem on networks are fixed-parameter tractable with respect to $c+t$ (running in time $O(T+c^{t+1}\cdot z)$, $O(T + c^t\cdot (3^t\cdot c \cdot q+z))$, and $O(T+ 6^{t\cdot c} \cdot 4^{t\cdot \log (c)}\cdot z)$), provided that a width-*t* tree-decomposition of the input network *N* can be computed in *T* time (this is the case for *t* equaling the treewidth of *N* and $T\in 2^{O(k^2)}$ [13]). Our proofs are constructive in the sense that a dynamic programming algorithm is provided for each version of the problem. The main strength of our algorithms lies in their parameterization, since the treewidth can be arbitrarily small, even for growing values of $\ell$. An implication of parameterizing by the treewidth is that our algorithms run in polynomial time even on classes of networks on which previously known algorithms require exponential time^1^ while our algorithms run in polynomial time on all classes of networks that were previously known to allow for polynomial-time algorithms. Hence, our algorithms can potentially be orders of magnitude faster than the state-of-the-art solutions. Moreover, our formulations are not limited to binary networks and they can take into account polymorphism as well as external information controlling the states that ancestral species may take.

### Treewidth for phylogenetic networks

The treewidth of a graph can roughly be described as a measure of “tree-likeness” and it ranks among the smallest of such parameters [14] (in particular, the treewidth can be seen to be smaller than the level $\ell$ on any network). Together with the fact that it facilitates the design of dynamic programming algorithms, this explains the enormous popularity the treewidth received in the parameterized complexity community [15, 16]. Starting with the groundbreaking work of Bryant and Lagergren [17] (using the celebrated result of Courcelle [18]), treewidth also gained traction with researchers studying algorithms for phylogenetics-related problems (surveyed in [19]). While this yielded some algorithms parameterized by the treewidth *of the display graph* of multiple trees (the result of “gluing” all trees at their leaves), we are not aware of any algorithms parameterized by the treewidth of the input network. In an attempt to facilitate the use of this parameter in future work, we dedicate Sect. “An alternative formulation of treewidth” to presenting a “phylogenetics-friendly” formulation by representing tree-decompositions of the input network as a rooted tree $\Gamma$ on the same vertex set as the network. In particular, this formulation generalizes our previously considered parameter “scanwidth” [20], which can be seen as a variant of treewidth that takes directness into account. While we expected scanwidth-based dynamic programming formulations to be easier and more straight-forward than their treewidth-counterparts, this comes at the cost of the scanwidth being potentially arbitrarily larger than the treewidth. Intuitively speaking, we expect scanwidth dynamic programming to be easier since phylogenetic networks exhibit a “natural flow of information”: most often, we know everything about the leaves, but the more we approach the root, the more information has to be inferred from the lower parts. In contrast to the scanwidth-layout, tree-decompositions disregard edge directions and, thereby, this “natural flow”. Thus, while using the scanwidth allows for more naïve and intuitive dynamic programming formulations, using the treewidth requires more care and ingenuity.

**Fig. 2:**
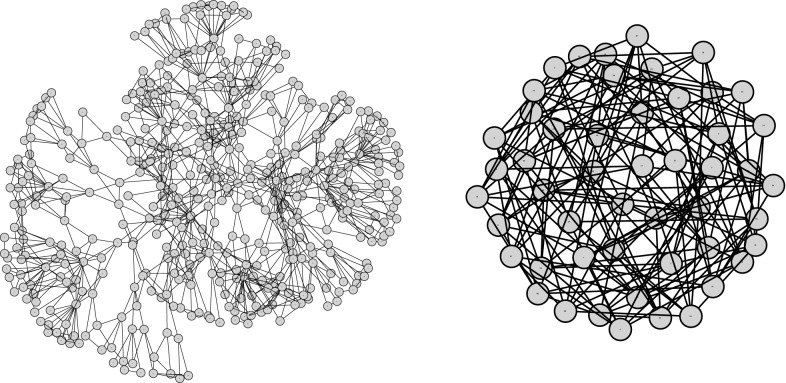
Tamaki’s tree-decomposer [23] has a harder time with the right, more tangled instance (50 nodes, 175 edges, treewidth 25 computed in 79s) than with the larger instance on the left (465 nodes, 1004 edges, treewidth 9 computed in 0.5s), illustrating that tangledness is a more important factor than size. Indeed, both instances display a tangledness that already exceeds what we expect to see in real-world phylogenetic networks. The instances are ex065 (right) and ex011 (left) of the PACE2017 challenge [22]

Since we will suppose that a (not necessarily optimal) tree-decomposition of the input network is given in the input, let us discuss the current state-of-the-art for computing good decompositions. Optimal decompositions are indeed very hard to compute, with even the best-known parameterized algorithm being considered impractical (see survey [15]). This gloomy cloud has, however, two silver linings: First, if we do not insist on optimality, then we can use a recently published algorithm to compute 2-approximated tree-decompositions in $2^{O(k)}n^{O(1)}$ time [21]. We will state our results in a way that allows plugging-in any algorithm that computes or approximates tree decompositions. Second, with development driven by recent instances of the PACE challenge [22], more practical exact algorithms to compute tree decompositions are now available as well [23]. Herein, the running times of Tamaki’s implementation [23] are hard to predict and show erratic behavior even for fixed graph size. As expected, however, examples for high running times occur only for instances with high treewidth, that is, for “highly tangled” networks (see Fig. 2 for two select examples). This hints towards some hidden properties of the input networks that govern the complexity of treewidth computations As we expect “natural networks” to be only moderately tangled, we think that existing algorithms, exact and approximative, are currently well-enough developed to deal with real world phylogenetic networks in reasonable timeframes. Indeed, we would welcome efforts similar to those made for the treewidth to also be made for the previously discussed scanwidth, which is also hard to compute [20].

For ease of presentation, the three main proofs (correctness of the dynamic programming formulations) are given as high-level sketches and their more detailed and formal versions can be found in the appendix.

## Preliminaries

### Mappings

For any *x* and *y*, we define ${{\,\mathrm{\delta }\,}}(x,y)$ to be 0 if $x=y$ and 1, otherwise, and we abbreviate $1-{{\,\mathrm{\delta }\,}}(x,y) =: {{\,\mathrm{\overline{\delta }}\,}}(x,y)$. We further abbreviate ${{\,\mathrm{\delta }\,}}(\phi (x),\phi (y))$ as ${{\,\mathrm{\delta }\,}}_\phi (x,y)$ for any function $\phi$. We may denote a pair (*x*, *y*) as $x\rightarrow y$ if it is referring to an assignment of *y* to *x* by some function and as *xy* if it refers to an arc in a network. We sometimes use the name of a function $\phi :X\rightarrow Y$ to refer to its set of pairs $\{x\rightarrow y\mid \phi (x)=y\}$ and we let ${\phi }\mid _{Z}:=\{(x\rightarrow y)\in \phi \mid x\in Z\}$ denote the *restriction* of $\phi$ to *Z*. We say $\phi (x)=\bot$ to indicate that $\phi$ is not defined for *x*. We denote the result of forcing $\phi (x)=y$ (whether or not *x* is mapped by $\phi$) as

$$\begin{aligned} {\phi }\left[ {x\rightarrow y}\right] := {\left\{ \begin{array}{ll} \phi \cup \{x\rightarrow y\} &{} \text {if}\, \phi (x)=\bot \\ {\left( \phi \setminus \{x\rightarrow \phi (x)\}\right) }\left[ {x\rightarrow y}\right] &{} \text {otherwise} \end{array}\right. } \end{aligned}$$

Finally, for sets *Z*, *X* and $Y\subseteq X$ and functions $\phi$ and $\psi$, we write $\psi \trianglelefteq \phi$ (and say that $\psi$ is a *subfunction* of $\phi$) if (a) $\phi :X\rightarrow Z$ and $\psi :Y\rightarrow Z$ and $\psi (x)\le \phi (x)$ for all $x\in Y$, or (b) $\phi :X\rightarrow 2^Z$ and $\psi :Y\rightarrow Z$ and $\psi (x)\in \phi (x)$ for all $x\in Y$, or (c) $\phi :X\rightarrow 2^Z$ and $\psi :Y\rightarrow 2^Z$ and $\psi (x)\subseteq \phi (x)$ for all $x\in Y$.

### Graphs and phylogenetic networks

In this work, we consider directed acyclic graphs (DAGs) *N* that may have a unique source $\rho _N$ called *root*. If the sinks (aka *leaves*) of *N* are labeled, we call *N* a *phylogenetic network*. We refer to the nodes and directed edges (arcs) of *N* by *V*(*N*) and *A*(*N*), respectively. The *underlying undirected graph* of *N* is the undirected graph on node-set *V*(*N*) that contains an edge $\{u,v\}$ if and only if *N* contains the arc (*u*, *v*). As we do not deal with mixed graphs, we use the term *uv* to refer to the arc from *u* to *v* or the undirected edge between *u* and *v*, depending on the context. We refer to the edge-set of an undirected graph *G* as *E*(*G*).

We denote the set of nodes of a DAG *N* with in-degree at least two by *R*(*N*) and we call such nodes *reticulations*. If $R(N)=\varnothing$, then *N* is called a *tree*. The result of, for each $v\in R(N)$ removing all but one of its incoming arcs is called a *switching* of *N* and $\mathcal {S}(N)$ denotes the set of all switchings of *N* (observe that all switchings are spanning trees). For each $v\in V(N)$, we denote the successors (or “children”) of *v* in *N* by $\hbox {Succ}_{N}{(v)}$ and its predecessors (or “parents”) by $\hbox {Pred}_{N}{(v)}$. If *N* contains a directed *u*-*w*-path, then we say that *w* is a *descendant* of *u* and *u* is an *ancestor* of *w* (denoted as $w\le _N u$ and $w<_N u$ if $u\ne w$). A set $Z\subseteq V(N)$ such that $u\not <_N w$ and $w\not <_N u$ for all $u,w\in Z$ is called an *anti-chain* in *N*. The *induced subgraph* *N*[*Z*] of a set $Z\subseteq V(N)$ is the result of removing all nodes $x\in V(N)\setminus Z$ from *N* (together with their incident arcs) and, for any $v\in V(N)$, the network $N_v:=N[\{w \mid w\le _N v\}]$ is called the subnetwork *rooted at* *v*.

## An alternative formulation of treewidth

In this section, we give an alternative definition of the *treewidth*, which allows to tackle the small parsimony problem for networks in a simpler and more intuitive way. Note that this alternative definition is known in the FPT community (Dendris et al. [24] call it the “support” of a vertex with respect to an ordering while, when referring to Arnborg [25]) and Mescoff et al. [26], call it “tree vertex separation”). However, since in these works its connection to treewidth is mostly touched in passing, we felt the need to prove it explicitly here.

Since tree decompositions are agnostic to edge directions, all results in this section are stated for undirected graphs *G* instead of networks *N*,. Keeping in mind that the framework is to be applied to phylogenetic networks, all examples will be made with DAGs while, for the sake of versatility, all results are stated for undirected graphs. The reader may simply ignore the edge directions in the examples as all undirected graphs will be underlying undirected graphs of some DAGs.

**Fig. 3:**
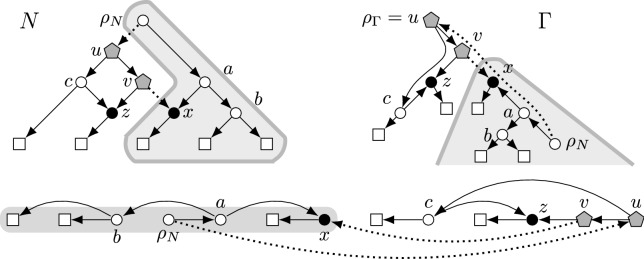
Example of a network *N* (left) with a linear order $$\sigma $$ of its nodes (below) as well as their canonical tree $$\Gamma ^\sigma $$ (right) whose arcs are not drawn (the arcs of *N* are drawn in their stead). Reticulations are black, leaves are boxes. For the first (wrt. $$\sigma $$) reticulation *x*, the set $$V(\Gamma ^\sigma _x)$$ is marked (gray area) and equals $$\sigma [1..x]$$ in this example. Further, the arcs in $$ {A}_{x}{(N)}$$ are dotted and the nodes in $$\hbox {YW}_{x}^{\Gamma }=\hbox {ZW}_{x}^{\sigma }$$ are gray pentagons. Note that $${x\mathop {\leadsto }\limits ^{N,\sigma } \rho _N}$$ but neither $${\rho _N\mathop {\leadsto }\limits ^{N,\sigma } x}$$ (since $$x\notin \sigma [1..\rho _N]$$) nor $${z\mathop {\leadsto }\limits ^{N,\sigma } x}$$ (since *x* is not weakly connected to *z* in $$N[\sigma [1..z]]$$)

For a linear ordering $\sigma$ of the nodes of an undirected graph *G* and any $x\in V(G)$, we write $y\le _\sigma x$ for all nodes *y* preceeding *x* in $\sigma$ (including *x* itself) and let $\sigma [1..x]$ denote the restriction of $\sigma$ to these nodes. We write ${x\mathop {\leadsto }\limits ^{G,\sigma } y}$ if *x* and *y* are connected in $G[\sigma [1..x]]$ (see Fig. 3 for an example). Note that ${\mathop {\leadsto }\limits ^{G,\sigma }}$ is a partial order on *V*(*G*). We consider nodes outside $\sigma [1..v]$ that have an edge to the parts of $\sigma [1..v]$ that are connected to *v* in $G[\sigma [1..v]]$. We denote these nodes by $\hbox {ZW}_{v}^{\sigma }$ and their number by $\hbox {zw}_{v}^{\sigma }$.

### Definition 1

Let $\sigma$ be a linear order of the nodes of an undirected graph *G* and let $v\in V(G)$. Then,

$$\begin{aligned} {\hbox {ZW}}_{v}^{\sigma } &:= \{u>_\sigma v \mid \exists _{w\in \sigma [1..v]} uw\in E(G) \wedge v\mathop {\leadsto }\limits ^{G,\sigma } w\} \\ &\quad {\text {and}} \ {\hbox {zw}}_{v}^{\sigma }:=|{\hbox {ZW}}_{v}^{\sigma }|. \end{aligned}$$

We abbreviate $\hbox {zw}{(\sigma )}:=\max _{v}\hbox {zw}_{v}^{\sigma }$ and $\hbox {zw}{(G)}:=\min _{\sigma }\hbox {zw}{(\sigma )}$ and we refer to the transitive reduction of the directed graph ${(V(G), \{uv \in V(G)^2 \mid u\mathop {\leadsto }\limits ^{G,\sigma } v\})}$ as the *canonical tree* $\Gamma ^\sigma$ of $\sigma$ for *G* (we will see below that $\Gamma ^\sigma$ is a rooted tree; see Fig. 3).

In the following, we say that a rooted tree $\Gamma$ on *V*(*G*) *agrees* with an undirected graph *G* if, for all $uv\in E(G)$ either $u<_\Gamma v$ or $v<_\Gamma u$. We also extend the definition of ${\mathop {\leadsto }\limits ^{G,\sigma }}$ to such trees by writing ${u \mathop {\leadsto }\limits ^{G,\Gamma } v}$ if *u* and *v* are connected in $G[\Gamma _u]$. In analogy to Definition 1, ${\mathop {\leadsto }\limits ^{G,\Gamma }}$ gives rise to a set $\hbox {YW}_{v}^{\Gamma }$ containing the nodes “above” *v* in $\Gamma$ that have a edge in *G* to a node “below” *v* in $\Gamma$.

### Definition 2

(see Fig. 3) Let *G* be an undirected graph and let $\Gamma$ agree with *G*. For each $v\in V(G)$, we define

$$\begin{aligned} \hbox {YW}_{v}^{\Gamma }:=\{u>_\Gamma v \mid \exists _{w\le _\Gamma v} uw\in E(G) \}&\text { and }&\hbox {yw}_{v}^{\Gamma }:=|\hbox {YW}_{v}^{\Gamma }|. \end{aligned}$$

Then, we abbreviate $\hbox {yw}(\Gamma ):=\max _{v}\hbox {yw}_{v}^{\Gamma }$ and $\hbox {yw}(G):=\min _{\Gamma }\hbox {yw}(\Gamma )$.

Note that the path *P* resulting from traversing $\sigma$ from right to left is a rooted tree agreeing with *G*. However, $\hbox {yw}{(P)}$ is expected to be large for this choice. Indeed, we can show that the most “refined” trees $\Gamma$ have the smallest $\hbox {yw}(\Gamma )$.

### Lemma 1

*Let* $\Gamma$* and* $\Gamma '$* be rooted trees agreeing with an undirected graph* *G and let* $\le _{\Gamma '}$* be a subset of* $\le _\Gamma$*, that is,* $x\le _{\Gamma '} y \Rightarrow x\le _\Gamma y$* for all *$x,y\in V(G)$.* Then,* $\text {yw}{(\Gamma ')}\le \text {yw}(\Gamma )$.

### Proof

Let $x\in V(G)$ and let $y\in \hbox {YW}_{x}^{\Gamma '}$ , that is, $y>_{\Gamma '} x$ and there is some $z\le _{\Gamma '} x$ with $yz\in E(G)$. Since $\le _\Gamma$ is a superset of $\le _{\Gamma '}$, we have $y>_\Gamma x\ge z$, implying $y\in \hbox {YW}_{x}^{\Gamma }$.$\square$

The following lemma proves a number of interesting properties relating $\sigma$ and $\Gamma ^\sigma$ such as $\Gamma ^\sigma$ being a rooted tree whose descendant relation is a refinement of $\le _\sigma$, culminating in the equality of $\hbox {ZW}_{x}^{\sigma }$ and $\hbox {YW}_{x}^{\Gamma \sigma }$ for all *x*.

### Lemma 2

*Let* $\sigma$* be a linear order of the nodes of a connected undirected graph* *G and let* $\Gamma ^\sigma$* be its canonical tree. Then,*

- for each *u* and *v* with $v\le _{\Gamma ^\sigma } u$, we have $v\le _\sigma u$,
- for each $u,v\in V(G)$, we have $v\le _{\Gamma ^\sigma } u$ if and only if ${u \mathop {\leadsto }\limits ^{G,\sigma } v}$,
- $$\Gamma ^\sigma $$ is connected,
- $\Gamma ^\sigma$ is rooted at the last vertex *r* of $\sigma$,
- $$\Gamma ^\sigma $$ is a tree,
- for all $uv\in E(G)$ with $v<_\sigma u$, we have $v<_{\Gamma ^\sigma } u$,
- $\Gamma ^\sigma$ agrees with *G*, and
- $$\hbox {YW}_{x}^{\Gamma \sigma }=\hbox {ZW}_{x}^{\sigma }$$ for all $$x\in V(G)$$.
- For each arc $xy\in A(\Gamma ^\sigma )$, $\Gamma ^\sigma _y$ contains a neighbor of *x* in *G*.
- Each $x\in V(G)$ has at most as many children in $\Gamma ^\sigma$ as it has neighbors in *G*.

### Proof

(a), (b): We show for all vertices *w* on a *u*-*v*-path *p* in $\Gamma ^\sigma$ that $w\le _\sigma u$ and ${u\mathop {\leadsto }\limits ^{G,\sigma } w}$. The base case $w=u$ holds trivially. For the induction step, let *q* preceed *w* in *p*. Since $\Gamma ^\sigma$ contains the arc *qw*, Definition 1 implies ${q \mathop {\leadsto }\limits ^{G,\sigma } w}$ and, since $q\le _\sigma u$ by induction hypothesis, $w\le _\sigma q\le _\sigma u$ and ${u\mathop {\leadsto }\limits ^{G,\sigma } w}$. For the reverse direction of (b), note that, by Definition 1, *uv* is an arc of the DAG of which $\Gamma ^\sigma$ is the transitive reduction.

(c),(d): Since *G* is connected, each $x\in V(G)$ has an *r*-*x*-path in $G=G[\sigma [1..r]]$, implying ${r\mathop {\leadsto }\limits ^{G,\sigma }x}$. Thus, (b) implies that $\Gamma ^\sigma$ is connected and rooted at *r*.

(e): To prove that $\Gamma ^\sigma$ is a tree, assume there is a vertex $x\in V(G)$ with two distinct parents *y* and *z* in $\Gamma ^\sigma$. Without loss of generality, let $y<_\sigma z$. By (b), ${y\mathop {\leadsto }\limits ^{G,\sigma } x}$ and ${z\mathop {\leadsto }\limits ^{G,\sigma } x}$, implying that $\sigma [1..y]$ contains a *y*-*x*-path $p_y$ in *G* and $\sigma [1..z]$ contains a *z*-*x*-path $p_z$ in *G*. Since $\sigma [1..y]\subsetneq \sigma [1..z]$ the concatenation of $p_z$ with (the reverse) of $p_y$ is a path in *G* whose nodes are in $\sigma [1..z]$. Thus, ${z\mathop {\leadsto }\limits ^{G,\sigma } y}$, implying $y\le _{\Gamma ^\sigma } z$ and, since $zx\in A(\Gamma ^\sigma )$, this contradicts $\Gamma ^\sigma$ being a transitive reduction.

(f): Note that ${u\mathop {\leadsto }\limits ^{G,\sigma } v}$, implying $v\le _{\Gamma ^\sigma } u$ by (b).

(g): For each $uv\in E(G)$, either $u<_\sigma v$, implying $u\le _{\Gamma ^\sigma } v$, or $v<_\sigma u$, implying $v\le _{\Gamma ^\sigma } u$ (both by (f)).

(h) “$\subseteq$”: Let $x\in V(G)$ and let $y\in \hbox {YW}_{x}^{\Gamma \sigma }$. By Definition 2, $y>_{\Gamma ^\sigma } x$ (implying $y>_\sigma x$ by (a)) and there is some $z\le _{\Gamma ^\sigma } x$ (implying $z\le _\sigma x$ by (a)) with $yz\in E(G)$. Then, by (b), ${x \mathop {\leadsto }\limits ^{G,\sigma } z}$. But then, $y\in \hbox {ZW}_{x}^{\sigma }$ by Definition 1.

(h) “$\supseteq$”: Let $x\in V(G)$ and let $y\in \hbox {ZW}_{x}^{\Gamma \sigma }$, that is, $x<_\sigma y$ and there is some $z\in \sigma [1..x]$ with ${x\mathop {\leadsto }\limits ^{G,\sigma } z}$ and $yz\in E(G)$. Then, $z \le _\sigma x <_\sigma y$. By (b), $z\le _{\Gamma ^\sigma } x$ and, by (f), $z\le _{\Gamma ^\sigma } y$. Thus, as $\Gamma ^\sigma$ is a tree (by (e)), *x* and *y* are not unrelated in $\Gamma ^\sigma$. Moreover, $y\nleq _\sigma x$ implies $y\nleq _{\Gamma ^\sigma } x$ by (b) and, thus, $x<_{\Gamma ^\sigma } y$. Together with $z\le _{\Gamma ^\sigma } x$ and $yz\in E(G)$, this implies $y\in \hbox {YW}_{x}^{\Gamma \sigma }$.

(i) By (b), *G* contains an *x*-*y*-path *p* whose vertices are in $\sigma [1..x]$ and, thus, ${x\mathop {\leadsto }\limits ^{G,\sigma }v}$ for all vertices *v* on *p*. We show $u\le _{\Gamma ^\sigma }y$ for all *u* on *p* except *x*, starting with the obvious $y\le _{\Gamma ^\sigma } y$. Then, this implies that the second vertex on *p*, which is a neighbor of *x* in *G*, is in $\Gamma ^\sigma _y$. Let $v\le _{\Gamma ^\sigma } y$ be a vertex on *p* and let *u* be the predecessor of *v* in *p*. If $u=x$ then we are done, so suppose $u\ne x$. Further, by (f), either $u<_\Gamma ^\sigma v \le _\Gamma ^\sigma y$, implying the claim directly, or $v<_\Gamma ^\sigma u$, implying that *u* is on an *x*-*v*-path in $\Gamma ^\sigma$. By (e) there is only one such path and it starts with $(x,y,\ldots )$ and, since $u\ne x$, this implies $u\le _\Gamma ^\sigma y$.

(j) is immediate from (i) combined with (e).$\square$

In order to show that $\hbox {zw}{(G)}$ and $\hbox {yw}(G)$ coincide, we need to “normalize” some aspects of the structure of agreeing trees. To this end, we use the following operation on rooted trees which can be interpreted as contracting a set of unwanted nodes upwards. Formally, for a rooted tree *T* and for $X\subset V(T)$ that does not contain the root *r* of *T*, we let $T\uparrow X$ denote the result of (1) replacing each arc *uv* with $uv\cap X=\{u\}$ with the arc *wv* where *w* is the lowest ancestor of *u* that is not in *X*, and (2) removing all nodes in *X* from *T*. Note that $T\uparrow X$ may have strictly larger out-degree than *T*, but does not create new ancestor-descendant relations.

### Observation 1

Let *T* be a tree, let $X\subseteq V(T)$ not contain its root, and let $u,v\in V(T\uparrow X)$ with $u \le _{T\uparrow X} v$. Then, $u \le _T v$.

### Lemma 3

*Let* $\Gamma$* be a rooted tree agreeing with an undirected graph* *G*.* Then, there is some rooted tree* $\Gamma ^*$* agreeing with* *G such that* $\hbox {yw}{(\Gamma ^*)}\le \hbox {yw}(\Gamma )$* and, for all* $u,v\in V(G)$* with* $v\le _{\Gamma ^*} u$*, we have* ${u\mathop {\leadsto }\limits ^{G,\Gamma ^*} v}$.

### Proof

**Fig. 4:**
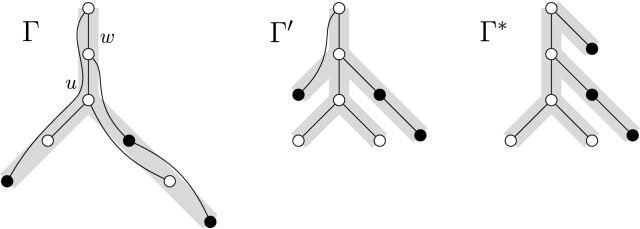
Example for the construction of $\Gamma '$ (middle) from $\Gamma$ (left) in Lemma 3. Repeated application yields $\Gamma ^*$ (right), for which ${v\le _{\Gamma ^*} u\Rightarrow u\mathop {\leadsto }\limits ^{G,\Gamma ^*} v}$. The rooted trees $\Gamma$, $\Gamma '$, and $\Gamma ^*$ are drawn with thick, gray lines. Thin, black lines are edges of *G*. For the indicated node *u*, the black nodes are in *X*, that is, they are below *u* in $\Gamma$ but not connected to *u* in $G[\Gamma _u]$

Let $u\in V(G)$ such that  We will modify $\Gamma$ into $\Gamma '$ with $\hbox {yw}{(\Gamma ')}\le \hbox {yw}(\Gamma )$ such that $\Gamma '$ agrees with *G* and the relation $\le _{\Gamma '}$ is a strict subset of $\le _\Gamma$. To this end, note that *u* has a parent *w* in $\Gamma$ as, otherwise, $G[\Gamma _u]=G$, implying $X=\varnothing$. Then, $\Gamma '$ results from $\Gamma$ by (see Fig. 4)

1. replacing $\Gamma$ by $\Gamma \uparrow (\Gamma _u\setminus X)$ and
2. dangling $$\Gamma _u\uparrow X$$ from *w*.

First, we show that $\Gamma '$ agrees with *G*. To this end, let $xy\in E(G)$ and let *x* and *y* be unrelated in $\Gamma '$. If neither *x* nor *y* are in $\Gamma _u$ then, by construction of $\Gamma '$, they are also unrelated in $\Gamma$, contradicting that $\Gamma$ agrees with *G*. So, without loss of generality, suppose $x\le _\Gamma u$. Since $xy\in E(G)$ and $\Gamma$ is a tree agreeing with *G*, we thus know that *u* and *y* are not unrelated in $\Gamma$. If $u<_\Gamma y$, then $w\le _\Gamma y$ and, thus, $x\le _{\Gamma '} y$. Thus, suppose $y\le _\Gamma u$. Clearly, if $x,y\in X$ or $x,y\notin X$, then *x* and *y* are also unrelated in $\Gamma$, contradicting its agreement with *G*. Thus, without loss of generality, suppose $x\in X$ and $y\notin X$, that is,  and ${u\mathop {\leadsto }\limits ^{G,\Gamma } y}$, contradicting $xy\in E(G)$.

Second, we show that $\le _{\Gamma '}$ is a strict subset of $\le _\Gamma$. To this end, let $xy \in A(\Gamma ')$ and assume towards a contradiction that $y\not <_\Gamma x$. Clearly, if $x\nleq _{\Gamma '} w$, then $xy\in A(\Gamma )$ contradicting $y\not <_\Gamma x$. Further, if $x=w$, then either $y\in X$ or *y* is a child of *w* in $\Gamma$, all of which imply $y<_\Gamma x$. Thus, $x<_{\Gamma '} w$. Since $xy\cap X=\{x\}$ or $xy\cap X=\{y\}$ contradicts $xy\in A(\Gamma ')$, we have $x,y\in X$ or $x,y\notin X$. But then, $y<_\Gamma x$ by Observation 1. Thus, $\le _{\Gamma '}$ is a subset of $\le _\Gamma$ and it is strict since we have $v\le _\Gamma u$ and $v\nleq _{\Gamma '} u$ for all $v\in X\ne \varnothing$.

Third, $\hbox {yw}{(\Gamma ')}\le \hbox {yw}(\Gamma )$ follows by Lemma 1.$\square$

### Lemma 4

*Let* $\Gamma$* be a tree agreeing with a graph* *G and let* *p be a non-empty path in* *G*.* Then, p contains a unique maximum* *u with respect to* $\Gamma$*, that is,* $v\le _\Gamma u$* for all vertices* *v of* *p*.

### Proof

Let *x* on *p* be maximal with respect to $\Gamma$ (that is, for all *z* on *p*, we have $x\not <_\Gamma z$) and assume towards a contradiction that there is another vertex $y\ne x$ on *p* that is maximal w.r.t. $\Gamma$. Without loss of generality, let *x* precede *y* in *p* and let $p_{xy}$ denote the unique *x*-*y*-subpath of *p*. Since $y\nleq _\Gamma x$, there is an edge $st\in E(G)$ on $p_{xy}$ with $s\le _\Gamma x$ and $t\nleq _\Gamma x$. Hence, $t\nleq _\Gamma s$. Further, $s\nleq _\Gamma t$ since, otherwise, the unique *t*-*s*-path in $\Gamma$ contains *x*, contradicting its maximality. But then $\Gamma$ does not agree with *G*.$\square$

### Lemma 5

*Let G be a graph. Then,* $\hbox {zw}{(G)}=\hbox {yw}(G)$.

### Proof

“$\ge$”: Let $\sigma$ be an ordering of *V*(*G*) such that $\hbox {zw}{(\sigma )}=\hbox {zw}{(G)}$. By Lemma 2(h), we have $\hbox {zw}{(\sigma )}=\hbox {yw}{(\Gamma \sigma )}$ for the canonical extension tree $\Gamma ^\sigma$ of $\sigma$. Thus, $\hbox {zw}{(G)}=\hbox {zw}{(\sigma )}=\hbox {yw}{(\Gamma \sigma )}\ge \hbox {yw}(G)$.

“$\le$”: Let $\Gamma$ be some rooted tree agreeing with *G* such that $\hbox {yw}(\Gamma )=\hbox {yw}(G)$. By Lemma 3, we may assume

1 $$\begin{aligned} {\forall _{u,v\in V(G)} u\le _\Gamma v \Rightarrow v\mathop {\leadsto }\limits ^{G,\Gamma } u.} \end{aligned}$$

Let $\sigma$ be any ordering of *V*(*G*) obtained by repeatedly picking and removing any leaf of $\Gamma$.$\square$

### Claim 1

For each $u,v\in V(G)$, we have $u\le _\Gamma v$ if and only if ${v\mathop {\leadsto }\limits ^{G,\sigma } u}$.

### Proof

First, note that all nodes below *v* in $\Gamma$ are chosen before *v*, so $\Gamma _v\subseteq \sigma [1..v]$.

“$\Rightarrow$”: Let $u\le _\Gamma v$, that is, $u\in \Gamma _v$, implying $u\le _\sigma v$. By (1), *v* is connected to *u* in $G[\Gamma _v]$ and, as $\Gamma _v\subseteq \sigma [1..v]$, also in $G[\sigma [1..v]]$.

“$\Leftarrow$”: Let *p* be a *v*-*u*-path in $G[\sigma [1..v]]$. By Lemma 4, *p* has a unique maximum *w* in $\Gamma$. Hence, $v\le _\Gamma w$ and, by “$\Rightarrow$”, we have $v\le _\sigma w$. Since *p* lives entirely in $G[\sigma [1..v]]$, that is, $V(p)\subseteq \sigma [1..v]$, we also have $w\le _\sigma v$. Thus, $v=w$ and, since $u\in V(p)$, we have $u\le _\Gamma w=v$ by maximality of *w*.$\square$

To prove the lemma, we show $\hbox {YW}_{x}^{\Gamma }\supseteq \hbox {ZW}_{x}^{\sigma }$ for each $x\in V(G)$. Let $y\in \hbox {ZW}_{x}^{\sigma }$, that is $y>_\sigma x$ and there is some $z\in \sigma [1..x]$ with $yz\in E(G)$ and ${x\mathop {\leadsto }\limits ^{G,\sigma } z}$. By Claim 1, $z\le _\Gamma x$. Further, as $yz\in E(G)$ and $\Gamma$ agrees with *G*, *y* and *z* are not unrelated in $\Gamma$ and, since $z\le _\Gamma x$, neither are *x* and *y*. Since $y<_\Gamma x$ implies $y<_\sigma x$ by Claim 1, contradicting $y>_\sigma x$, we conclude $x<_\Gamma y$. Together with $z\le _\Gamma x$ and $yz\in E(G)$, this implies $y\in \hbox {YW}_{x}^{\Gamma }$.

Having shown that the notion of $\hbox {zw}{(G)}$ and $\hbox {yw}(G)$ are equivalent, we can now turn our attention to the treewidth. In particular, we introduce (nice) tree-decompositions and use their properties to show that the treewidth of any undirected graph *G* equals $\hbox {yw}(G)$.

### Definition 3

**Fig. 5:**
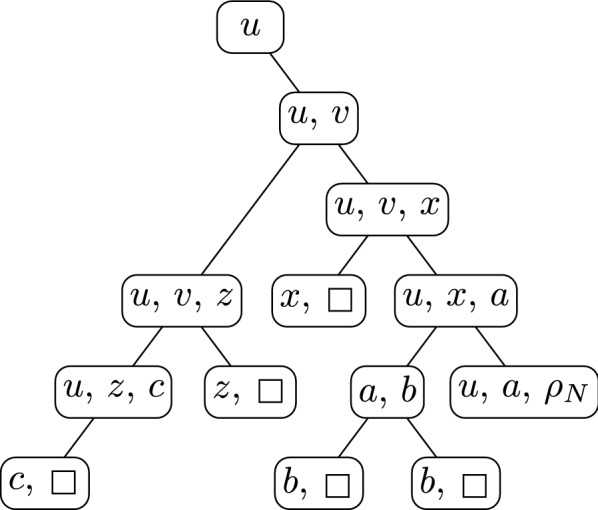
The tree decomposition $(\Gamma , B)$ for the network *N* given in Fig. 3 constructed in the “$\ge$”-part of Lemma 1. Leaves are represented by boxes instead of their names. Note that $(\Gamma ,B)$ is *not* a nice tree decomposition

(see Fig. 5) Let *G* be an undirected graph and let *T* be a rooted tree whose vertices are associated to subsets of *V*(*G*) by a function $B:V(T)\rightarrow 2^{V(G)}$ such that

- for each $uv\in E(G)$, there is some $x\in V(T)$ with $u,v\in B(x)$ and
- for each $v\in V(G)$, the nodes $x\in V(T)$ with $v\in B(x)$ are weakly connected in *T*.

We call (*T*, *B*) a *tree decomposition* of *G* and its *width* is $\hbox {tw}{(T,B)}:=\max _{x\in V(T)}\hbox {tw}_{x}{(T,B)}$ with $\hbox {tw}_{x}{(T,B)}:=|B(x)|-1$. We call $\hbox {tw}{(G)}:=\min _{T,B}\hbox {tw}{(T,B)}$ the *treewidth* of *G*.

We call (*T*, *B*) *nice* if *T* is binary and all $x\in V(T)$ fall into one of the following categories

- **“leaf”:** *x* is a leaf of *T* and $B(x)=\varnothing$,
- **“root”:** *x* is the root of *T* and $B(x)=\varnothing$,
- **“introduce** *v*”: *x* has a single child *y* in *T* and $B(y)=B(x)-v$,
- **“forget** *v*”: *x* has a single child *y* in *T* and $B(x)=B(y)-v$,
- **“join”**: *x* has two children *y* and *z* and $B(x)=B(y)=B(z)$.

As stated at the beginning of the section, recall that, while tree decompositions are defined for undirected graphs, we may talk about tree decompositions of DAGs, meaning tree decompositions of their underlying undirected graphs. Note that all graphs *G* have a nice tree decomposition with $|V(T)|\in O(\hbox {tw}{(G)}\cdot |G|)$ and width $\hbox {tw}{(G)}$ [27]. Further, since all bags of (*T*, *B*) containing a vertex *v* of *G* are connected, we can observe the following.

### Observation 2

Let (*T*, *B*) be a nice tree decomposition for an undirected graph *G* and let $v\in V(G)$. Then, *T* contains a single “forget *v*”-node *x* and $y<_T x$ for all *y* with $v\in B(y)$.

### Proposition 1

*Let* *G be an undirected graph. Then,* $\hbox {yw}(G)=\hbox {tw}{(G)}$.* Further, given a tree decomposition* (*T*, *B*)* for* *G*,* we can compute a tree* $\Gamma$* agreeing with* *G such that* $\hbox {yw}(\Gamma )=\hbox {tw}{(T,B)}$* in linear time*.

### Proof

“$\le$”: Let (*T*, *B*) be a nice tree decomposition for *G* of width $\hbox {tw}{(G)}$ and let $F\subset V(T)$ denote the set of all “forget”-nodes in *T* (noting that *F* contains the root of *T*). We define $\Gamma$ as the transitive reduction of $(F,>_T\cap (F\times F))$.^2^ Note that $u\le _\Gamma v \iff u\le _T v$ for all $u,v\in F$ and, by Observation 2, $V(\Gamma )=F=V(G)$.

First, we show that $\Gamma$ agrees with *G*. To this end, let $uv\in E(G)$ and let $f_u,f_v\in F$ denote the unique “forget *u*” and “forget *v*”-nodes in *T*, which are distinct since *T* is nice. By Definition 3(a), there is a node $q\in V(T)$ with $u,v \in B(q)$ and, by Observation 2, $q<_T f_u,f_v$. Thus, $f_u$ and $f_v$ are not unrelated in *T* and, thus, neither in $\Gamma$.

Second, we show for all $v\in \Gamma$ and the unique “forget *v*”-node $f_v$ in *T* that $\hbox {YW}_{v}^{\Gamma }\subseteq B(f_v)$. Let $u\in \hbox {YW}_{v}^{\Gamma }$, that is, $u>_\Gamma v$ and there is some $w\le _\Gamma v$ such that $uw\in E(G)$ (note that $w\ne u$ but $w=v$ is possible). Let $f_u$ and $f_w$ be the unique “forget *u*” and “forget *w*”-nodes in *T*, which are distinct since *T* is nice. Then, $w\le _\Gamma v <_\Gamma u$ and, since $f_u,f_w\in F$, we also have $f_w\le _T f_v <_T f_u$. Since $uw\in E(G)$, Definition 3(a) implies that there is a node *q* of *T* with $u,w \in B(q)$ and, by Observation 2, $q<_T f_u,f_w$. Then, by Definition 3(b), $u\in B(x)$ for all *x* with $q\le _T x<_T f_u$ and, since $q<_T f_w \le _T f_v <_T f_u$, we have $u\in B(f_v)$. As *u* was chosen arbitrary, we conclude $\hbox {YW}_{v}^{\Gamma }\subseteq B(f_v)$. Hence, $\hbox {yw}(G)\le |\hbox {YW}_{v}^{\Gamma }|\le |B(f_v)|$ and, since $f_v$ has a child *x* with $B(x)=B(f_v)\cup \{v\}$, we know $|B(f_v)|=|B(x)| - 1\le \hbox {tw}{(T,B)}=\hbox {tw}{(G)}$.

“$\ge$”: Let $\Gamma$ be a tree with $\hbox {yw}(\Gamma )=\hbox {yw}(G)$ that agrees with *G*. For all $u\in V(G)$, we define $B(u):=\hbox {YW}_{u}^{\Gamma }\cup \{u\}$ and show that $(\Gamma ,B)$ is a tree-decomposition for *G* noting that its width is $\hbox {yw}(\Gamma )=\hbox {yw}(G)$ (see example in Fig. 5).

First, to prove Definition 3(a), let $uv\in E(G)$. Since $\Gamma$ agrees with *G*, either $u<_\Gamma v$ or $v<_\Gamma u$. Without loss of generality, suppose the latter. Then, $u\in \hbox {YW}_{v}^{\Gamma }$ by Definition 2 (using $w=v$), implying that $uv\in B(v)$.

Second, let $u,v\in V(G)$ be distinct such that $u\in B(v)=\hbox {YW}_{v}^{\Gamma }\cup \{v\}$, implying $u\in \hbox {YW}_{v}^{\Gamma }$ since $u\ne v$. By Definition 2, there is some $w\le _\Gamma v$ such that $uw\in E(G)$ and $v<_\Gamma u$, implying that $\Gamma$ contains a unique *u*-*v*-path *p*. To show Definition 3(b), it suffices to prove $u\in B(x)$ for all $x\in V(p)$ (since *v* has been chosen arbitrarily, a path with these properties exists for all $v'$ with $u\in B(v')$, so they all contain the node *u* and are, thus, connected). For $x=u$ this follows by definition of *B*(*u*). Otherwise, $x<_\Gamma u$ since $x\in V(p)$. But then, $w\le _\Gamma v\le _\Gamma x <_\Gamma u$ and $uw\in E(G)$, implying $u\in \hbox {YW}_{x}^{\Gamma }\subseteq B(x)$.$\square$

## Parsimony

**Fig. 6:**
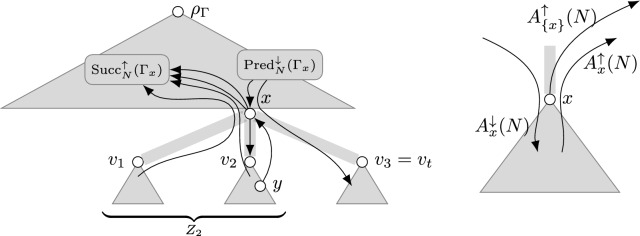
A tree $\Gamma$ is depicted in gray and some arcs of *N* are depicted in black. Recall that *t* is the number of children of *x* and $Z_i:=\bigcup _{1\le j\le i}\Gamma _{v_j}$. Note that $x\in \hbox {Succ}_{N}^{\uparrow }{(Z_2)}\setminus \hbox {Succ}_{N}^{\uparrow }{(\Gamma _x)}$ since *x* is an ancestor of a node of $\Gamma _{v_2}$ in *N*. Note that *x* is a reticulation of *N* with parents *y* (drawn) and *z* (not drawn) with $y<_\Gamma v_2<_\Gamma x<_\Gamma z$. Thus, $z\in \hbox {Pred}_{N}^{\downarrow }{(x)}$ but $y\in \hbox {Pred}_{N}^{\uparrow v_2}{(x)}\subseteq \hbox {Pred}_{N}^{\uparrow }{(x)}$. Finally, note that $\hbox {YW}_{x}^{\Gamma }=\hbox {Pred}_{N}^{\downarrow }{(\Gamma _x)}\cup \hbox {Succ}_{N}^{\uparrow }{(\Gamma _x)}$ and $\bigcup _{i\le t}\hbox {YW}_{{v_i}}^{\Gamma }\subseteq \hbox {YW}_{x}^{\Gamma }\uplus \{x\}$

Notation Large parts of this work are in context of a rooted tree $\Gamma$ on the node set *V*(*N*) of a given phylogenetic network *N* (see Fig. 6). Specifically for the tree $\Gamma$, we permit ourselves to abbreviate $V(\Gamma _x)$ to $\Gamma _x$ to increase readability. In such context, we additionally define the following sets for any nodes $y,z\in V(N)$: $\hbox {Pred}_{N}^{\uparrow y}{(z)}:=\hbox {Pred}_{N}{(z)}\cap \Gamma _y$ and $\hbox {Pred}_{N}^{\downarrow y}{(z)}:=\hbox {Pred}_{N}{(z)}\setminus \Gamma _y$ denote the respective *predecessors* of *z* in *N* that are or are not in $\Gamma _y$. Likewise, $\hbox {Succ}_{N}^{\uparrow y}{(z)}:=\hbox {Succ}_{N}{(z)}\cap \Gamma _y$ and $\hbox {Succ}_{N}^{\uparrow y}{(z)}:=\hbox {Succ}_{N}{(z)}\setminus \Gamma _y$ denote the respective *successors* of *z* in *N* that are or are not in $\Gamma _y$ – note that the arrow in the notation indicates the direction of the arc between *z* and the members of the set when drawing $\Gamma$ top-down. If $z=y$, we drop *y* and simply write $\hbox {Pred}_{N}^{\downarrow }{(z)}$, $\hbox {Pred}_{N}^{\uparrow }{(z)}$, $\hbox {Succ}_{N}^{\uparrow }{(z)}$, and $\hbox {Succ}_{N}^{\uparrow }{(z)}$. We also abbreviate $\hbox {Pred}_{N}^{\downarrow }{(z)}\cap R(G)=:\hbox {Pred}_{N}^{R\downarrow }{(z)}$ and $\hbox {Succ}_{N}^{\uparrow }{(z)}\cap R(G) =: \hbox {Succ}_{N}^{R\uparrow }{(z)}$ as well as $\hbox {Pred}_{N}^{\downarrow }{(z)}\setminus R(G) =: \hbox {Pred}_{N}^{T\downarrow }{(z)}$ and $\hbox {Succ}_{N}^{\uparrow }{(z)}\setminus R(G) =: \hbox {Succ}_{N}^{T\uparrow }{(z)}$. All these functions generalize to sets $Z\subseteq V(N)$ (for example, $\hbox {Pred}_{N}{(Z)} := \bigcup _{z\in Z}\hbox {Pred}_{N}{(z)}\setminus Z$). Further, for any $X\subseteq V(N)$, we define the sets of arcs of *N*

- from a node $u\in X$ to any ancestor of *u* in $\Gamma$ as $A^{\uparrow }_{X}{(N)}:=\{uw\in A(N) \mid u\in X \wedge u<_\Gamma w\}$ and
- to a node $u\in X$ from any ancestor of *u* in $\Gamma$ as $A^{\downarrow }_{X}{(N)}:=\{uw\in A(N) \mid w\in X \wedge w<_\Gamma u\}$.

For brevity, we abbreviate $A_{X}{(N)}:=A^{\uparrow }_{X}{(N)}\cup A^{\downarrow }_{X}{(N)}$, $A^{\uparrow }_{v}{(N)}:=A^{\uparrow }_{{\Gamma _v}}{(N)}$, $A^{\downarrow }_{v}{(N)}:=A^{\downarrow }_{{\Gamma _v}}{(N)}$, and ${A}_{v}{(N)}:={A}_{{\Gamma _{v}}}{(N)}$.

Introduction to Parsimony Given states of a character, observed in extant species, as well as a species phylogeny, the small parsimony problem asks to infer states of the same character for all ancestral species such as to minimize the “parsimony score” of this assignment. This problem comes in three flavors called “hardwired”, “softwired”, and “parental” parsimony. Throughout this section, let *C* be a fixed finite set (a “character”). For convenient use of the $\trianglelefteq$-relation, let *C* be an anti-chain (that is, for each $x,y\in C$, we have $x\le y$ only if $x=y$). Formally, for a phylogeny *N* and a function $\phi :V(N)\rightarrow 2^C$, we define the hardwired and softwired parsimony score as

$$\begin{aligned} {{\,\mathrm{par}\,}}^H_N(\phi ) & := \min _{\psi :V(N)\rightarrow C,\;\psi \trianglelefteq \phi } \sum \limits _{uv\in A(N)} {{\,\mathrm{\delta }\,}}_\psi (u,v)\\ {{\mathrm{par}\,}}^S_N(\phi )& := \min _{\begin{array}{c} \psi :V(N)\rightarrow C,\;\psi \trianglelefteq \phi \\ T\in \mathcal {S}(N) \end{array}}\sum \limits _{uv\in A(T)} {{\,\mathrm{\delta }\,}}_\psi (u,v). \end{aligned}$$

The “parental parsimony” is defined using “parental trees” but, in this work, we use the equivalent formulation using lineage functions [12].

### Definition 4

A *lineage function* for a phylogeny *N* is any function $f:V(N)\rightarrow 2^{C}$. The *cost* of *f* is $\hbox {cost}_{(f)}:=\sum _{v\in V(N)}\hbox {cost}_{f}{(v)}$ where

$$\begin{aligned} \hbox {cost}_{f}{(v)}:= |f(v)\setminus {\bigcup \limits _{u\in \hbox {Pred} {{(v)}}}}f(u)| + {\left\{ \begin{array}{ll} -1 &{} \text {if}\,v=\rho _N\hbox { and }|f(v)|=1\\ 0 &{} \text {if}\,v\ne \rho _N\hbox { and }|f(v)| \le \sum _{u\in \hbox {Pred}{(v)}}|f(u)|\\ \infty &{} \text {otherwise} \end{array}\right. } \end{aligned}$$

Given *N* and a function $\phi :V(N)\rightarrow 2^{C}$, we denote the set of all lineage functions *f* on *N* with $f\trianglelefteq \phi$ as $\mathcal {LF}_{N,\phi }$. Finally, the *parental parsimony score* is

2 $$\begin{aligned} {{\,\mathrm{par}\,}}^P_N(\phi ) := \min _{f\in \mathcal {LF}_{N,\phi }}\hbox {cost}_{(f)} \end{aligned}$$

For each of the presented variants, we give a dynamic programming formulation using a given tree $\Gamma$ that agrees with the undirected graph *G* underlying the input network and corresponds to Lemma 3, that is, each non-leaf *x* of $\Gamma$ has a child *v* with $x\in \hbox {YW}_{v}^{\Gamma }$. The running time of the resulting algorithm will depend on the width $\hbox {yw}(\Gamma )$ of $\Gamma$ (recalling that $\hbox {yw}(\Gamma )$ coincides with the treewidth of *G* for optimal $\Gamma$).

As stated in the introduction, in this paper we focus on the case of analyzing a specific position in the genome. Since the function $\phi$ can associate several states to a same leaf, our definition permits to describe polymorphism in a population. While in our current formulation the algorithms “choose” an optimal state to associate to each leaf, the parental parsimony can be easily modified to explain *all* states of each leaf at the end of the run. This allows keeping the information on polymorphism in all steps of the algorithm (see “Parental parsimony”). Note also that $\phi$ can associate information to internal nodes, thus permitting the user to impose restrictions on the states associated to ancestral species.

In the presentation of the dynamic programming, a table entry $Q^y_x[z]$ means that *x* and *y* are considered fix for this table and *z* is a variable index. Further, tables $Q^{y_1}_{x_1}$ and $Q^{y_2}_{x_2}$ are independent of one another, allowing an implementation to forget $Q^{y_1}_{x_1}$ if it is no longer needed, even if $Q^{y_2}_{x_2}$ still is. In the following, for an anti-chain *Y* in $\Gamma$ and a class $\mathcal {G}$ of subnetworks of *N*, a *Y*-*substitution system* of $\mathcal {G}$ is a series of subnetworks $(N^y)_{y\in Y}$ of *N* such that, for all $N'\in \mathcal {G}$, the digraph $(V(N), (A(N')\setminus \bigcup _{y\in Y}{A}_{y}{(N')})\cup \bigcup _{y\in Y}{A}_{y}{(N^{y})})$ is also in $\mathcal {G}$. Roughly, we can “swap out” the arcs in ${A}_{y}{(N')}$ for ${A}_{y}{(N^{y})}$ for each $y\in Y$ without loosing membership in $\mathcal {G}$. Note that the $N^y$ are not necessarily distinct, so a trivial *Y*-substitution system for $\{N'\}$ would be $(N')_{y\in Y}$. The formulations are based on the following lemma about independent sub-solutions, showing that an optimal solution $(S,\psi )$ for a sub-network (of *G*) “below” an anti-chain *Z* in $\Gamma$ is also optimal on any sub-network “below” an anti-chain *Y* in $\Gamma$ that is itself “below” *Z* (among all solutions with $\psi$’s behavior on $\bigcup _{y\in Y}\hbox {YW}_{y}^{\Gamma }$).

### Lemma 6

**Fig. 7:**
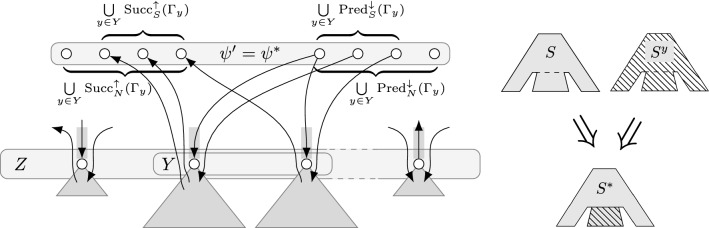
Lemma 6 proves that any solution $(S,\psi )$ that is optimal on sub-trees rooted at *Z* in $\Gamma$ must also be optimal (among all solutions with $\psi$’s behavior on $\bigcup _{y\in Y}\hbox {YW}_{{y}}^{\Gamma }$ (gray box on top)) on all sub-trees of $\Gamma$ that are rooted below *Z* (at *Y*). That is, no solution $(S^y,\psi _y)$ can be better than $(S,\psi )$ on the sub-network induced by $\Gamma _y$ for any $y\in Y$. To prove this, a new solution $(S^*,\psi ^*)$ is constructed by replacing the sub-solution of $(S,\psi )$ below *Y* by the sub-solutions $(S^y,\psi _y)$ below *Y*

 (see Fig. 7) *Let* $Y,Z\subseteq V(N)$* be anti-chains in* $\Gamma$* such that* $Y\subseteq \bigcup _{z\in Z}\Gamma _z$.* Let* $\mathcal {G}$* be a class of subnetworks of* *N and let* $S\in \mathcal {G}$* and* $\psi :V(N)\rightarrow C$* such that (a)* $\sum _{z\in Z}\sum _{uw\in {A}_{z}{(S)}}{{\,\mathrm{\delta }\,}}_\psi (u,w)$* is minimum among all such* *S and* $\psi$.* Let *$(S^y)_{y\in Y}$* be a* *Y-substitution system for* $\mathcal {G}$* and let* $\psi _y:V(N)\rightarrow C$* for each* $y\in Y$* such that (b)* $\psi _y$* and* $\psi$* coincide on* $\hbox {YW}_{y}^{\Gamma }$.* Then,*

$$\begin{aligned} \sum _{y\in Y}\sum \limits _{uw\in A_y({S^y})}{{\,\mathrm{\delta }\,}}_{\psi _y}(u,w) \ge \sum _{y\in Y}\sum \limits _{uw\in A_y({S})}{{\,\mathrm{\delta }\,}}_{\psi }(u,w). \end{aligned}$$

### Proof

Towards a contradiction, assume that the lemma is false. We construct $\psi ^*:V(N)\rightarrow C$ with

$$\begin{aligned} \psi ^*(u) = {\left\{ \begin{array}{ll} \psi _y(u) &{} \text {if}\,u\in \Gamma _y\hbox { for any }y\in Y\\ \psi (u) &{} \text {otherwise} \end{array}\right. } \end{aligned}$$

Note that $\psi ^*$ and $\psi$ coincide with $\psi _y$ on $\hbox {YW}_{y}^{\Gamma }$ for all $y\in Y$. Thus, ${{\,\mathrm{\delta }\,}}_{\psi ^*}(u,w) = {{\,\mathrm{\delta }\,}}_{\psi _y}(u,w)$ if $uw\in {A}_{y}{(S^{*})}$ for any $y\in Y$ and ${{\,\mathrm{\delta }\,}}_{\psi ^*}(u,w) = {{\,\mathrm{\delta }\,}}_{\psi }(u,w)$, otherwise. Further, we construct a digraph $S^*:=(V(N), (A(S)\setminus \bigcup _{y\in Y} {A}_{y}{(S)})\cup \bigcup _{y\in Y} {A}_{y}{(S^{y})})$ which is in $\mathcal {G}$ since $(S^y)_{y\in Y}$ is a *Y*-substitution system for $\mathcal {G}$. Since all $S^y$ are subnetworks of *N*, we know that $\Gamma$ agrees with $S^*$. Furthermore, since $Y\subseteq \bigcup _{z\in Z}\Gamma _z$, we know that each $y\in Y$ has a $z\in Z$ with $y\le _\Gamma z$. Thus,

$$\begin{aligned} \sum _{z\in Z}\;\;{\sum _{uw\in {A}_{z}{(S^{*})}}}{{\,\mathrm{\delta }\,}}_{\psi ^*}(u,w)&= \sum _{z\in Z}\sum _{v\in \Gamma _z} \sum \limits _{uw\in {A}_{\{v\}}{(S^{*})}}{{\,\mathrm{\delta }\,}}_{\psi ^*}(u,w)\\&= \sum _{z\in Z}\sum _{\begin{array}{c} v\in \Gamma _z\\ v\notin \bigcup _{y\in Y}\Gamma _y \end{array}}\sum \limits _{uw\in A_\{v\}({S^*})}{{\,\mathrm{\delta }\,}}_{\psi ^*}(u,w) + \sum _{y\in Y}\sum \limits _{uw\in A_\{v\}({S^*})}{{\,\mathrm{\delta }\,}}_{\psi ^*}(u,w)\\&= \sum _{z\in Z}\sum _{\begin{array}{c} v\in \Gamma _z\\ v\notin \bigcup _{y\in Y}\Gamma _y \end{array}}\sum \limits _{uw\in A_y({S})}{{\,\mathrm{\delta }\,}}_{\psi }(u,w) + \sum _{y\in Y}\sum _{uw\in A_y({S^y})}{{\,\mathrm{\delta }\,}}_{\psi _y}(u,w)\\&{\mathop {<}\limits ^{\text {assumption}}} \sum _{z\in Z}\sum _{\begin{array}{c} v\in \Gamma _z\\ v\notin \bigcup _{y\in Y}\Gamma _y \end{array}} {\sum _{uw\in {A}_{\{v\}}{(S)}}}{{\,\mathrm{\delta }\,}}_{\psi }(u,w) + \sum _{y\in Y}\sum \limits _{uw\in A_y({S})}{{\,\mathrm{\delta }\,}}_{\psi }(u,w)\\&= \sum _{z\in Z}\sum \limits _{uw\in A_z({S})}{{\,\mathrm{\delta }\,}}_{\psi }(u,w) \end{aligned}$$

contradicting optimality of *S* and $\psi$ (that is, Lemma 6(a)) since $S^*\in \mathcal {G}$.$\square$

### Hardwired parsimony

To compute the hardwired parsimony score at a node *v* of *N*, we require knowledge of the character assigned to *v* and its neighbors. For all $$u\in \hbox {YW}_{v}^{\Gamma }$$, we thus “guess” the character $$\psi (u)$$ assigned to *u* by an optimal assignment. In our dynamic programming, we scan $$\Gamma $$ bottom-up, computing a table entry $$T^{\mathcal {HW}}[{x,\psi }]$$ for each $$x\in V(\Gamma )=V(N)$$ and each $$\psi :\hbox {YW}_{x}^{\Gamma }\rightarrow C$$, containing the parsimony cost incurred by all arcs in $$ {A}_{x}{(N)}$$, assuming that all nodes in $$\hbox {YW}_{x}^{\Gamma }$$ receive their characters according to $$\psi $$. Note that $$ {A}_{x}{(N)}=\bigcup _i {A}_{{v_{i}}}{(N)}\cup {A}_{\{x\}}{(N)}$$, where the $$v_i$$ are the children of *x* in $$\Gamma $$. Thus, $$T^{\mathcal {HW}}[{x,\psi }]$$ can be calculated as follows.

#### Definition 5

Let $\Gamma$ be a tree that agrees with *N*, let $x\in V(N)$ and let $\psi _x:\hbox {YW}_{x}^{\Gamma }\rightarrow C$ with $\psi _x\trianglelefteq \phi$. Let $v_1,v_2,\ldots ,v_t$ denote the children of *x* in $\Gamma$ ($t=0$ if *x* is a leaf). Then, we define a table entry

3 $$\begin{aligned} T^{\mathcal {HW}}[{x,\psi _x}] := \min _{c_x\in \phi (x)} \left( \sum \limits _{1\le i\le t} T^{\mathcal {HW}}[{v_i,{{\psi _x}\left[ {x\rightarrow c_x}\right] }\mid _{\hbox {YW}_{{v_i}}^{\Gamma }}}] + {\sum _{z\in \hbox {Pred}_{N}^{\downarrow }{(x)}\cup \hbox {Succ}_{N}^{\uparrow }{(x)}}} {{\,\mathrm{\delta }\,}}(c_x,\psi _x(z)) \right) \end{aligned}$$

#### Lemma 7

*Let* $x\in V(N)$* and let* $\psi _x:\hbox {YW}_{x}^{\Gamma }\rightarrow C$* with* $\psi _x\trianglelefteq \phi$*. Let* $\psi :V(N)\rightarrow C$* with* $\psi _x\trianglelefteq \psi \trianglelefteq \phi$* such that* $\psi$* minimizes* $\sum _{uw\in {A}_{x}{(N)}}{{\,\mathrm{\delta }\,}}_\psi (u,w)$.* Then,*

$$\begin{aligned} T^{\mathcal {HW}}[{x,\psi _x}] = \sum \limits _{uw\in A_x({N})}{{\,\mathrm{\delta }\,}}_\psi (u,w) \end{aligned}$$

*Proof Sketch.* For “$$\ge $$”, we construct a mapping $$\psi '$$ from mappings $$\psi _i$$ that are optimal on $$ {A}_{{v_{i}}}{(N)}$$ among all mappings with $$\psi _i(x):=c_x$$. This is possible since all such $$\psi _i$$ coincide with $$\psi '$$ and $$\psi _x$$ on $$\hbox {YW}_{x}^{\Gamma }$$. By induction hypothesis, the cost of $$\psi '$$ on $$ {A}_{x}{(N)}$$ is $${\sum _{1\le i\le t}T^{\mathcal {HW}}[{v_i,{\psi '}\mid _{\hbox {YW}_{{v_i}}^{\Gamma }}}] + \sum _{uw\in {A}_{\{x\}}{(N)}}{{\,\mathrm{\delta }\,}}_{\psi '}(u,w)}$$. Then, “$$\ge $$” follows from optimality of $$\psi $$ on $$ {A}_{x}{(N)}$$.

For “$$\le $$”, it suffices to show that the cost of $$\psi $$ on $$ {A}_{x}{(N)}$$ is equal to the result of setting $$c_x:=\psi (x)$$ in the right hand side of (3) (which is a valid choice for the minimum since $$\psi (x)\in \phi (x)$$). First, the cost of $$\psi $$ on $$ {A}_{{v_{i}}}{(N)}$$ is $$T^{\mathcal {HW}}[{v_i,{\psi }\mid _{\hbox {YW}_{{v_i}}^{\Gamma }}}]$$ by independence of sub-solutions and the induction hypothesis. Second, the cost of $$\psi $$ on $$A^{\downarrow }_{\{x\}}{(N)}$$ is $$\sum _{z\in \hbox {Pred}_{N}^{\downarrow }{(x)}}{{\,\mathrm{\delta }\,}}(c_x,\psi _x(z))$$ and the cost of $$\psi $$ on $$A^{\uparrow }_{\{x\}}{(N)}$$ is $$\sum _{z\in \hbox {Succ}_{N}^{\uparrow }{(x)}}{{\,\mathrm{\delta }\,}}(c_x,\psi _x(z))$$ since $$\psi $$ and $$\psi _x$$ coincide on $$\hbox {YW}_{x}^{\Gamma }$$$$\square $$.

In order to solve the hardwired parsimony problem given *N*, $\phi$ and $\Gamma$, all we have to do is compute $T^{\mathcal {HW}}[{x,\psi _x}]$ for each *x* bottom-up in $\Gamma$ and each of the (at most) $|C|^{|\hbox {YW}_{x}^{\Gamma }|}$ many choices of $\psi _x:\hbox {YW}_{x}^{\Gamma }\rightarrow C$ with $\psi _x\trianglelefteq \phi$. Then, by Lemma 7, the hardwired parsimony score of *N* with respect to $\phi$ can be read from $T^{\mathcal {HW}}[{\rho _\Gamma ,\varnothing }]$. To compute $T^{\mathcal {HW}}$, the sum over the children of *x* for all $x\in V(N)$ in (3) can be computed in amortized *O*(|*A*(*N*)|) time and, with a bit of bookkeeping, it is possible to maintain the value of the second sum in (3) in *O*(|*A*(*N*)|) amortized time per choice of $\psi$. Then the following holds:

#### Theorem 1

*Given a network* *N, some *$\phi :V(N)\rightarrow 2^{C}$* and a tree* $\Gamma$* agreeing with* *N, the hardwired parsimony score of *$(N,\phi )$* can be computed in *$O(|C|^{\hbox {yw}(\Gamma )+1}\cdot |A(N)|)$* time.*

Proposition 1 lets us turn tree decompositions of *N* into trees $\Gamma$ agreeing with *N*, allowing us to replace $\hbox {yw}(\Gamma )$ by $\hbox {tw}{(N)}$, incurring an additional running time of $|N|\cdot 2^{O(\hbox {tw}{(N)}^3)}$ [13].

#### Corollary 1

*Let *$(N,\phi )$* be an instance of* Hardwired Parsimony.* Let *$t\ge \hbox {tw}{(N)}$* and let* *T be the time in which a width-t tree decomposition of N can be computed. Then, the hardwired parsimony score of *$(N,\phi )$* can be computed in* $O(T+ |C|^{t+1}\cdot |A(N)|)$ *time.*

### Softwired parsimony

In contrast to the hardwired parsimony score, where the computation of the cost of the incident edges of a node *x* only required knowledge of the characters assigned to neighbors of *x*, computing the *softwired* score additionally requires knowledge of which parent of *x* remains a parent in the sought switching. A table entry $$T^{\mathcal {SW}}[{x, \ldots }]$$ contains the smallest combined cost of all arcs in $$ {A}_{x}{(S)}$$ for a switching *S* of *N* minimizing this cost. To be able to compute an entry for $$x\in V(N)$$, we not only need to “guess” $$\psi _x$$ but, additionally, some representation of the switching *S*. In particular, in *S*, no child of *x* may have another parent than *x*. However, since children of *x* in *N* may be above *x* in $$\Gamma $$, we have to “guess” which children of *x* in *N* are still children of *x* in *S*. Such a guess manifests itself as an additional index $$R^x$$ of the dynamic programming table (note that we clearly only have to store this information for children of *x* that are reticulations). Indeed, this information has to be stored for all nodes considered below *x* who still have children in $$\hbox {YW}_{x}^{\Gamma }$$. Thus, we index our DP-table also by a subset $$R^x\subseteq \hbox {YW}_{x}^{\Gamma }\cap R(N)$$ containing a reticulation $$r\in R(N)$$ if and only if $$\Gamma _x$$ contains a parent *v* of *r* and *vr* is an arc of an optimal switching *S* for $$N[\Gamma _x\cup \hbox {YW}_{x}^{\Gamma }]$$.

#### Definition 6

Let $\Gamma$ be a tree that agrees with *N*, let $x\in V(N)$, let $\psi _x:\hbox {YW}_{x}^{\Gamma }\rightarrow C$ with $\psi _x\trianglelefteq \phi$, and let $R^x\subseteq \hbox {Succ}_{N}^{R\uparrow }{(\Gamma _x)}$. Let $v_1, v_2,\ldots , v_t$ denote the children of *x* in $\Gamma$ ($t=0$ if *x* is a leaf in $\Gamma$). Then, set

4 $$\begin{aligned} T^{\mathcal {SW}}[{x, \psi _x, R^x}]&:= \min _{c_x\in \phi (x)} \min _{R^*\subseteq R^x\cap \hbox {Succ}_{N}^{R\uparrow }{(x)}} \sum \limits _{r\in R^*\cup \hbox {Succ}_{N}^{T\uparrow }{(x)}}{{\,\mathrm{\delta }\,}}(c_x,\psi _x(r)) \;\; + \nonumber \\&\min \left\{ \begin{array}{ll} Q_{{x,c_x}}^{{\psi _x}}[t,R^x\setminus R^*] + {\min \limits _{y\in \hbox {Pred}_{N}^{\downarrow }{(x)}}}\,{{\,\mathrm{\delta }\,}}(c_x,\psi _x(y)) &{} \text {if}\,\hbox {Pred}_{N}^{\downarrow }{(x)}\ne \varnothing \\ Q_{{x,c_x}}^{{\psi _x}}[t,(R^x\setminus R^*) \cup (\{x\}\cap R(N))] &{} \text {if}\, \hbox {Pred}_{N}^{\uparrow }{(x)}\ne \varnothing \end{array}\right. \end{aligned}$$

where

5 $$\begin{aligned} {Q_{{x,c_x}}^{{\psi _x}}[{i,R'}] := {\left\{ \begin{array}{ll} \min \limits _{R^*\subseteq R'\cap \hbox {Succ}_{N}^{R\uparrow }{(\Gamma _{v_i})}} Q_{{x,c_x}}^{{\psi _x}}[{i-1,R'\setminus R^*}] + T^{\mathcal {SW}}[{v_i, \psi _i, R^*}] &{} \text {if}\, i\ne 0\\ 0 &{} {if}\,i=0\hbox { and }R'=\varnothing \\ \infty &{} {otherwise} \end{array}\right. }} \end{aligned}$$

where $\psi _i:={{\psi _x}\left[ {x\rightarrow c_x}\right] }\mid _{\hbox {YW}_{{v_i}}^{\Gamma }}$ for all $i\le t$. (Note how $Q_{{x,c_x}}^{{\psi _x}}[{i,R'}]$ is used to assign the nodes in $R^x$ to the $v_i$ (with $v_0=x$) such that every node in $R^x$ has a parent in some $\Gamma _{v_i}$).

In the following, for any anti-chain *X* in $\Gamma$ and all $Z\subseteq \bigcup _{x\in X}\hbox {YW}_{x}^{\Gamma }$, let $\mathcal {S}^{X\rightarrow Z}(N)$ denote the set of all switchings *S* of *N* with $\hbox {Succ}_{S}^{R\uparrow }{(X)}=Z$.

#### Lemma 8

*Let *$\Gamma$* be a tree that agrees with* *N*,* let *$x\in V(N)$*, let *$\psi _x:\hbox {YW}_{x}^{\Gamma }\rightarrow C$* with *$\psi _x\trianglelefteq \phi$,* and let* $R^x\subseteq \hbox {Succ}_{N}^{R\uparrow }{(\Gamma _x)}$.* If* $\mathcal {S}^{\Gamma _x\rightarrow R^x}(N)=\varnothing$,* then *$T^{\mathcal {SW}}[{x,\psi _x,R^x}=\infty ]$.* Otherwise, let* $S\in \mathcal {S}^{\Gamma _x\rightarrow R^x}(N)$* and* $\psi :V(N)\rightarrow C$* such that (a)* $\psi _x\trianglelefteq \psi \trianglelefteq \phi$* and (b)* $\sum _{uw\in {A}_{x}{(S)}}{{\,\mathrm{\delta }\,}}_\psi (u,w)$* is minimum among all such* *S and* $\psi$.* Then,*

6 $$\begin{aligned} T^{\mathcal {SW}}[{x,\psi _x,R^x}]=\sum \limits _{uw\in A_x({S})}{{\,\mathrm{\delta }\,}}_\psi (u,w). \end{aligned}$$

*Proof Sketch.* Let us abbreviate $Z_i:=\bigcup _{j\le i}V(\Gamma _{v_j})$. We first show that the table *Q* does what we expect it to do.

#### Claim 2

$Q_{{x,c_x}}^{{\psi _x}}[{i,R'}] = \sum _{j\le i}\sum _{uw\in {A}_{{v_j}}{(S_i)}}{{\,\mathrm{\delta }\,}}_{\psi _i}(u,w)$ for optimal $S_i\in \mathcal {S}^{Z_i\rightarrow R'}$ and $\psi _i$ coincides with ${\psi _x}\left[ {x\rightarrow c_x}\right]$ on $\bigcup _{j\le i}\hbox {YW}_{{v_j}}^{\Gamma }$.

*Proof Sketch.* For “$$\ge $$”, let $$R^*\subseteq R'\cap \hbox {Succ}_{N}^{R\uparrow }{(\Gamma _{v_i})}$$ such that equality holds in (5). We consider a switching $$S'\in \mathcal {S}^{Z_i\rightarrow R'}$$ constructed from switchings $$S_{i-1}\in \mathcal {S}^{Z_{i-1}\rightarrow R'\setminus R^*}$$ and $$S^*\in \mathcal {S}^{\Gamma _{v_i}\rightarrow R^*}$$ as well as a mapping $$\psi '$$ coinciding with $${\psi _x}\left[ {x\rightarrow c_x}\right] $$ on $$\bigcup _{j<i}\hbox {YW}_{{v_j}}^{\Gamma }$$ constructed from mappings $$\psi _{i-1}$$ and $$\psi ^*$$ such that (a) $$\psi _{i-1}$$ coincides with $${\psi _x}\left[ {x\rightarrow c_x}\right] $$ on $$\bigcup _{j<i}\hbox {YW}_{{v_j}}^{\Gamma }$$, (b) $$\psi ^*$$ coincides with $${\psi _x}\left[ {x\rightarrow c_x}\right] $$ on $$\hbox {YW}_{{v_i}}^{\Gamma }$$, (c) the cost of $$\psi _{i-1}$$ is optimal on $$ {A}_{{Z_{i-1}}}{(S_{i-1})}$$ and (d) the cost of $$\psi ^*$$ is optimal on $$ {A}_{v_i}{(S^*)}$$. By induction hypotheses, these costs are $$Q_{{x,c_x}}^{{\psi _x}}[{i-1,R'\setminus R^*}]$$ and $$T^{\mathcal {SW}}[{{v_i,{\psi _x}\left[ {x\rightarrow c_x}\right] ,R^*}}]$$, respectively. Then, “$$\ge $$” follows by optimality of $$S_i$$ and $$\phi _i$$.

For “$$\le $$”, we let $$R^*:=\hbox {Succ}_{{S_i}}^{R\uparrow }{(\Gamma _{v_i})}$$ and use independence of sub-solutions and the induction hypotheses to show that the cost of $$\phi _i$$ on $$ {A}_{{Z_{i-1}}}{(S_i)}$$ is $$Q_{{x,c_x}}^{{\psi _x}}[{i-1,R'\setminus R^*}]$$ and the cost of $$\phi _i$$ on $$ {A}_{{v_i}}{(S_i)}$$ is $$T^{\mathcal {SW}}[{{v_i,\phi _i,R^*}}]$$. Then, “$$\le $$” follows from the fact that $$R^*$$ is only one of the possible choices for the minimum in (5).$$\square $$

For “$$\ge $$”, let $$c_x\in \phi (x)$$ and $$R^*\subseteq R^x\cap \hbox {Succ}_{N}^{R\uparrow }{(x)}$$ be such that equality holds in (4). We consider a switching $$S'\in \mathcal {S}^{\Gamma _x\rightarrow R^x}$$ constructed from switchings $$S_t$$ and $$S^*$$ with $$S_t\in \mathcal {S}^{Z_t\rightarrow R^x\setminus R^*}$$ (if $$\hbox {Pred}_{N}^{\downarrow }{(x)}\ne \varnothing $$) or $$S_t\in \mathcal {S}^{Z_t\rightarrow (R^x\setminus R^*)\cup \{x\}}$$ (if $$x\in R(N)$$ and $$\hbox {Pred}_{N}^{\uparrow }{(x)}\ne \varnothing $$), and $$S^*\in \mathcal {S}^{\{x\}\rightarrow R^*}$$, as well as a mapping $$\psi '$$ coinciding with $$\psi _x$$ on $$\hbox {YW}_{x}^{\Gamma }$$ constructed from mappings $$\psi _t$$ and $$\psi ^*$$ such that (a) $$\psi _t$$ coincides with $${\psi _x}\left[ {x\rightarrow c_x}\right] $$ on $$\bigcup _{i\le t}\hbox {YW}_{{v_i}}^{\Gamma }$$, (b) $$\psi ^*$$ coincides with $$\psi _x$$ on $$\hbox {YW}_{x}^{\Gamma }$$, (c) $$\psi ^*(x)=c_x$$, (d) the cost of $$\psi _t$$ is optimal on $$ {A}_{{Z_t}}{(S_t)}$$ and (e) the cost of $$\psi ^*$$ is optimal on $$ {A}_{\{x\}}{(S^*)}$$. Then, the cost of $$\psi ^*$$ on $$A^{\uparrow }_{\{x\}}{(S^*)}$$ is $$\sum _{r\in R^*\cup \hbox {Succ}_{N}^{T\uparrow }{(x)}}{{\,\mathrm{\delta }\,}}(c_x,\psi _x(r))$$, the cost of $$\psi ^*$$ on $$A^{\downarrow }_{\{x\}}{(S^*)}$$ is $$\min _{y\in \hbox {Pred}_{N}^{\downarrow }{(x)}}{{\,\mathrm{\delta }\,}}(c_x,\psi _x(y))$$ if the parent of *x* in $$S_t$$ is above *x* in $$\Gamma $$ (that is, $$x\notin \hbox {Succ}_{{S_t}}^{R\uparrow }{(Z_t)}$$) and, by the claim above, the cost of $$\psi _t$$ on $$ {A}_{{Z_t}}{(S_t)}$$ is $$Q_{{x,c_x}}^{{\psi _x}}[{t,\hbox {Succ}_{S_t}^{R\uparrow }{(Z_t)}}]$$. Then, as $$S'\in \mathcal {S}^{\Gamma _x\rightarrow R^x}$$, “$$\ge $$” follows by optimality of *S* and $$\phi $$.

For “$$\le $$”, let $$c_x:=\phi (x)$$ and let $$R^*:=\hbox {Succ}_{S}^{R\uparrow }{(\Gamma _x)}$$. We use independence of sub-solutions and the induction hypothesis to show that the cost of $$\phi $$ on $$ {A}_{{Z_t}}{(S)}$$ is $$Q_{{x,c_x}}^{{\psi _x}}[{t,R'\setminus R^*}]$$ (if $$x\notin R(N)$$ or the parent of *x* in *S* is above *x* in $$\Gamma $$) or $$Q_{{x,c_x}}^{{\psi _x}}[{t,(R'\setminus R^*)\cup \{x\}}]$$ (if $$x\in R(N)$$ and the parent of *x* in *S* is in $$\Gamma _x$$). Further, the cost of $$\psi $$ on $$A^{\uparrow }_{\{x\}}{(S)}$$ is $$\sum _{r\in R^*\cup \hbox {Succ}_{N}^{T\uparrow }{(x)}}{{\,\mathrm{\delta }\,}}(c_x,\psi _x(r))$$, the cost of $$\psi $$ on $$A^{\downarrow }_{\{x\}}{(S)}$$ is $$\min _{y\in \hbox {Pred}_{N}^{\downarrow }{(x)}}{{\,\mathrm{\delta }\,}}(c_x,\psi _x(y))$$ if the parent of *x* in *S* is above *x* in $$\Gamma $$. Then, “$$\le $$” follows from the fact that our choices of $$c_x$$ and $$R^*$$ are only one of the possible choices for the minimum in (4).

In order to solve the softwired parsimony problem given *N*, $\phi$ and $\Gamma$, all we have to do is compute $T^{\mathcal {SW}}[{{x,\psi _x,R^x}}]$ for each *x* bottom-up in $\Gamma$, each of the (at most) $|C|^{|\hbox {YW}_{x}^{\Gamma }|}$ many choices of $\psi _x:\hbox {YW}_{x}^{\Gamma }\rightarrow C$ with $\psi _x\trianglelefteq \phi$, and each $R^x\subseteq \hbox {Succ}_{N}^{R\uparrow }{(x)}\subseteq \hbox {YW}_{x}^{\Gamma }\cap R(N)$. To this end, $Q_{{x,c_x}}^{{\psi _x}}[{i,R^x\setminus R^*}]$ and $Q_{{x,c_x}}^{{\psi _x}}[{i,(R^x\setminus R^*)\cup \{x\}}]$ have to be computed for each child $v_i$ of *x* in $\Gamma$ and each $R^*\subseteq R^x\cap \hbox {Succ}_{N}^{R\uparrow }{(x)}$. Then, by Lemma 8, the softwired parsimony score of *N* with respect to $\phi$ can be read from $T^{\mathcal {SW}}[{{\rho _\Gamma ,\varnothing ,\varnothing }}]$. In the following, let $\psi _x$ be fix. Then, for fix $c_x$, we can compute $Q_{{x,c_x}}^{{\psi _x}}[{i,R'}]$ for all choices of *x*, *i* and $R'$ in $O(2^{|R'\cap \hbox {Succ}_{N}^{R\uparrow }{(v_i)}|}+\sum _{x\in \Gamma }|\hbox {Succ}_{\Gamma }{(x)}|)\subseteq O(2^{|\hbox {YW}_{x}^{\Gamma }|+1}+|\Gamma |)$ time total. Further, the values of $\min _{y\in \hbox {Pred}_{N}^{\downarrow }{(x)}}{{\,\mathrm{\delta }\,}}(c_x,\phi _x(y))$ can be pre-computed for all $x\in \Gamma$ in *O*(|*A*(*N*)|) time total. Then, to compute $T^{\mathcal {SW}}[{{x,\psi _x,R^x}}]$ for all *x* and $R^x$, we have to check |*V*(*N*)| choices for *x*, as well as $|\phi (x)|\le |C|$ choices for $c_x$ and $3^{|\hbox {Succ}_{N}^{R\uparrow }{(x)}|}$ choices for $R^x$ and $R^*\subseteq R^x$ combined. Altogether, the table $T^{\mathcal {SW}}$ can be computed in $O(|C|^{|\hbox {YW}_{x}^{\Gamma }|}\cdot (3^{|\hbox {YW}_{x}^{\Gamma }|}\cdot |C|\cdot |V(N)| + |A(N)|))$ time. The computation of $Q_{{x,c_x}}^{{\psi _x}}$ in $O(2^{|\hbox {YW}_{x}^{\Gamma }|} + |A(N)|)$ time is absorbed by this. For practical purposes, note that estimating $|\hbox {Succ}_{N}^{R\uparrow }{(x)}|\le |\hbox {YW}_{x}^{\Gamma }|$ is quite crude and equality will almost never be attained. Then, the following result holds:

#### Theorem 2

*Given a network N*, $\phi :V(N)\rightarrow 2^{C}$* and a tree* $\Gamma$* agreeing with* *N*,* the softwired parsimony score of* $(N,\phi )$* can be computed in *$O(|C|^{\hbox {yw}(\Gamma )}\cdot (3^{\hbox {yw}(\Gamma )}\cdot |C|\cdot |V(N)|+|A(N)|))$ *time.*

Again, we can replace $\hbox {yw}(\Gamma )$ by $\hbox {tw}{(N)}$ using Proposition 1.

#### Corollary 2

*Let* $(N,\phi )$* be an instance of* Softwired Parsimony.* Let *$t\ge \hbox {tw}{(N)}$* and let* *T be the time in which a width-t tree decomposition of* *N can be computed. Then, the softwired parsimony score of* $(N,\phi )$* can be computed in *$O(T + |C|^t\cdot (3^t\cdot |C|\cdot |V(N)|+|A(N)|))$ *time.*

### Parental parsimony

For ease of presentation, we introduce some additional notation. First, for any *a* and *b*, we abbreviate $\max \{a-b,0\}=:a{\mathop {-}\limits ^{.}}b$. Let $\psi$ and $\psi '$ be functions. If $\psi$ maps all items to $\varnothing$ or to 0, then we say that $\psi$ is a *zero-function* and we write $\psi =\overrightarrow{0}$. We use $\psi -\psi '$ to denote the function defined on the domain of $\psi$ for which $(\psi -\psi ')(x) = \psi (x)$ if $\psi '(x)=\bot$ and $(\psi -\psi ')(x)=\psi (x)-\psi '(x)$, otherwise. This definition extends to functions mapping to sets in a natural way.

Each finite-cost lineage function *f* corresponds to a phylogenetic tree “embedded” in *N* whose branches are called *lineages* (see Fig. 1(right)). For each $x\in V(N)$, *f*(*x*) represents the set of such lineages passing through *x*. Each such lineage may “choose” a parent among the parents of *x* in *N*. This models the biological circumstance that a character trait may be inherited from any parent. We compute (the cost of) an optimal lineage function on *N* using a tree $\Gamma$ that agrees with *N*. To compute $\hbox {cost}_{f}{(x)}$, we require knowledge of $\sum _{y\in \hbox {Pred}{(x)}}|f(y)|$ as well as $\bigcup _{y\in \hbox {Pred}{(x)}}f(y)$ (see Definition 4). We partition the predecessors of *x* over which the formula iterates into those above *x* in $\Gamma$ and those below (since $\Gamma$ agrees with *N*, all predecessors of *x* in *N* are comparable to *y* in $\Gamma$). For all $y\in \hbox {YW}_{x}^{\Gamma }$, we thus store

1. the set $\lambda (y):=f(y)$ of lineages in *y*,
2. the subset $\psi (y)$ of lineages of *y* that also occur in parents (in *N*) of *y* that are below *x* in $\Gamma$, that is, in $\hbox {Pred}_{N}^{\uparrow x}{(y)}$ (such lineages are inherited by *y* at no cost),
3. the total number $\eta (y)$ of lineages of *y* that can be inherited from parents (in *N*) of *y* that are below *x* in $\Gamma$, that is, from $\hbox {Pred}_{N}^{\uparrow x}{(y)}$ (cost 0 or 1).

Then, in order to compute an entry $T^{\mathcal {PT}}[{{x,\lambda _x,\psi _x,\eta _x}}]$, we “guess” the set $U\subseteq \phi (x)$ of lineages passing through *x* in an optimal solution, as well as the set $D\subseteq U$ of lineages inherited from nodes in $\hbox {Pred}_{N}^{\uparrow }{(x)}$. This allows us to infer $\eta (x)=|\lambda (x)| {\mathop {-}\limits ^{.}}\sum _{r\in \hbox {Pred}_{N}^{\downarrow }{(x)}}|\lambda (r)|$ and $\psi (x):=D$. Then, by Definition 4, the cost incurred by *f* on *x* can be computed from $\sum _{y\in \hbox {Pred}_{N}{(x)}}|f(y)|= \eta (x)+\sum _{y\in \hbox {Pred}_{N}^{\downarrow }{(x)}}|\lambda (y)|$ and $\bigcup _{y\in \hbox {Pred}_{N}{(x)}}f(y)= \psi (x)\cup \bigcup _{y\in \hbox {Pred}_{N}^{\downarrow }{(x)}}\lambda (y)$.

We will compute table entries for *x* using the already computed table entries for the children $v_i$ of *x* in $\Gamma$. In these lookups, we have $x\in \hbox {YW}_{{v_i}}^{\Gamma }$ so, to be consistent with the semantics, we have to make sure that $\lambda (x)=U$, $\psi (x)=D$, and that all lineages of *x* that are not inherited from $\hbox {Pred}_{N}^{\downarrow }{(x)}$ can be inherited from $\hbox {Pred}_{N}^{\uparrow }{(x)}$, that is, $\eta (x)=|\lambda (x)| {\mathop {-}\limits ^{.}}\sum _{r\in \hbox {Pred}_{N}^{\downarrow }{(x)}}|\lambda (r)|$. Further, each child *y* of *x* in *N* may inherit a lineage from *x* and, if *y* is above *x* in $\Gamma$, this has to be registered by removing the lineages of *U* from $\psi (y)$ and subtracting |*U*| from $\eta (y)$. Finally, the lineages represented by $\psi$ and $\eta$ are distributed among the children of *x* in $\Gamma$ using the table *Q*. In the following, in order to avoid treating the case that $x=\rho _N$ separately, we define $\rho (x):=1-{{\,\mathrm{\delta }\,}}(x,\rho _N)$, that is, $\rho (x)=1$ if and only if $x=\rho _N$.

#### Definition 7

Let $\Gamma$ be a tree that agrees with *N*, let $x\in V(N)$, let $\lambda _x:\hbox {YW}_{x}^{\Gamma }\rightarrow 2^{C}$ with $\lambda _x\trianglelefteq \phi$ and let $\psi _x\trianglelefteq {\lambda _x}$. Let $\{v_1,v_2,\ldots ,v_t\}=\hbox {Succ}_{\Gamma }{(x)}$ ($t=0$ if *x* is a leaf in $\Gamma$). Then, set $T^{\mathcal {PT}}[{{x,\lambda _x,\psi _x,\eta _x}}]$ to

7 $$\begin{aligned} \min _{\begin{array}{c} D \subseteq U\subseteq \phi (x)\\ U\ne \varnothing \end{array}} Q_{x}^{{\lambda _x}{x\rightarrow U}} \left[ t, {\psi _x}\left[ {\begin{array}{ll} {x\rightarrow D}&{}\\ {\forall }_{y\in \hbox {Succ}_{N}^{\uparrow }{(x)}}&{} y\rightarrow \psi _x(y)\setminus U \end{array}}\right] , {\eta _x}\left[ {\begin{array}{l} x\rightarrow |U| {\mathop {-}\limits ^{.}}\sum _{u\in \hbox {Pred}_{N}^{\downarrow }{(x)}}|\lambda _x(u)|\\ {\forall }_{y\in \hbox {Succ}_{N}^{\uparrow }{(x)}}y\rightarrow \eta _x(y){\mathop {-}\limits ^{.}}|U|\end{array}}\right] \right] \nonumber \\ + \left| U\setminus \left( D \cup {\bigcup _{u\in \hbox {Pred}_{N}^{\downarrow }{(x)}}}\lambda _x(u)\right) \right| \end{aligned}$$

where $$Q_{x}^{\lambda }[i,\psi ,\eta ]$$ equals

8 $$\begin{aligned} \left\{ \begin{array}{ll} \min \limits _{\psi '\trianglelefteq {\psi }\mid _{\hbox {YW}_{{v_i}}^{\Gamma }}} \min \limits _{\eta '\trianglelefteq {\eta }\mid _{\hbox {YW}_{{v_i}}^{\Gamma }}} Q_{x}^{\lambda }[{i-1,\psi -\psi ',\eta -\eta '}] + T^{\mathcal {PT}} [{v_i,{\lambda }\mid _{\hbox {YW}_{{v_i}}^{\Gamma }},\psi ',\eta '}] &{} \\ {} &{} {if}\,i>0\\ -\rho (x) &{} {if}\,i=0\hbox { and }\psi =\overrightarrow{0}\hbox { and }\eta ={\overrightarrow{0}}\left[ {x\rightarrow \rho (x)}\right] \\ \infty &{} {otherwise} \end{array}\right. \end{aligned}$$

Note how the table $Q_{x}^{\lambda }$ distributes the lineage branches of *x* whose parents are in $\Gamma _x$ among the children of *x* in $\Gamma$. We show that both $T^{\mathcal {PT}}$ and $Q_{x}^{\lambda }$ are monotone in $\psi$ and $\eta$ (wrt. $\trianglelefteq$).

#### Lemma 9

*Let* $x\in V(N)$,* let* $i\in \mathbb {N}$,* let *$\lambda :\hbox {YW}_{x}^{\Gamma }\rightarrow 2^{C}$,* let *$\eta ,\eta ':\hbox {YW}_{x}^{\Gamma }\rightarrow \mathbb {N}$,* and let *$\psi ,\psi ':\hbox {YW}_{x}^{\Gamma }\rightarrow 2^{C}$* such that *$\psi '\trianglelefteq \psi \trianglelefteq \lambda$* and *${\overrightarrow{0}}\left[ {x\rightarrow \rho (x)}\right] \trianglelefteq \eta '\trianglelefteq \eta$.* Then,*

$$\begin{aligned} T^{\mathcal {PT}}[{x,\lambda ,\psi ',\eta '}] \le T^{\mathcal {PT}}[{x,\lambda ,\psi ,\eta }]&\text {and}&Q_{x}^{\lambda }[{i,\psi ',\eta '}] \le Q_{x}^{\lambda }[{i,\psi ,\eta }] \end{aligned}$$

*Proof Sketch.* The lemma can be proved by induction on the height of *x* in $\Gamma$ and the value of *i*. If *x* is a leaf, then $Q_{x}^{\lambda }[{0,\psi ,\eta }]$ is finite only if $\psi =\overrightarrow{0}$ and $\eta ={\overrightarrow{0}}\left[ {x\rightarrow \rho (x)}\right]$, implying the second inequality. For monotony of $T^{\mathcal {PT}}$, fix the sets $D\subseteq U\subseteq \phi (x)$ for which the minimum in the formula of ${T^{\mathcal {PT}}[{x,\lambda ,\psi ,\eta }]}$ is attained. Then, by monotony of $Q_{x}^{\lambda }$, replacing $\psi$ by $\psi '$ and $\eta$ by $\eta '$ in this formula does not increase its value and this value is at most ${T^{\mathcal {PT}}[{x,\lambda ,\psi ',\eta '}]}$ since it is obtained for one of several possible choices for *D* and *U*. If *x* is not a leaf in $\Gamma$ then monotonicity of $Q_{x}^{\lambda }[{i,\ldots }]$ is implied by monotonicity of $Q_{x}^{\lambda }[{i-1,\ldots }]$ and monotonicity of ${T^{\mathcal {PT}}[{v,\ldots }]}$ for the children *v* of *x*. Finally, monotonicity of $T^{\mathcal {PT}}$ follows from monotonicity of $Q_{x}^{\lambda }$ as in the induction base.$\square$

#### Lemma 10

*Let* $\Gamma$* be a tree agreeing with* *N*,* let* $x\in V(N)$,* let *$\psi _x,\lambda _x:\hbox {YW}_{x}^{\Gamma }\rightarrow 2^{c}$* and *$\eta _x:\hbox {YW}_{x}^{\Gamma }\rightarrow \mathbb {N}$.* Let* *f minimize *$\hbox {cost}_{(f)}$* among all lineage functions in* $\mathcal {LF}_{N,\phi }$* such that, for all* $w\in \hbox {YW}_{x}^{\Gamma }$, $\lambda _x(w) = f(w)$, $\psi _x(w) = f(w)\cap \bigcup _{u\in \hbox {Pred}_{N}^{\uparrow x}{(w)}}f(u)$,* and* $\eta _x(w) \le \sum _{u\in \hbox {Pred}_{N}{\uparrow x}{(w)}}|f(u)|$. *If there are no such* *f*,* then* ${T^{\mathcal {PT}}[{x,\lambda _x,\psi _x,\eta _x}=\infty ]}$.* Otherwise,*

$$\begin{aligned} {T^{\mathcal {PT}}[{x,\lambda _x, \psi _x, \eta _x}] = \sum _{z\le _\Gamma x}\hbox {cost}_{f}{(z)}} \end{aligned}$$

*Proof Sketch.* Let us abbreviate $Z_i:=\bigcup _{j\le i}V(\Gamma _{v_j})$. We first show that the table *Q* does what we expect it to do.

#### Claim 3

Let $\lambda ,\psi :\hbox {YW}_{x}^{\Gamma }\cup \{x\}\rightarrow 2^{C}$ and $\eta :\hbox {YW}_{x}^{\Gamma }\cup \{x\}\rightarrow \mathbb {N}$ such that $\psi \trianglelefteq \lambda \trianglelefteq \phi$. Let $f_i\in \mathcal {LF}_{N,\phi }$ have minimum cost on $\bigcup _{j\le i}\Gamma _{v_j}$ among all lineage functions for *N* that, for all $w\in \bigcup _{j\le i}\hbox {YW}_{{v_j}}^{\Gamma }$, satisfy (a) $\lambda (w) = f_i(w)$, (b) $\psi (w) = f_i(w)\cap \bigcup _{j\le i}\bigcup _{u\in \hbox {Pred}_{N}^{\uparrow v_j}{(w)}}f_i(u)$, and (c) $\eta (w) \le \sum _{j\le i}\sum _{u\in \hbox {Pred}_{N}^{\uparrow v_j}{(w)}}|f_i(u)|$ Then, $Q_{x}^{\lambda }[{i,\psi ,\eta }] = \sum _{j\le i}\sum _{u\in \Gamma _{v_j}}\hbox {cost}_{{f_i}}{(u)}$.

*Proof Sketch.* For “$\ge$”, let $\psi '\trianglelefteq {\psi }\mid _{\hbox {YW}_{{v_i}}^{\Gamma }}$ and $\eta '\trianglelefteq {\eta }\mid _{\hbox {YW}_{{v_i}}^{\Gamma }}$ such that equality holds in (8). Let $f_{i-1}\in \mathcal {LF}_{N,\phi }$ minimize $\sum _{j<i}\sum _{u\in \Gamma _{v_j}}\hbox {cost}_{{f_{i-1}}}{(u)}$ among all lineage functions satisfying (a)–(c) for $i-1$. Let $f^*\in \mathcal {LF}_{N,\phi }$ minimize $\sum _{u\in \Gamma _{v_i}}\hbox {cost}_{f^*}{(u)}$ among all lineage functions that, for all $w\in \hbox {YW}_{{v_i}}^{\Gamma }$, satisfy $\lambda (w)=f^*(w)$, $\psi '(w)=f^*(w)\cap \bigcup _{u\in \hbox {Pred}_{N}^{\uparrow v_i}{(w)}}f^*(u)$ and $\eta '(w)=\sum _{u\in \Gamma _{v_i}}|f^*(u)|$. By induction hypotheses, the cost of $f_{i-1}$ on $Z_i$ is $Q_{x}^{\lambda }[{i-1,\psi -\psi ',\eta -\eta '}]$ and the cost of $f^*$ on $\Gamma _{v_i}$ is ${T^{\mathcal {PT}}[{v_i,{\lambda }\mid _{\hbox {YW}_{{v_i}}^{\Gamma }},\psi ',\eta '}]}$. From $f_{i-1}$ and $f^*$, we construct a lineage function $f'\in \mathcal {LF}_{N,\phi }$ whose cost on $Z_i$ is $\sum _{j<i}\sum _{u\in \Gamma _{v_j}}\hbox {cost}_{{f_{i-1}}}{(u)} + \sum _{u\in \Gamma _{v_i}}\hbox {cost}_{f^*}{(u)}$. Then, “$\ge$” follows by optimality of $f_i$ on $Z_i$.

For “$\le$”, let $\psi '$ and $\eta '$ be such that, for all $w\in \hbox {YW}_{{v_i}}^{\Gamma }$, we have $\psi '(w)=f_i(w)\cap \bigcup _{u\in \hbox {Pred}_{N}^{\uparrow v_i}{(w)}}f_i(u) \subseteq \psi (w)$ and $\eta '(w)=\sum _{u\in \hbox {Pred}_{N}^{\uparrow v_i}{(w)}}|f_i(u)|$. By independence of sub-solutions, $f_i$ is optimal on $Z_{i-1}$ and on $\Gamma _{v_i}$ so, by induction hypotheses, the cost of $f_i$ on $Z_{i-1}$ is $Q_{x}^{\lambda }[{i-1,\psi -\psi ',\eta -\eta '}]$ and the cost of $f_i$ on $\Gamma _{v_i}$ is ${T^{\mathcal {PT}}[{v_i,{\lambda }\mid _{\hbox {YW}_{{v_i}}^{\Gamma }},\phi ',\eta '}]}$. Since $\psi '$ and $\eta '$ are only one of the possible choices for the minimum in (8), “$\le$” follows.$\square$

For “$\ge$”, let $D\subseteq U\subseteq \phi (x)$ such that equality holds in (7). We construct a lineage function $f'$ that assigns $f'(x)=U$ and such that the lineages of *D* are inherited from parents of *x* (in *N*) that are below *x* in $\Gamma$. To this end, we ask the dynamic programming table for the cost of a lineage function that is optimal on $Z_t$ and such that 1. $\psi '(x)=D$ *(lineages in* *D* *are inherited from parents of* *x* *in* $\Gamma _x$) 2. $\psi '(w)=\psi '(w)\setminus U$ for all $w\in \hbox {Succ}_{N}^{\uparrow }{(x)}$ *(children of* *x* *in* $\hbox {YW}_{x}^{\Gamma }$ *no longer need to inherit the lineages in* *U* *from* $\Gamma _x$) 3. $\eta '(x)=|U|{\mathop {-}\limits ^{.}}\sum _{u\in \hbox {Pred}_{N}^{\downarrow }{(x)}}|\lambda _x(u)|$ (*x* *needs to inherit* |*U*| *lineages in total:* $|\lambda _x(u)|$ *come from every parent* *u* *of* *x* *in* $\hbox {YW}_{x}^{\Gamma }$ *while the rest has to be inherited from* $\Gamma _x$) and 4. $\eta '(w)=\eta _x(w){\mathop {-}\limits ^{.}}|U|$ for all $w\in \hbox {Succ}_{N}^{\uparrow }{(x)}$ *(children of* *x* *in* $\hbox {YW}_{x}^{\Gamma }$ *can inherit a maximum of* |*U*| *lineages from* *x*). Since the functions $\lambda ':={\lambda _x}\left[ {x\rightarrow U}\right]$, $\psi ':={\psi _x}\left[ {x\rightarrow D, \forall _{u\in \hbox {Succ}_{N}^{\uparrow }{(x)}}w\rightarrow \psi _x(w)\setminus U}\right]$ and $\eta ':={\eta _x}\left[ {x\rightarrow |U|{\mathop {-}\limits ^{.}}\sum _{u\in \hbox {Pred}_{N}^{\downarrow }{(x)}}|\lambda _x(u)|, \forall _{u\in \hbox {Succ}_{N}^{\uparrow }{(x)}}w\rightarrow \eta _x(w){\mathop {-}\limits ^{.}}|U|}\right]$ satisfy the conditions of Claim 3, the optimal cost of such a lineage function $f'$ on $Z_t$ is $Q_{x}^{\lambda }[{t,\psi ',\eta '}]$. Further, the cost of $f'$ on *x* is the number of lineages in *U* that is not inherited “for free” from parents of *x*, that is, $|U\setminus (D\cup \bigcup _{u\in \hbox {Pred}_{N}^{\downarrow }{(x)}}\lambda _x(u))|$. Then, “$\ge$” follows by optimality of *f* on $\Gamma _x$.

For “$\le$”, let $U:=f(x)$ and let $D:=U\cap \bigcup _{u\in \hbox {Pred}_{N}^{\uparrow }{(x)}}f(x)$ be the set of lineages of *U* that are inherited from parents of *x* in *N* that are below *x* in $\Gamma$. By independence of sub-solutions, *f* is optimal on $Z_t$ so, by Claim 3, its cost on $Z_t$ is $Q_{x}^{\lambda }[{t,\psi ',\eta '}]$ where $\psi ':={\psi _x}\left[ {\ldots }\right]$ and $\eta ':={\eta _x}\left[ {\ldots }\right]$ are defined as in (7) and its cost on *x* is $|f(x)\setminus (\bigcup _{u\in \hbox {Pred}_{N}^{\uparrow }{(x)}}f(x)\cup \bigcup _{u\in \hbox {Pred}_{N}^{\downarrow }{(x)}}f(x))|=|U\setminus (D\cup \bigcup _{\hbox {Pred}_{N}^{\downarrow }{(x)}}f(x))|$. Then, “$\le$” follows from the fact that *U* and *D* are only one of the possible choices for the minimum in (7).$\square$

To solve the parental parsimony problem given *N*, $\phi$ and $\Gamma$, we compute ${T^{\mathcal {PT}}[{x,\lambda _x,\psi _x,\eta _x}]}$ for each *x* bottom-up in $\Gamma$, each $\psi _x,\lambda _x:\hbox {YW}_{x}^{\Gamma }\rightarrow 2^{C}$ with $\psi _x\trianglelefteq \lambda _x\trianglelefteq \phi$ and each $\eta _x:\hbox {YW}_{x}^{\Gamma }\rightarrow \{0,\ldots ,|C|\}$ (by Definition 7, no value larger than |*C*| ever enters $\eta _x$ and all modifications to $\eta _x$ decrease the mapped-to values). To this end, $Q_{x}^{\lambda }[{i,\psi ,\eta }]$ is computed for each *x*, *i*, $\lambda$, $\psi$, and $\eta$ by making at most $2^{|C|\cdot |\hbox {YW}_{x}^{\Gamma }|}\cdot |C|^{|\hbox {YW}_{x}^{\Gamma }|}$ queries to $Q_{{x,c_x}}^{{\psi _x}}$ and $T^{\mathcal {PT}}$. As there are *O*(|*A*(*N*)|) valid combinations of *x* and *i*, the table *Q* can be computed in $O(|A(N)| \cdot 3^{|C|\cdot \hbox {yw}{(N)}} \cdot |C|^{\mathrm{yw}{(N)}} \cdot 2^{|C|\cdot \hbox {yw}{\mathrm{N}}} \cdot |C|^{\mathrm{yw}{(N)}} ) =O(|A(N)| \cdot 6^{|C|\cdot \hbox {yw}{(N)}|} \cdot 4^{\mathrm{yw}{(N)}\cdot \log {|C|}} )$ time. Further, computing each ${T^{\mathcal {PT}}[{x,\lambda _x,\psi _x,\eta _x}]}$ requires testing $3^{|\phi (x)|}\le 3^{|C|}$ choices for $D\subseteq U\subseteq \phi (x)$ and computing $|U\setminus (D\cup \bigcup _{u\in \hbox {Pred}_{N}^{\downarrow }{(x)}}\lambda _x(u))|$ in *O*(|*C*|) time (we precompute $\bigcup _{u\in \hbox {Pred}_{N}^{\downarrow }{(x)}}\lambda _x(u)$ for each fix *x* and $\lambda _x$). Thus, the table $T^{\mathcal {PT}}$ can be computed in $O(3^{|C|\cdot \hbox {yw}{(N)}}\cdot (|C|^{\mathrm{yw}{(N)}+1}\cdot 3^{|C|} + |A(N)|))$ time, which is dominated by the construction of *Q*.

#### Theorem 3

*Given a network* *N*, $\phi :V(N)\rightarrow 2^{C}$* and a tree* $\Gamma$* agreeing with* *N*,* the parental parsimony score of* $(N,\phi )$* can be computed in* $O(6^{\hbox {yw}(\Gamma )\cdot |C|}\cdot 4^{\hbox {yw}(\Gamma )\cdot \log |C|}\cdot |A(N)|)$* time*.

Again, we can replace $\hbox {yw}(\Gamma )$ by $\hbox {tw}{(N)}$ using Proposition 1.

#### Corollary 3

*Let* $(N,\phi )$* be an instance of* Parental Parsimony.* Let *$t\ge \hbox {tw}{(N)}$* and let T be the time in which a width-t tree decomposition of* *N* can be computed. Then, the parental parsimony score of $(N,\phi )$* can be computed in* $O(T+ 6^{t\cdot |C|}\cdot 4^{t\cdot \log |C|}\cdot |A(N)|)$* time*.

Note that the parental parsimony setting supports assigning multiple states of a character to a single species, thereby modeling species carrying multiple alleles of a single gene. By forcing $D\subseteq U = \phi (x)$ instead of $D\subseteq U\subseteq \phi (x)$ if *x* is a leaf, we can trivially modify our dynamic programming to explain multiple character states in extant species.

Corollaries 1, 2 and 3 give the running times of our algorithms as depending on the treewidth of *N*. The state-of-the-art solutions for Hardwired Parsimony, Softwired Parsimony and Parental Parsimony have the following respective running times: $O(|C|^{r+2}|V(N)|)$ [9], $O(2^\ell |C|^2|V (N)||A(N)|)$ [8] and $O(|2^C|^{\ell +3}|V(N)|)$ [12]. Since the scanwidth of *N* is potentially much smaller than its level $\ell$ [28], and the treewidth of *N* is smaller than its scanwidth [20], we have $\hbox {tw}{(N)}-1\le \ell \le r$. Thus, we expect that there will be several cases where our algorithms will be faster than the current best-known ones.

## Discussion

In this paper, we focused on the small version of the parsimony problem for networks given a specific position in the genome. When markers can be assumed to be independent, as it is the case when a certain distance is preserved between genomic locations included in the matrix, each position can be analyzed separately, and the parsimony score of a network w.r.t. the matrix is simply the sum of the parsimony scores of the network for each genomic location. Thus, the algorithms presented here can be easily expanded to several independent genomic locations. Moreover, our formulations are defined for networks that are not necessarily binary, can account for polymorphism and can impose restrictions on ancestral states. As discussed above, our algorithms can be orders of magnitude faster than the state-of-the-art solutions. A comparison of the reticulation number, the level, the scanwidth and the treewidth for practically relevant classes of networks would thus be an interesting project for future work.

Our results are slightly overshadowed by the fact that optimal tree decompositions are very hard to compute. However, practical exact and approximative algorithms are available today and we expect them do perform well, as phylogenetic networks can be expected to only be moderately tangled.

Furthermore, closer inspection of our dynamic programming formulations (most prominently Definition 6) unveils that their computation is faster when the maximum number of reticulations in each bag is small. Thus, it would be interesting to be able to compute tree decompositions in which this quantity is low, to the point where one could improve running time of the algorithm by sacrificing optimality of the decomposition in favor of reducing this “reticulation density”. Research in this direction is, to the best of our knowledge, limited to a paper by Bachoore and Bodlaender [29], considering tree decompositions minimizing a *weight function* over the bags.

The ability to fast-score phylogenetic networks under the parsimony framework could be a big help in designing likelihood-based heuristics or bayesian methods to infer networks from independent markers [28, 30] by providing fast heuristics to compute the initial networks with which to start the likelihood or bayesian search, or to design fast local-search techniques.

In the future, we would like to tackle the small parsimony problem for several *dependent* genomic locations (e.g. a gene). Little is known for this problem, except that it stays NP-hard even for binary characters on level-1 networks [31] and that it is fixed-parameter tractable in the number of reticulations of the network [6]. Another important direction would be to study the big parsimony problem, which is currently wide open, even lacking a consensus of the definition of optimality [6, 32–34].

## Appendix

### Lemma 7

*Let* $x\in V(N)$* and let* $\psi _x:\hbox {YW}_{x}^{\Gamma }\rightarrow C$* with* $\psi _x\trianglelefteq \phi$.* Let *$\psi :V(N)\rightarrow C$* with* $\psi _x\trianglelefteq \psi \trianglelefteq \phi$* such that* $\psi$* minimizes* $\sum _{uw\in {A}_{x}{(N)}}{{\,\mathrm{\delta }\,}}_\psi (u,w)$.* Then,*

$$\begin{aligned} T^{\mathcal {HW}}[{x,\psi _x}] = \sum \limits _{uw\in A_x({N})}{{\,\mathrm{\delta }\,}}_\psi (u,w) \end{aligned}$$

### Proof

The proof is by induction on the height of *x* in $\Gamma$. For the induction base, suppose that *x* is a leaf in $\Gamma$ and note that ${A}_{x}{(N)}= {A}_{\{x\}}{(N)}$ in this case. Then, (3) simplifies to

9 $$\begin{aligned} T^{\mathcal {HW}}[{x,\psi _x}]&= \min _{c_x\in \phi (x)} \sum \limits _{z\in \hbox {Pred}_{N}^{\downarrow }{(x)}\cup \hbox {Succ}_{N}^{\uparrow }{(x)}} {{\,\mathrm{\delta }\,}}(c_x,\psi _x(z))\nonumber \\&= \min _{c_x\in \phi (x)} \left( \sum \limits _{zx\in A^{\downarrow }_{x}{(N)}}{{\,\mathrm{\delta }\,}}(c_x,\psi _x(z)) + \sum \limits _{xz\in A^{\uparrow }_{x}{(N)}}{{\,\mathrm{\delta }\,}}(c_x,\psi _x(z)) \right) \nonumber \\&= \min _{c_x\in \phi (x)} \sum \limits _{uw\in A_x ({N})}{{\,\mathrm{\delta }\,}}_{{\psi _x}\left[ {x\rightarrow c_x}\right] }(u,w) \end{aligned}$$

Since $$\psi (x)\in \phi (x)$$, we know that $$\psi (x)$$ participates in the minimum in (9), implying the “$$\le $$”-direction. For the “$$\ge $$”-direction, assume that $$T^{\mathcal {HW}}[{x,\psi _x}]<\sum _{uw\in {A}_{x}{(N)}}{{\,\mathrm{\delta }\,}}_\psi (u,w)$$. By (9), there is some $$c_x\ne \psi (x)$$ with $$c_x\in \phi (x)$$ and $$\sum _{uw\in {A}_{x}{(N)}}{{\,\mathrm{\delta }\,}}_{{\psi _x}\left[ {x\rightarrow c_x}\right] }(u,w) < \sum _{uw\in {A}_{x}{(N)}}{{\,\mathrm{\delta }\,}}_\psi (u,w)$$. Since $$c_x\in \phi (x)$$, we still have $$\psi _x\trianglelefteq {\psi _x}\left[ {x\rightarrow c_x}\right] \trianglelefteq \phi $$, contradicting optimality of $$\psi $$ on $$ {A}_{x}{(N)}$$. For the induction step, suppose that $$t>0$$ and consider both directions separately. “$$\le $$”: Let $$i\le t$$, and let $$\psi _i:={\psi }\mid _{\hbox {YW}_{{v_i}}^{\Gamma }}={{\psi _x}\left[ {x\rightarrow \psi (x)}\right] }\mid _{\hbox {YW}_{{v_i}}^{\Gamma }}$$. Then, by Lemma 6 (with $$Z=\{x\}$$, $$Y=\{v_i\}$$, $$\mathcal {G}=\{N\}$$ and $$(S^y)_{y\in Y}=(N)_{y\in Y}$$), optimality of $$\psi $$ on $$ {A}_{x}{(N)}$$ implies optimality of $$\psi _i$$ on $$ {A}_{{v_{i}}}{(N)}$$. Thus, we can use the induction hypothesis on $$T^{\mathcal {HW}}[{v_i,\psi _i}]$$. Since $$\psi (x)$$ participates in the minimum of (3),

$$\begin{aligned} T^{\mathcal {HW}}[{x,\psi _x}]&{\mathop {\le }\limits ^{(3)}} \sum \limits _{1\le i\le t} T^{\mathcal {HW}}[{v_i,\psi _i}] + \sum \limits _{z\in \hbox {Pred}_{N}^{\downarrow }{(x)}\cup \hbox {Succ}_{N}^{\uparrow }{(x)}} {{\,\mathrm{\delta }\,}}(\psi (x),\psi _x(z))\\&{\mathop {=}\limits ^{\text {IH}}} \sum _{1\le i\le t}\; \sum \limits _{uw\in {A}_{{v_{i}}}{(N)}}{{\,\mathrm{\delta }\,}}_\psi (u,w) + \sum \limits _{z\in \hbox {Pred}_{N}^{\downarrow }{(x)}\cup \hbox {Succ}_{N}^{\uparrow }{(x)}} {{\,\mathrm{\delta }\,}}_\psi (x,z)\\&= \sum \limits _{uw\in A_x ({N})}{{\,\mathrm{\delta }\,}}_\psi (u,w) \end{aligned}$$

“$\ge$”: Assume towards a contradiction that the lemma is false, that is, “<” holds. By (3), there is some $c_x\in \phi (x)$ such that

10 $$\begin{aligned} T^{\mathcal {HW}}[{x,\psi _x}] = \sum \limits _{1\le i\le t} T^{\mathcal {HW}}[{v_i,{{\psi _x}\left[ {x\rightarrow c_x}\right] }\mid _{\hbox {YW}_{{v_i}}^{\Gamma }}}] + \sum \limits _{z\in \hbox {Pred}_{N}^{\downarrow }{(x)}\cup \hbox {Succ}_{N}^{\uparrow }{(x)}} {{\,\mathrm{\delta }\,}}(c_x,\psi _x(z)) \end{aligned}$$

Since $c_x\in \phi (x)$, we can extend ${\psi _x}\left[ {x\rightarrow c_x}\right]$ to *V*(*N*) without violating $\phi$, that is, there are functions $\psi ':V(N)\rightarrow C$ with ${\psi _x}\left[ {x\rightarrow c_x}\right] \trianglelefteq \psi '\trianglelefteq \phi$. Among them, let $\psi '$ minimize $\sum _{i\le t}\sum _{uw\in {A}_{{v_{i}}}{(N)}}{{\,\mathrm{\delta }\,}}_{\psi '}(u,w)$. By Lemma 6 (with $Z=\hbox {Succ}_{\Gamma }{(x)}$, $Y=\{v_i\}$, $\mathcal {G}=\{N\}$, and $(S^y)_{y\in Y}=(N)_{y\in Y}$), $\psi '$ also minimizes $\sum _{uw\in {A}_{{v_{i}}}{(N)}}{{\,\mathrm{\delta }\,}}_{\psi '}(u,w)$ for all $1\le i\le t$. Thus, the induction hypothesis applies to $T^{\mathcal {HW}}[{v_i,{{\psi _x}\left[ {x\rightarrow c_x}\right] }\mid _{\hbox {YW}_{{v_i}}^{\Gamma }}}]$ for all *i*. Then,

$$\begin{aligned} T^{\mathcal {HW}}[{x,\psi _x}]&{\mathop {=}\limits ^{(10)}} \sum \limits _{1\le i\le t} T^{\mathcal {HW}}[{v_i,{{\psi _x}\left[ {x\rightarrow c_x}\right] }\mid _{\hbox {YW}_{{v_i}}^{\Gamma }}}] + \sum \limits _{z\in \hbox {Pred}_{N}^{\downarrow }{(x)}\cup \hbox {Succ}_{N}^{\uparrow }{(x)}} {{\,\mathrm{\delta }\,}}(c_x,\psi _x(z))\\&{\mathop {=}\limits ^{\text {IH}}} \sum _{1\le i\le t}\;\sum \limits _{uw\in {A}_{{v_{i}}}{(N)}}{{\,\mathrm{\delta }\,}}_{\psi '}(u,w) + \sum \limits _{z\in \hbox {Pred}_{N}^{\downarrow }{(x)}\cup \hbox {Succ}_{N}^{\uparrow }{(x)}} {{\,\mathrm{\delta }\,}}(c_x,\psi 
_x(z))\\&{\mathop {=}\limits ^{\psi _x={\psi '}\mid _{\hbox {YW}_{x}^{\Gamma }}}} \sum _{1\le i\le t}\;\sum \limits _{uw\in {A}_{{v_{i}}}{(N)}}{{\,\mathrm{\delta }\,}}_{\psi '}(u,w) + \sum \limits _{z\in \hbox {Pred}_{N}^{\downarrow }{(x)}\cup \hbox {Succ}_{N}^{\uparrow }{(x)}} {{\,\mathrm{\delta }\,}}_{\psi '}(x,z)\\&= \sum \limits _{uw\in {A}_{x}{(N)}}{{\,\mathrm{\delta }\,}}_{\psi '}(u,w) \end{aligned}$$

Since, by assumption, $${T^{\mathcal {HW}}[{x,\psi _x}]}$$ is strictly less than the cost of $$\psi $$ on $$ {A}_{x}{(N)}$$, we conclude that the cost of $$\psi '$$ on $$ {A}_{x}{(N)}$$ is strictly less than that of $$\psi $$, contradicting optimality of $$\psi $$.$$\square $$

### Lemma 8

*Let* $\Gamma$* be a tree that agrees with **N*, let $x\in V(N)$,* let *$\psi _x:\hbox {YW}_{x}^{\Gamma }\rightarrow C$* with* $\psi _x\trianglelefteq \phi$,* and let* $R^x\subseteq \hbox {Succ}_{N}^{R\uparrow }{(\Gamma _x)}$.* If* $\mathcal {S}^{\Gamma _x\rightarrow R^x}(N)=\varnothing$,* then* $T^{\mathcal {SW}}[{x,\psi _x,R^x}=\infty ]$.* Otherwise, let* $S\in \mathcal {S}^{\Gamma _x\rightarrow R^x}(N)$* and* $\psi :V(N)\rightarrow C$* such that (a)* $\psi _x\trianglelefteq \psi \trianglelefteq \phi$* and (b)* $\sum _{uw\in {A}_{x}{(S)}}{{\,\mathrm{\delta }\,}}_\psi (u,w)$* is minimum among all such* *S* and $\psi$.* Then,*

6 $$\begin{aligned} T^{\mathcal {SW}}[{x,\psi _x,R^x}]=\sum \limits _{uw\in A_x({S})}{{\,\mathrm{\delta }\,}}_\psi (u,w). \end{aligned}$$

### Proof

Note that arcs that are incoming to tree nodes cannot be switched off and, thus, $\hbox {Succ}_{N}^{T\uparrow }{(z)}=\hbox {Succ}_{S'}^{T\uparrow }{(z)}$ for all $z\in V(N)$ and all switchings $S'\in \mathcal {S}(N)$. The proof is by induction on the height of *x* in $\Gamma$.

**Case 1:** *x* is a leaf in $\Gamma$, that is, $t=0$. First, note that $R^x\subseteq \hbox {Succ}_{N}^{R\uparrow }{(x)}$ and no $r\in R^x\subseteq R(N)$ can have all their parents in $\Gamma _x=\{x\}$, thus implying $\mathcal {S}^{x\rightarrow R^x}(N)\ne \varnothing$. Next, let *y* be the predecessor of *x* in *S* and note that $y\in \hbox {Pred}_{N}^{\downarrow }{(x)}=\hbox {Pred}_{N}{(x)}$. Further, *y* minimizes ${{\,\mathrm{\delta }\,}}_\psi (y,x)$ among all $y\in \hbox {Pred}_{N}{(x)}$ as, otherwise, we can construct a new switching $S'\in \mathcal {S}^{\Gamma _x\rightarrow R^x}(N)$ by replacing *yx* by some $y'x$ with $y'\in \hbox {Pred}_{N}{(x)}$, thereby contradicting (b). Clearly, $\hbox {Pred}_{N}^{\uparrow }{(x)}=\varnothing$ and $Q_{{x,c_x}}^{{\psi _x}}[{0,R^x\setminus R^*}]\ne \infty$ only if $R^*=R^x$. Thus,

$$\begin{aligned} T^{\mathcal {SW}}[{x,\psi _x,R^x}]&{\mathop {=}\limits ^{(4)}} \min _{c_x\in \phi (x)} \left( \sum \limits _{r\in R^x\cup \hbox {Succ}_{N}^{T\uparrow }{(x)}}{{\,\mathrm{\delta }\,}}(c_x,\psi _x(r)) + \min \limits _{y\in \hbox {Pred}_{N}^{\downarrow }{(x)}}{{\,\mathrm{\delta }\,}}(c_x,\psi _x(y)) \right) \\&{\mathop {\le }\limits ^{\psi (x)\in \phi (x)}} \sum \limits _{r\in R^x\cup \hbox {Succ}_{N}^{T\uparrow }{(x)}}{{\,\mathrm{\delta }\,}}(\psi (x),\psi _x(r)) + \min \limits _{yx\in A^{\downarrow }_{x}{(N)}}{{\,\mathrm{\delta }\,}}(\psi (x),\psi _x(y))\\&= \sum \limits _{xr\in A^{\uparrow }_{x}{(S)}}{{\,\mathrm{\delta }\,}}_\psi (x,r) + \sum \limits _{yx\in A^{\downarrow }_{x}{(S)}}{{\,\mathrm{\delta }\,}}_\psi (y,x) = \sum \limits _{uw\in {A}_{x}{(S)}}{{\,\mathrm{\delta }\,}}_\psi (u,w) \end{aligned}$$

and there is some $c_x\in \phi (x)$ such that equality holds if $\psi (x)=c_x$. Let $\psi ^*:={\psi }\left[ {x\rightarrow c_x}\right]$ be the result of changing the assignment of *x* to $c_x$ in $\psi$ and note that $\psi _x\trianglelefteq \psi ^*$. Clearly, we still have $S\in \mathcal {S}^{\Gamma _x\rightarrow R^x}(N)$. Thus,

$$\begin{aligned} T^{\mathcal {SW}}[{x,\psi _x,R^x}]&{\mathop {=}\limits ^{(4)}} \sum \limits _{r\in R^x\cup \hbox {Succ}_{N}^{T\uparrow }{(x)}}{{\,\mathrm{\delta }\,}}(c_x,\psi _x(r)) + \min _{yx\in A^{\downarrow }_{x}{(N)}}{{\,\mathrm{\delta }\,}}(c_x,\psi _x(y))\\&{\mathop {=}\limits ^{\psi _x\trianglelefteq \psi ^*}} \sum \limits _{xr\in A^{\uparrow }_{x}{(S)}}{{\,\mathrm{\delta }\,}}_{\psi ^*}(x,r) + \sum \limits _{yx\in A^{\downarrow }_{x}{(S)}}{{\,\mathrm{\delta }\,}}_{\psi ^*}(y,x)\\&= \sum \limits _{uw\in {A}_{x}{(S)}}{{\,\mathrm{\delta }\,}}_{\psi ^*}(u,w) {\mathop {\ge }\limits ^{\text {Lemma 8(b)}}} \sum \limits _{uw\in {A}_{x}{(S)}}{{\,\mathrm{\delta }\,}}_\psi (u,w) \end{aligned}$$

**Case 2:** *x* has children $v_1$, $v_2$, ..., $v_t$ in $\Gamma$. Recall that we suppose that $x\in \bigcup _{i\le t}\hbox {YW}_{{v_i}}^{\Gamma }$ by Lemma 3. For all $S^*\in \mathcal {S}(N)$ and all anti-chains *Y* in $\Gamma$, abbreviate $\mathcal {S}^{Y\rightarrow \bigcup _{y\in Y}\hbox {Succ}_{{S^*}}^{R\uparrow }{(\Gamma _y)}}(N)=:\mathcal {S}^{Y,S^*}(N)$, that is, roughly, the set of switchings of *N* with the same “behavior” as $S^*$ on *Y*. The proof of Case 2 relies on the independence of partial solutions established by Lemma 6 with $\mathcal {G}=\mathcal {S}^{Y, S^*}(N)$. To apply Lemma 6, we show that any set of switchings $S^y$ such that $\{\hbox {Succ}_{S^y}^{R\uparrow }{(\Gamma _y)}\mid y\in Y\}$ is a partition of $\bigcup _{y\in Y}\hbox {Succ}_{S^*}^{R\uparrow }{(\Gamma _y)}$ is a *Y*-substitution system for $\mathcal {S}^{Y,S^*}(N)$.

### Claim 4

Let $S^*\in \mathcal {S}(N)$ and let *Y* be an anti-chain in $\Gamma$. For each $y\in Y$, let $S^y\in \mathcal {S}(N)$ such that $\{\hbox {Succ}_{S^y}^{R\uparrow }{(\Gamma _y)} \mid y\in Y\}$ is a partition of $\bigcup _{y\in Y}\hbox {Succ}_{S^*}^{R\uparrow }{(\Gamma _y)}$. Let

$$\begin{aligned} S':=\left( V(N), \left( A(S^*)\setminus \bigcup _{y\in Y} {A}_{y}{(S^{*})} \right) \cup \bigcup _{y\in Y} {A}_{y}{(S^{y})}\right) \end{aligned}$$

Then, $$S'\in \mathcal {S}^{Y,S^*}(N)$$.

### Proof

Since $\{\hbox {Succ}_{S_i}^{R\uparrow }{(\Gamma _y)} \mid y\in Y\}$ is a partition of $\bigcup _{y\in Y}\hbox {Succ}_{S^*}^{R\uparrow }{(\Gamma _y)}$, it is sufficient to show that $S'\in \mathcal {S}(N)$. Towards a contradiction, assume there is a node $w\in V(N)-\rho _N$ that does not have exactly one parent in $S'$ and let $u^*$ be the parent of *w* in $S^*$. Clearly, for each $y\in Y$, we have $w\notin \Gamma _y$ as, otherwise, $\hbox {Pred}_{S'}{(w)}=\hbox {Pred}_{S^y}{(w)}$. Further, $w\in \bigcup _{y\in Y}\hbox {YW}_{{y}}^{\Gamma }$ as, otherwise, $\hbox {Pred}_{S'}{(w)}=\hbox {Pred}_{S^*}{(w)}$.

First, suppose *w* has no parent in $S'$. Then, $u^*w\in \bigcup _{y\in Y} {A}_{y}{(S^{*})}$ that is, $u^*\in \Gamma _y$ for some $y\in Y$, but $w\notin {A}_{y}{(S^{y})}$. But since $S^y\in \mathcal {S}(N)$, we know that *w* has a parent in $S^y$ (which is not $u^*$ since $w\notin {A}_{y}{(S^{y})}$), implying that *w* is a reticulation in *N*. Thus, $w\in \hbox {Succ}_{S^*}^{R\uparrow }{(\Gamma _y)}\subseteq \bigcup _{y'\in Y}\hbox {Succ}_{S_{y'}}^{R\uparrow }{(\Gamma _{y'})}$ so there is some $y'\in Y$ with $w\in \hbox {Succ}_{S_{y'}}^{R\uparrow }{(\Gamma _{y'})}$ (note that $y\ne y'$ is possible). But then, $S_{y'}$ contains an arc $uw\in {A}_{y'}{(S_{y'})}$ which is in $S'$ by construction, thus contradicting *w* having no parents in $S'$.

Second, suppose that *w* has at least two distinct parents *u* and $u^*$ in $S'$ and note that, again, *w* is a reticulation in *N*. Since $S^*$ is a switching, at least one of them, say *u*, is such that $uw\in \bigcup _{y\in Y} {A}_{y}{(S^{y})}$. However, since the $\hbox {Succ}_{S^{y}}^{R\uparrow }{(\Gamma _y)}$ are disjoint and each $S^y$ is a switching, we cannot have $u^*w\in \bigcup _{y\in Y} {A}_{y}{(S^{y})}$. Thus, $u^*w\in A(S^*)\setminus \bigcup _{y\in Y} {A}_{y}{(S^{*})}$. However, since $\bigcup _{y\in Y}\hbox {Succ}_{S^{*}}^{R\uparrow }{(\Gamma _y})=\bigcup _{y\in Y}\hbox {Succ}_{S^{y}}^{R\uparrow }{(\Gamma _y})$, we know that $uw\in {A}_{{S^*}}{(\Gamma _y)}$ for some $y\in Y$. But then, *w* has two parents in $S^*$ contradicting $S^*\in \mathcal {S}(N)$.$\square$

In the following, we prove the semantics of the table $Q_{{x,c_x}}^{{\psi _x}}$. For all $i\le t$, abbreviate $\bigcup _{1\le j\le i}\Gamma _{v_j}=:Z_i$.

### Claim 5

Let $1\le i\le t$, let $c_x\in \phi (x)$, and let $R'\subseteq R(N)$. If $\mathcal {S}^{Z_i\rightarrow R'}(N)=\varnothing$, then $Q_{{x,c_x}}^{{\psi _x}}[{i,R'}]=\infty$. Otherwise, let $S_i\in \mathcal {S}^{Z_i\rightarrow R'}(N)$ and $\psi _i:V(N)\rightarrow C$ such that (a) $\psi _i\trianglelefteq \phi$, (b) $\psi _i$ coincides with ${\psi _x}\left[ {x\rightarrow c_x}\right]$ on $\bigcup _{j\le i}\hbox {YW}_{{v_j}}^{\Gamma }$ and (c) $\sum _{j\le i}\sum _{uw\in {A}_{{v_j}}{(S_i)}}{{\,\mathrm{\delta }\,}}_{\psi _i}(u,w)$ is minimum among all such $S_i$ and $\psi _i$ and

$$\begin{aligned} Q_{{x,c_x}}^{{\psi _x}}[{i,R'}] = \sum _{j\le i}\sum \limits _{uw\in {A}_{{v_j}}{(S_i)}}{{\,\mathrm{\delta }\,}}_{\psi _i}(u,w) \end{aligned}$$

### Proof

The proof is by induction on *i*, noting that ${{\psi _x}\left[ {x\rightarrow c_x}\right] }\mid _{\hbox {YW}_{{v_i}}^{\Gamma }} = {\psi _i}\mid _{\hbox {YW}_{{v_1}}^{\Gamma }}$ by Claim 5(b).

**Case** $i=1$: By (5), $Q_{{x,c_x}}^{{\psi _x}}[{0,R'\setminus R^*}]\ne \infty$ only if $R^*=R'\subseteq \hbox {Succ}_{N}^{R\uparrow }{(Z_1)}$ and ${T^{\mathcal {SW}}[{v_1,{\psi _1}\mid _{\hbox {YW}_{{v_1}}^{\Gamma }},R^*}]\ne \infty }$. However, if $\mathcal {S}^{Z_i\rightarrow R'}(N)=\varnothing$ then, by induction hypothesis (of the lemma), ${T^{\mathcal {SW}}[{v_1,{\psi _1}\mid _{\hbox {YW}_{{v_1}}^{\Gamma }},R'}]=\infty }$ and so $Q_{{x,c_x}}^{{\psi _x}}[{0,R'\setminus R^*}]=\infty$. Furthermore, $S_1$, $\psi _1$, and $R'$ satisfy the conditions of the lemma for $v_1$, so we can employ the induction hypothesis of the lemma. Thus,

$$\begin{aligned} Q_{{x,c_x}}^{{\psi _x}}[{1,R'}]&= 0 + T^{\mathcal {SW}}[{v_1,{\psi _1}\mid _{\hbox {YW}_{{v_1}}^{\Gamma }},R'}] {\mathop {=}\limits ^{\text {IH lemma}}} \sum \limits _{uw\in {A}_{{v_1}}{(S_1)}}{{\,\mathrm{\delta }\,}}_{\psi _1}(u,w) \end{aligned}$$

**Case** $i>1$: First, by (5), $Q_{{x,c_x}}^{{\psi _x}}[{i,R'}]\ne \infty$ only if $Q_{{x,c_x}}^{{\psi _x}}[{i-1,R'\setminus R^*}]\ne \infty$ and ${T^{\mathcal {SW}}[{v_i,{\psi _i}\mid _{\hbox {YW}_{{v_i}}^{\Gamma }},R^*}]\ne \infty }$. By induction hypotheses (of the claim and the lemma), there are switchings $S_{i-1}$ and $S'$ of *N* with $\hbox {Succ}_{S_{i-1}}^{R\uparrow }{(Z_{i-1})}=R'\setminus R^*$ and $\hbox {Succ}_{S'}^{R\uparrow }{(\Gamma _{v_i})}=R^*$. Now, we construct a digraph $S_i:=(V(N),(A(S_{i-1}\setminus {A}_{{v_i}}{(S_{i-1})})\cup {A}_{{v_i}}{(S')})$ and show that $S_i\in \mathcal {S}^{Z_i\rightarrow R'}(N)$. Since $\hbox {Succ}_{S_{i}}^{R\uparrow }{(Z_i)}=\hbox {Succ}_{S_{i-1}}^{R\uparrow }{(Z_{i-1})}\uplus \hbox {Succ}_{S'}^{R\uparrow }{(\Gamma _{v_i})}=(R'\setminus R^*)\uplus R^*=R'$, it is sufficient to show that $S_i$ can be turned into a switching of *N* without changing $\hbox {Succ}_{S_{i}}^{R\uparrow }{(Z_i)}$. To this end, suppose that there is a node $w\ne \rho _N$ of *N* that does not have exactly one parent in $S_i$. Since $S_{i-1}$ and $S'$ are switchings, *w* has parents $u_{i-1}$ and $u'$ in $S_{i-1}$ and $S'$, respectively. If *w* has no parent in $S_i$, then $u_{i-1}w\in {A}_{{v_i}}{(S_{i-1})}$ and $u'w\notin {A}_{{v_i}}{(S')}$ and, thus, $u_{i-1} \le _\Gamma v_i <_\Gamma u'$, implying $u'\ne u_{i-1}$ as well as $w\in \hbox {YW}_{{v_i}}^{\Gamma }$ and $w\notin R'$. Then, we can just add the arc $u'w$ to $S_i$ without changing $\hbox {Succ}_{S_{i}}^{R\uparrow }{(Z_i)}$. If *w* has at least two parents, then $u_{i-1}$ and $u'$ are both parents of *w* in $S_i$, that is, $u_{i-1}w\notin {A}_{{v_i}}{(S_{i-1})}$ and $u'w\in {A}_{{v_i}}{(S')}$ and, thus, $u'<_\Gamma v_i <_\Gamma u_{i-1}$, implying $u'\ne u_{i-1}$ as well as $w\in \hbox {YW}_{{v_i}}^{\Gamma }$ and $w\in R^*$. But then, we can remove $u_{i-1}w$ from $S_i$ without changing $\hbox {Succ}_{S_{i}}^{R\uparrow }{(v_i)}$. Repeating this argument, we can turn $S_i$ into a switching of *N* with $\hbox {Succ}_{S_{i}}^{R\uparrow }{(Z_i)}=R'$, implying that $\mathcal {S}^{Z_i\rightarrow R'}(N)\ne \varnothing$. For the second part of the claim, we show both inequalities separately.

“$\le$”: Let $S_i\in \mathcal {S}^{Z_i\rightarrow R'}(N)$ and $\psi _i:V(N)\rightarrow C$ $\psi _i$ coincides with ${\psi _x}\left[ {x\rightarrow c_x}\right]$ on $\bigcup _{j\le i}\hbox {YW}_{{v_j}}^{\Gamma }$ and $\sum _{j\le i}\sum _{uw\in {A}_{{v_j}}^{(S_i)}}{{\,\mathrm{\delta }\,}}_{\psi _i}(u,w)$ is minimum among all such $S_i$ and $\psi _i$. Further, let $R^*:=\hbox {Succ}_{S_{i}}^{R\uparrow }{(\Gamma _{v_i})}$. Note that $\hbox {Succ}_{S_{i}}^{R\uparrow }{(Z_{i-1})}$ and $\hbox {Succ}_{S_{i}}^{R\uparrow }{(v_i)}=R^*$ are disjoint since $S_i$ is a switching, implying $\hbox {Succ}_{S_{i}}^{R\uparrow }{(Z_{i-1})} = R'\setminus R^*$ and, thus, $Q_{{x,c_x}}^{{\psi _x}}[{i-1,R'\setminus R^*}]$ and ${T^{\mathcal {SW}}[{v_i,\phi _i,R^*}]}$ are finite by induction hypotheses. Then, as $R^*\subseteq R'\cap \hbox {Succ}_{N}^{R\uparrow }{(\Gamma _{v_i})}$, we know that $R^*$ participates in the minimum of (5). Thus,

$$\begin{aligned} Q_{{x,c_x}}^{{\psi _x}}[{i,R'}]&\le Q_{{x,c_x}}^{{\psi _x}}[{i-1,R'\setminus R^*}] + T^{\mathcal {SW}}[{v_i,{\psi _i}\mid _{\hbox {YW}_{{v_i}}^{\Gamma }},R^*}]\\&{\mathop {\le }\limits ^{\begin{array}{c} \text {IH claim}\\ \text {IH lemma}\\ \end{array}}} \sum \limits _{j\le i-1}\sum \limits _{uw\in {A}_{{v_j}}{(S_i)}}{{\,\mathrm{\delta }\,}}_{\psi _i}(u,w) + \sum \limits _{uw\in {A}_{{v_i}}{(S_i)}}{{\,\mathrm{\delta }\,}}_{\psi _i}(u,w)\\&= \sum _{j\le i}\sum \limits _{uw\in {A}_{{v_j}}{(S_i)}}{{\,\mathrm{\delta }\,}}_{\psi _i}(u,w) \end{aligned}$$

“$$\ge $$”: Clearly, this direction is trivial if $$Q_{{x,c_x}}^{{\psi _x}}[{i,R'}]$$ is infinite, so suppose it is finite. By (5), there is some $$R^*\subseteq R'\cap \hbox {Succ}_{N}^{R\uparrow }{(\Gamma _{v_i})}$$ with $${Q_{{x,c_x}}^{{\psi _x}}[{i,R'}]=Q_{{x,c_x}}^{{\psi _x}}[{i-1,R'\setminus R^*}] + T^{\mathcal {SW}}[{v_i,{\psi _i}\mid _{\hbox {YW}_{{v_i}}^{\Gamma }},R^*}]}$$. First, since $${T^{\mathcal {SW}}[{v_i,{\psi _i}\mid _{\hbox {YW}_{{v_i}}^{\Gamma }},R^*}]\ne \infty }$$, the induction hypothesis (of the lemma) guarantees that there is some $$S^*\in \mathcal {S}^{\Gamma _{v_i}\rightarrow R^*}(N)$$ and $$\psi ^*:V(N)\rightarrow C$$ such that (a) $${\psi _i}\mid _{\hbox {YW}_{{v_i}}^{\Gamma }}\trianglelefteq \psi ^*\trianglelefteq \phi $$, (b) $$(S^*,\psi ^*)$$ is optimal on $$ {A}_{{v_i}}{(S^*)}$$, and (c) $${T^{\mathcal {SW}}[{v_i,{\psi _i}\mid _{\hbox {YW}_{{v_i}}^{\Gamma }},R^*}]=\sum _{uw\in {A}_{{v_i}}{(S^*)}}{{\,\mathrm{\delta }\,}}_{\psi ^*}(u,w)}$$. Second, since $$Q_{{x,c_x}}^{{\psi _x}}[{i-1,R'\setminus R^*}]\ne \infty $$, the induction hypothesis (of the claim) guarantees that there are $$S_{i-1}\in \mathcal {S}^{Z_{i-1}\rightarrow R'\setminus R^*}(N)$$ and $$\psi _{i-1}:V(N)\rightarrow C$$ such that (a) $$\psi _{i-1}\trianglelefteq \phi $$, (b) $$\psi _{i-1}$$ coincides with $${\psi _x}\left[ {x\rightarrow c_x}\right] $$ on $$\bigcup _{j<i}\hbox {YW}_{{v_j}}^{\Gamma }$$, (c) $$\sum _{j<i}\sum _{uw\in {A}_{{v_j}}{(S_{i-1})}}{{\,\mathrm{\delta }\,}}_{\psi _{i-1}}(u,w)$$ is minimal among all such $$S_{i-1}$$ and $$\psi _{i-1}$$, and (d) $$Q_{{x,c_x}}^{{\psi _x}}[{i-1,R'\setminus R^*}]=\sum _{j<i}\sum _{uw\in {A}_{{v_j}}{(S_{i-1})}}{{\,\mathrm{\delta }\,}}_{\psi _{i-1}}(u,w)$$. Finally, we construct a new solution $$S'$$ by replacing $$S_i$$ by $$S^*$$ on $$\Gamma _{v_i}$$ and by $$S_{i-1}$$ on $$Z_{i-1}$$ and we use Claim 5(c) to show that the cost of $$S_i$$ is at most that of $$S'$$. More formally, let

11 $$\begin{aligned} S' := \left( V(N), \left( A(S_i)\setminus \bigcup _{j\le i} {A}_{{v_j}}{(S_i)}\right) \cup \bigcup _{j<i} {A}_{{v_j}}{(S_{i-1})} \cup {A}_{{v_i}}{(S^*)}\right) \end{aligned}$$

Since $$\{v_1,v_2,\ldots ,v_i\}$$ is an anti-chain in $$\Gamma $$ and $$\{\hbox {Succ}_{S_{i-1}}^{R\uparrow }{(Z_{i-1})}, \hbox {Succ}_{S^{*}}^{R\uparrow }{(\Gamma _{v_i})}\}=\{R^*,R'\setminus R^*\}$$ is a partition of $$\hbox {Succ}_{S_{i}}^{R\uparrow }{(Z_i)}=R'$$, Claim 4 implies $$S'\in \mathcal {S}^{Z_i\rightarrow R'}(N)$$. Further, let $$\psi ':V(N)\rightarrow C$$ such that, for all $$a\in A(S')$$, $$\psi '(a):=\psi _{i-1}(a)$$ if $$a\in {A}_{{Z_i}}{(S_{i-1})}$$, $$\psi '(a):=\psi ^*(a)$$ if $$a\in {A}_{{v_i}}{(S^*)}$$, and $$\psi '(a):=\psi _i(a)$$, otherwise. Note that $$\psi '\trianglelefteq \phi $$. Further, $$\psi _i$$ and $$\psi _{i-1}$$ coincide on $$\hbox {YW}_{{Z_{i-1}}}^{\Gamma }$$ and, thus, $$\psi '$$ and $$\psi _{i-1}$$ coincide on all nodes touched by $$ {A}_{{Z_{i-1}}}{(S')}= {A}_{{Z_{i-1}}}{(S_{i-1})}$$. Further, $$\psi _i$$ and $$\psi ^*$$ coincide on $$\hbox {YW}_{{v_i}}^{\Gamma }$$ and, thus, $$\psi '$$ and $$\psi ^*$$ coincide on all nodes touched by $$ {A}_{{v_i}}{(S')}= {A}_{{v_i}}{(S^*)}$$. Thus,

$$\begin{aligned} Q_{{x,c_x}}^{{\psi _x}}[{i,R'}]&= Q_{{x,c_x}}^{{\psi _x}}[{i-1,R'\setminus R^*}] + T^{\mathcal {SW}}[{v_i,{\psi ^*}\mid _{\hbox {YW}_{{v_i}}^{\Gamma }},R^*}]\\&{\mathop {=}\limits ^{(c),(g)}} \sum _{j<i}\sum \limits _{uw\in {A}_{{v_j}}{(S_{i-1})}}{{\,\mathrm{\delta }\,}}_{\psi _{i-1}}(u,w) + \sum \limits _{uw\in {A}_{{v_i}}{(S^*)}}{{\,\mathrm{\delta }\,}}_{\psi ^*}(u,w)\\&{\mathop {=}\limits ^{\text {df.}\,\psi ', (11)}} \sum _{j<i}\sum \limits _{uw\in {A}_{{v_j}}{(S')}}{{\,\mathrm{\delta }\,}}_{\psi '}(u,w) + \sum \limits _{uw\in {A}_{{v_i}}{(S')}}{{\,\mathrm{\delta }\,}}_{\psi '}(u,w)\\&= \sum \limits _{uw\in {A}_{{Z_i}}{(S')}}{{\,\mathrm{\delta }\,}}_{\psi '}(u,w) {\mathop {\ge }\limits ^{\text {Claim 5(c)}}} \sum _{j\le i}\sum \limits _{uw\in {A}_{{v_j}}{(S_i)}}{{\,\mathrm{\delta }\,}}_{\psi _i}(u,w) \end{aligned}$$

$$\square $$

Having established the semantics of $Q_{{x,c_x}}^{{\psi _x}}$, we can finish proving Case 2 of Lemma 8s. First, consider the case that $\mathcal {S}^{\Gamma _x\rightarrow R^x}(N)=\varnothing$ and assume that ${T^{\mathcal {SW}}[{x,\psi _x,R^x}]\ne \infty }$. By Eq. (4) and Claim 5, there is some $c_x$ and $R^*\subseteq R^x\cap \hbox {Succ}_{N}^{R\uparrow }{(x)}$ such that $\mathcal {S}^{Z_t\rightarrow R^x\setminus R^*}(N)\ne \varnothing$ or $\mathcal {S}^{Z_t\rightarrow (R^x\setminus R^*)\cup (\{x\}\cup R(N))}(N)\ne \varnothing$. Let $S'$ be a switching in one of these sets and note that $\hbox {Succ}_{S'}^{R\uparrow }{(\Gamma _x)}=R^x\setminus R^*$. If there is some $y\in R^x\setminus \hbox {Succ}_{S'}^{R\uparrow }{(\Gamma _x)}$, then $y\in R^*$ and $S'$ contains an arc *zy* for some $z\notin \Gamma _x$, implying that we can swap *zy* for *xy* in $S'$ without affecting $\hbox {Succ}_{S'}^{R\uparrow }{(Z_t)}$ or $S'$ being a switching. Thus, we can assume without loss of generality that $\hbox {Succ}_{S'}^{R\uparrow }{(\Gamma _x)}=R^x$. But then, $S'\in \mathcal {S}^{\Gamma _x\rightarrow R^x}$ contradicting $\mathcal {S}^{\Gamma _x\rightarrow R^x}=\varnothing$. In the following, we thus assume that $\mathcal {S}^{\Gamma _x\rightarrow R^x}\ne \varnothing$ and we show both directions of the lemma separately.

“$\le$”: Let $c_x:=\psi (x)\in \phi (x)$, let $R^*:=\hbox {Succ}_{S}^{R\uparrow }{(x)}$, and note that $R^*=\hbox {Succ}_{S}^{R\uparrow }{(x)}\subseteq \hbox {Succ}_{S}^{R\uparrow }{(\Gamma _x)}=R^x$. Further, let $y:=\hbox {Pred}_{S}{(x)}$ be the parent of *x* in *S*. Since $\Gamma$ agrees with *N* (and, thus, with *S*) we know that either $x<_\Gamma y$ or $x>_\Gamma y$. If $x<_\Gamma y$, that is, $y\in \hbox {Pred}_{N}^{\downarrow }{(x)}$, then $\hbox {Succ}_{S}^{R\uparrow }{(Z_t)}=\hbox {Succ}_{S}^{R\uparrow }{(\Gamma _x)}\setminus \hbox {Succ}_{S}^{R\uparrow }{(x)} = R^x\setminus R^*$ and, by Claim 5,

12 $$\begin{aligned} \sum \limits _{uw\in {A}_{{Z_t}}{(S)}}{{\,\mathrm{\delta }\,}}_\psi (u,w) + \sum \limits _{uw\in A^{\downarrow }_{\{x\}}{(S)}}{{\,\mathrm{\delta }\,}}_\psi (u,w)&\ge Q_{{x,c_x}}^{{\psi _x}}[{t,R^x\setminus R^*}] + {{\,\mathrm{\delta }\,}}_\psi (x,y)\nonumber \\&\ge Q_{{x,c_x}}^{{\psi _x}}[{t,R^x\setminus R^*}] + {\min _{yx\in A^{\downarrow }_{\{x\}}{(N)}}}{{\,\mathrm{\delta }\,}}(c_x,\psi (y)) \end{aligned}$$

If $x>_\Gamma y$, that is, $y\in \hbox {Pred}_{N}^{\uparrow }{(x)}$, then $\hbox {Succ}_{S}^{R\uparrow }{(Z_t)}=(R(N)\cap \{x\})\cup (\hbox {Succ}_{S}^{R\uparrow }{(\Gamma _x)}\setminus \hbox {Succ}_{S}^{R\uparrow }{(x)}) = (R(N)\cap \{x\})\cup (R^x\setminus R^*)$ and, by Claim 5,

13 $$\begin{aligned} \sum \limits _{uw\in {A}_{{Z_t}}{(S)}}{{\,\mathrm{\delta }\,}}_\psi (u,w) + \sum \limits _{uw\in A^{\downarrow }_{\{x\}}{(S)}}{{\,\mathrm{\delta }\,}}_\psi (u,w) \ge Q_{{x,c_x}}^{{\psi _x}}[{t,(R(N)\cap \{x\})\cup R^x\setminus R^*}] \end{aligned}$$

Then, since $c_x$ and $R^*$ are valid choices for the minima in (4), we have

$$\begin{aligned} T^{\mathcal {SW}}[{x,\psi _x,R^x}]&{\mathop {\le }\limits ^{(4),(12),(13)}} \sum \limits _{r\in R^*\cup \hbox {Succ}_{N}^{T\uparrow }{(x)}}{{\,\mathrm{\delta }\,}}(c_x,\psi (r)) + \sum \limits _{uw\in {A}_{{Z_t}}{(S)}}{{\,\mathrm{\delta }\,}}_\psi (u,w) + \sum \limits _{uw\in A^{\downarrow }_{\{x\}}{(S)}}{{\,\mathrm{\delta }\,}}_\psi (u,w)\\&= \sum \limits _{xr\in A^{\uparrow }_{\{x\}}{(S)}}{{\,\mathrm{\delta }\,}}_\psi (x,r) + \sum \limits _{uw\in {A}_{{Z_t}}{(S)}}{{\,\mathrm{\delta }\,}}_\psi (u,w) + \sum \limits _{uw\in A^{\downarrow }_{\{x\}}{(S)}}{{\,\mathrm{\delta }\,}}_\psi (u,w) = \sum \limits _{uw\in {A}_{x}{(S)}}{{\,\mathrm{\delta }\,}}_\psi (u,w) \end{aligned}$$

“$\ge$”: Suppose that ${T^{\mathcal {SW}}[{x,\psi _x,R^x}]\ne \infty }$ as, otherwise, this direction is trivial. We consider each case of the minimum in (4) individually (although both cases are analogous).

**Case 2.1:** $\hbox {Pred}_{N}^{\downarrow }{(x)}\ne \varnothing$ and there are $c_x\in \phi (x)$ and $R^*\subseteq R^x\cap \hbox {Succ}_{N}^{R\uparrow }{(x)}$ such that

14 $$\begin{aligned} T^{\mathcal {SW}}[{x,\psi _x,R^x}] = \sum \limits _{r\in R^*\cup \hbox {Succ}_{N}^{T\uparrow }{(x)}}{{\,\mathrm{\delta }\,}}(c_x,\psi _x(r)) + Q_{{x,c_x}}^{{\psi _x}}[{t,R^x\setminus R^*}] + \min \limits _{y\in \hbox {Pred}_{N}^{\downarrow }{(x)}}{{\,\mathrm{\delta }\,}}(c_x,\psi _x(y)) \end{aligned}$$

By Claim 5, there is some $S'\in \mathcal {S}^{Z_t\rightarrow R^x\setminus R^*}(N)$ and some $\psi ':V(N)\rightarrow C$ such that (a) $\psi '\trianglelefteq \phi$, (b) $\psi '$ coincides with ${\psi _x}\left[ {x\rightarrow c_x}\right]$ on $\bigcup _{i\le t}\hbox {YW}_{{v_i}}^{\Gamma }$ (recall that $x\in \bigcup _{i\le t}\hbox {YW}_{{v_i}}^{\Gamma }$) (c) $\sum _{uw\in {A}_{{Z_t}}{(S')}}{{\,\mathrm{\delta }\,}}_{\psi '}(u,w)$ is minimum among all such $S'$ and $\psi '$ and

15 $$\begin{aligned} Q_{{x,c_x}}^{{\psi _x}}[{t,R^x\setminus R^*}] = \sum \limits _{uw\in {A}_{{Z_t}}{(S')}}{{\,\mathrm{\delta }\,}}_{\psi '}(u,w) \end{aligned}$$

From $$S'$$ we construct a switching $$S^*\in \mathcal {S}^{\Gamma _x\rightarrow R^x}(N)$$ by 1. swapping each arc $$zr\in A(S')$$ with $$r\in R^*$$ for *xr* (which exists in *N* since $$R^*\subseteq \hbox {Succ}_{N}^{R\uparrow }{(x)}$$), 2. swapping each arc $$xr\in A(S')$$ with $$r\notin R^x$$ for an arc *zr* with $$z\notin \Gamma _x$$ (which exists in *N* since $$\mathcal {S}^{\Gamma _x\rightarrow R^x}(N)\ne \varnothing $$), and 3. swapping the arc $$yx\in A^{\downarrow }_{\{x\}}{(S')}$$ with an arc $$zx\in \hbox {Pred}_{N}^{\downarrow }{(x)}\times \{x\}$$ minimizing $${{\,\mathrm{\delta }\,}}_{\psi '}(x,z)$$. Since this operation does not change the in-degree of any node, $$S^*$$ is still a switching of *N* and we have $$\hbox {Succ}_{S^{*}}^{R\uparrow }{(x)}=R^*$$ and $$ {A}_{{Z_t}}{(S')}= {A}_{{Z_t}}{(S^{*})}$$ by construction. Thus, $$\hbox {Succ}_{S^{*}}^{R\uparrow }{(Z_t)}=R^x\setminus R^*$$ and $$\hbox {Succ}_{S^{*}}^{R\uparrow }{(\Gamma _x)}=R^x$$. Altogether,

$$\begin{aligned} T^{\mathcal {SW}}[{x,\psi _x,R^x}] {\mathop {=}\limits ^{(14),(15)}}&\sum \limits _{r\in R^*\cup \hbox {Succ}_{N}^{T\uparrow }{(x)}}{{\,\mathrm{\delta }\,}}(c_x,\psi _x(r)) + \sum \limits _{uw\in {A}_{{Z_t}}{(S')}}{{\,\mathrm{\delta }\,}}_{\psi '}(u,w) + \min \limits _{y\in \hbox {Pred}_{N}^{\downarrow }{(x)}}{{\,\mathrm{\delta }\,}}(c_x,\psi _x(y))\\ =&\sum \limits _{r\in R^*\cup \hbox {Succ}_{N}^{T\uparrow }{(x)}}{{\,\mathrm{\delta }\,}}(c_x,\psi _x(r)) + \sum \limits _{uw\in {A}_{{Z_t}}{(S^{*})}}{{\,\mathrm{\delta }\,}}_{\psi '}(u,w) + \min \limits _{y\in \hbox {Pred}_{N}^{\downarrow }{(x)}}{{\,\mathrm{\delta }\,}}(c_x,\psi _x(y))\\ {\mathop {=}\limits ^{\psi _x={\psi '}\mid _{\hbox {YW}_{x}^{\Gamma }}}}&\sum \limits _{xr\in A^{\uparrow }_{\{x\}}{(S^*)}}{{\,\mathrm{\delta }\,}}_{\psi '}(x,r) + \sum \limits _{uw\in {A}_{{Z_t}}{(S^{*})}}{{\,\mathrm{\delta }\,}}_{\psi '}(u,w) + \sum \limits _{yx\in A^{\downarrow }_{\{x\}}{(S^*)}}{{\,\mathrm{\delta }\,}}_{\psi '}(y,x)\\ =&\sum \limits _{uw\in {A}_{x}{(S^*)}}{{\,\mathrm{\delta }\,}}_{\psi '}(u,w) {\mathop {\ge }\limits ^{\text {Lemma 8(b)}}} \sum \limits _{uw\in {A}_{x}{(S)}}{{\,\mathrm{\delta }\,}}_\psi (u,w) \end{aligned}$$

**Case 2.2:** $\hbox {Pred}_{N}^{\uparrow }{(x)}\ne \varnothing$ and there are $c_x\in \phi (x)$ and $R^*\subseteq R^x\cap \hbox {Succ}_{N}^{R\uparrow }{(x)}$ such that

16 $$\begin{aligned} T^{\mathcal {SW}}[{x,\psi _x,R^x}] = \sum \limits _{r\in R^*\cup \hbox {Succ}_{N}^{T\uparrow }{(x)}}{{\,\mathrm{\delta }\,}}(c_x,\psi _x(r)) + Q_{{x,c_x}}^{{\psi _x}}[{t,(R(N)\cap \{x\})\cup R^x\setminus R^*}] \end{aligned}$$

Abbreviate $R':=(R(N)\cap \{x\})\cup R^x\setminus R^*$. By Claim 5, there is some $S'\in \mathcal {S}^{Z_t\rightarrow R'}(N)$ and some $\psi ':V(N)\rightarrow C$ such that (a) $\psi '\trianglelefteq \phi$, (b) $\psi '$ coincides with ${\psi _x}\left[ {x\rightarrow c_x}\right]$ on $\bigcup _{i\le t}\hbox {YW}_{{v_i}}^{\Gamma }$, (c) $\sum _{uw\in {A}_{{Z_t}}{(S')}}{{\,\mathrm{\delta }\,}}_{\psi '}(u,w)$ is minimum among all such $S'$ and $\psi '$ and

17 $$\begin{aligned} Q_{{x,c_x}}^{{\psi _x}}[{t,R'}] = \sum \limits _{uw\in {A}_{{Z_t}}{(S')}}{{\,\mathrm{\delta }\,}}_{\psi '}(u,w) \end{aligned}$$

We construct a switching $$S^*\in \mathcal {S}^{\Gamma _x\rightarrow R^x}(N)$$ by 1. swapping each arc $$zr\in A(S')$$ with $$r\in R^*$$ for *xr* (which exists in *N* since $$R^*\subseteq \hbox {Succ}_{N}^{R\uparrow }{(x)}$$) and 2. swapping each arc $$xr\in A(S')$$ with $$r\notin R^x$$ for an arc *zr* with $$z\notin \Gamma _x$$ (which exists in *N* since $$\mathcal {S}^{\Gamma _x\rightarrow R^x}(N)\ne \varnothing $$). Since this operation does not change the in-degree of any node, $$S^*$$ is still a switching of *N* and we have $$\hbox {Succ}_{S^{*}}^{R\uparrow }{(x)}=R^*$$ and $$ {A}_{{Z_t}}{(S')}= {A}_{{Z_t}}{(S^{*})}$$ by construction. Thus, $$\hbox {Succ}_{S^{*}}^{R\uparrow }{(Z_t)}=R'$$ and $$\hbox {Succ}_{S^{*}}^{R\uparrow }{(\Gamma _x)}=R^x$$. Further, note that if *x* is a tree node, then $$\hbox {Pred}_{N}^{\uparrow }{(x)}\ne \varnothing $$ implies $$A^{\downarrow }_{\{x\}}{(S^*)}\subseteq A^{\downarrow }_{\{x\}}{(N)}=\varnothing $$ and, otherwise, $$x\in R'$$ implying $$A^{\downarrow }_{\{x\}}{(S^*)}=\varnothing $$. Altogether,

$$\begin{aligned} T^{\mathcal {SW}}[{x,\psi _x,R^x}]&{\mathop {=}\limits ^{(16),(17)}} \sum \limits _{r\in R^*\cup \hbox {Succ}_{N}^{T\uparrow }{(x)}}{{\,\mathrm{\delta }\,}}(c_x,\psi _x(r)) + \sum \limits _{uw\in {A}_{{Z_t}}{(S')}}{{\,\mathrm{\delta }\,}}_{\psi '}(u,w)\\&= \sum \limits _{r\in R^*\cup \hbox {Succ}_{N}^{T\uparrow }{(x)}}{{\,\mathrm{\delta }\,}}(c_x,\psi _x(r)) + \sum \limits _{uw\in {A}_{{Z_t}}{(S^{*})}}{{\,\mathrm{\delta }\,}}_{\psi '}(u,w)\\&{\mathop {=}\limits ^{\psi _x={\psi '}\mid _{\hbox {YW}_{x}^{\Gamma }}}} \sum \limits _{xr\in A^{\uparrow }_{\{x\}}{(S^*)}}{{\,\mathrm{\delta }\,}}_{\psi '}(x,r) + \sum \limits _{uw\in {A}_{{Z_t}}{(S^{*})}}{{\,\mathrm{\delta }\,}}_{\psi '}(u,w)\\&{\mathop {=}\limits ^{A^{\downarrow }_{\{x\}}{(S^*)}=\varnothing }} \sum \limits _{uw\in {A}_{x}{(S^*)}}{{\,\mathrm{\delta }\,}}_{\psi '}(u,w) {\mathop {\ge }\limits ^{\text {Lemma 8(b)}}} \sum \limits _{uw\in {A}_{x}{(S)}}{{\,\mathrm{\delta }\,}}_\psi (u,w) \end{aligned}$$

$$\square $$

### Lemma 9

*Let* $x\in V(N)$,* let *$i\in \mathbb {N}$,* let *$\lambda :\hbox {YW}_{x}^{\Gamma }\rightarrow 2^{C}$,* let *$\eta ,\eta ':\hbox {YW}_{x}^{\Gamma }\rightarrow \mathbb {N}$,* and let *$\psi ,\psi ':\hbox {YW}_{x}^{\Gamma }\rightarrow 2^{C}$* such that* $\psi '\trianglelefteq \psi \trianglelefteq \lambda$* and* ${\overrightarrow{0}}\left[ {x\rightarrow \rho (x)}\right] \trianglelefteq \eta '\trianglelefteq \eta$.* Then,*

$$\begin{aligned} T^{\mathcal {PT}}[{x,\lambda ,\psi ',\eta '}] \le T^{\mathcal {PT}}[{x,\lambda ,\psi ,\eta }]&\text {and}&Q_{x}^{\lambda }[{i,\psi ',\eta '}] \le Q_{x}^{\lambda }[{i,\psi ,\eta }] \end{aligned}$$

### Proof

Note that the inequality on $Q^\lambda _x$ trivially holds if $Q_{x}^{\lambda }[{i,\psi ,\eta }]=\infty$ and, similarly for $T^{\mathcal {PT}}$. The proof is based on the observation that the transformations done to $\psi$ and $\eta$ in Equations (7) and (8) are monotone.

### Claim 6

Let $U,D\in \mathbb {N}$. The following functions (acting on functions) are montone

$$\begin{aligned} f_{U,D}(\psi ) := {\psi }\left[ {\begin{array}{l} x\rightarrow D\\ \forall _{y\in \hbox {Succ}_{N}^{\uparrow }{(x)}}y\rightarrow \psi (y)\setminus U \end{array}} \right] \quad g_{U,D}(\eta ) := {\eta }\left[ {\begin{array}{l} x\rightarrow |U| {\mathop {-}\limits ^{.}}\sum _{u\in \hbox {Pred}_{N}^{\downarrow }{(x)}}|\lambda _x(u)|\\ \forall _{y\in \hbox {Succ}_{N}^{\uparrow }{(x)}}y\rightarrow \eta (y){\mathop {-}\limits ^{.}}|U| \end{array}}\right] \end{aligned}$$

### Proof

Let $\psi ,\psi ':\hbox {YW}_{x}^{\Gamma }\rightarrow 2^{C}$ with $\psi '\trianglelefteq \psi$. Then, for all $y\in \hbox {YW}_{x}^{\Gamma }$, Further, for all $y\in \hbox {Succ}_{N}^{\uparrow }{(x)}$, we have $f(\psi ')(y) = \psi '(y)\setminus U \subseteq \psi (y)\setminus U=f(\psi )(y)$.

$$\begin{aligned} f(\psi ')(y)&= \left\{ \begin{array}{ll} D &{} \text {if}\,x=y\\ \psi '(y)\setminus U &{} \text {if}\,y\in \hbox {Succ}_{N}^{\uparrow }{(x)}\\ \psi '(y) &{} \text {otherwise} \end{array}\right. \trianglelefteq \left\{ \begin{array}{ll} D &{} \text {if}\,x=y\\ \psi (y)\setminus U &{} \text {if}\,y\in \hbox {Succ}_{N}^{\uparrow }{(x)}\\ \psi (y) &{} \text {otherwise} \end{array}\right. \\&= f(\psi )(y) \end{aligned}$$

The proof for $g_{U,D}$ is completely analogous.$\square$

With Claim 6, we can show that monotonicity of $Q^\lambda _x$ implies monotonicity of $T^{\mathcal {PT}}$.

### Claim 7

Let $v_1,v_2,\ldots ,v_t$ be the children of *x* in $\Gamma$ and suppose that $Q^\lambda _x$ is monotone. Then, $T^{\mathcal {PT}}$ is monotone.

### Proof

If ${T^{\mathcal {PT}}[{x, \lambda ,\phi ,\eta }]\ne \infty }$, there are $D\subseteq U\subseteq \phi (x)$ such that the minimum in Equation (7) in Definition 7 is attained, that is,

$$\begin{aligned} T^{\mathcal {PT}}[{x,\lambda ,\phi ,\eta }]&= Q_{x}^{{\lambda }{x\rightarrow U}}\left[ {0, f_{U,D}(\phi ), g_{U,D}(\eta )}\right] + c_{U,D}\\&= Q_{x}^{\lambda }[{0, f_{U,D}(\phi ), g_{U,D}(\eta )}] + c^*_{U,D} \end{aligned}$$

for some constants $c_{U,D}$ and $c^*_{U,D}$ that are independant of $\phi$ and $\eta$. Since, by assumption, $Q^\lambda _x$ is monotone for all $\lambda$ and both $f_{U,D}$ and $g_{U,D}$ are monotone by Claim 6, we conclude

$$\begin{aligned} T^{\mathcal {PT}}[{x,\lambda ,\psi ,\eta }]&\ge Q_{x}^{\lambda }[{0, f_{U,D}(\psi ), g_{U,D}(\eta )}] + c^*_{U,D}\\&\ge Q_{x}^{\lambda }[{0, f_{U,D}(\psi '), g_{U,D}(\eta ')}] + c^*_{U,D} \ge T^{\mathcal {PT}}[{x,\lambda ,\psi ',\eta '}] \end{aligned}$$

Note the last “$\ge$” since we only know that this particular value participates in the minimum that forms $T^{\mathcal {PT}}[{x,\lambda ,\psi ',\eta '}]$, while this minimum may be attained at an even smaller value.$\square$

By Claim 7, in order to prove Lemma 9, it is sufficent to show that $Q^\lambda _x$ is monotone. This proof is by induction on the height of *x* in $\Gamma$ and the value of the first argument *i* of $Q^\lambda _x$.

For the induction base, suppose that *x* is a leaf of $\Gamma$ and note that *x* has $t=0$ children. If $Q_{x}^{\lambda }[{0,\psi ,\eta }]\ne \infty$, we have $\psi =\overrightarrow{0}$ and $\eta ={\overrightarrow{0}}\left[ {x\rightarrow \rho (x)}\right]$. But then, $\psi '=\psi$ and $\eta '=\eta$, implying $Q_{x}^{\lambda }[{0,\psi ',\eta '}]=Q_{x}^{\lambda }[{0,\psi ,\eta }]$.

For the induction step, let *x* have *t* children $v_1,v_2,\ldots ,v_t$ and let $0<i\le t$. First, let $\psi ^*\trianglelefteq {\psi }\mid _{\hbox {YW}_{{v_i}}^{\Gamma }}$ and $\eta ^*\trianglelefteq {\eta }\mid _{\hbox {YW}_{{v_i}}^{\Gamma }}$ be such that the minimum in Equation (8) in Definition 7 is attained, that is, ${Q_{x}^{\lambda }[{i,\psi ,\eta }]=Q_{x}^{\lambda }[{i-1,\psi -\psi ^*,\eta -\eta ^*}] +T^{\mathcal {PT}}[{v_i, {\lambda }\mid _{\hbox {YW}_{{v_i}}^{\Gamma }},\psi ^*,\eta ^*}]}$. Further, let $\psi '^*$ and $\eta '^*$ be defined as $\psi '^*(y) :=\psi '(y)\cap \psi ^*(y)$ and $\eta '^*(y) :=\min \{\eta '(y),\eta ^*(y)\}$. Clearly, $\psi '^*\trianglelefteq \psi '$ and $\psi '^*\trianglelefteq \psi ^*$ and $\eta '^*\trianglelefteq \eta '$ and $\eta '^*\trianglelefteq \eta ^*$. Further, for all *y*,

18 $$\begin{aligned} (\psi ' - \psi '^*)(y)&= \psi '(y) \setminus (\psi '(y)\cap \psi ^*(y))=\psi '(y)\setminus \psi ^*(y)\nonumber \\&\subseteq \psi (y)\setminus \psi ^*(y) =(\psi -\psi ^*)(y) \end{aligned}$$

19 $$\begin{aligned} (\eta ' - \eta '^*)(y)&= \eta '(y) - \min \{\eta '(y), \eta ^*(y)\} = \eta '(y) {\mathop {-}\limits ^{.}}\eta ^*(y)\nonumber \\&\le \eta (y) {\mathop {-}\limits ^{.}}\eta ^*(y) = (\eta -\eta ^*)(y), \end{aligned}$$

so $\psi '-\psi '^*\trianglelefteq \psi -\psi ^*$ and $\eta '-\eta '^*\trianglelefteq \eta -\eta ^*$. Since $\psi '^*$ and $\eta '^*$ participate in the minimum in the definition of $Q_{x}^{\lambda }[{i,\psi ',\eta '}]$,

$$\begin{aligned} Q_{x}^{\lambda }[{i, \psi , \eta }]&= Q_{x}^{\lambda }[{i-1,\psi -\psi ^*,\eta -\eta ^*}]+T^{\mathcal {PT}} [{v_i,{\lambda }\mid _{\hbox {YW}_{{v_i}}^{\Gamma }},\psi ^*,\eta ^*}]\\&{\mathop {\ge }\limits ^{IH,(18),(19)}} Q_{x}^{\lambda }[{i-1,\psi '-\psi '^*,\eta '-\eta '^*}]+T^{\mathcal {PT}} [{v_i,{\lambda }\mid _{\hbox {YW}_{{v_i}}^{\Gamma }},\psi '^*,\eta '^*}]\\&\ge Q_{x}^{\lambda }[{i,\psi ',\eta '}] \end{aligned}$$

$$\square $$

### Lemma 10

*Let* $\Gamma$* be a tree agreeing with* *N*, let $x\in V(N)$,* let *$\psi _x,\lambda _x:\hbox {YW}_{x}^{\Gamma }\rightarrow 2^{c}$* and *$\eta _x:\hbox {YW}_{x}^{\Gamma }\rightarrow \mathbb {N}$.* Let* *f** minimize* $\hbox {cost}_{(f)}$* among all lineage functions in* $\mathcal {LF}_{N,\phi }$* such that, for all* $w\in \hbox {YW}_{x}^{\Gamma }$, $\lambda _x(w) = f(w)$, $\psi _x(w) = f(w)\cap \bigcup _{u\in \hbox {Pred}_{N}^{\uparrow x}{(w)}}f(u)$,* and *$\eta _x(w) \le \sum _{u\in \hbox {Pred}_{N}{\uparrow x}{(w)}}|f(u)|$. *If there are no such* *f*, then ${T^{\mathcal {PT}}[{x,\lambda _x,\psi _x,\eta _x}=\infty ]}$.* Otherwise,*

$$\begin{aligned} {T^{\mathcal {PT}}[{x,\lambda _x, \psi _x, \eta _x}] = \sum _{z\le _\Gamma x}\hbox {cost}_{f}{(z)}} \end{aligned}$$

### Proof

Note that, if the cost of *f* is finite, then $|f(v)|\le \sum _{u\in \hbox {Pred}{(v)}}|f(u)|$ for all $v\ne \rho _N$ and $|f(\rho _N)|=1$ by Definition 4. Again, the proof is by induction on the height of *x* in $\Gamma$.

**Case 1:** *x* is a leaf in $\Gamma$, that is, $t=0$ and $\hbox {Pred}_{N}^{\uparrow x}{(v)}\subseteq \{x\}$ for all *v*. Then, by Definition 7, ${T^{\mathcal {PT}}[{x,\lambda _x,\psi _x,\eta _x}]}$ is finite if and only if

$$\begin{aligned} {\psi _x}\left[ {\begin{array}{l} x\rightarrow D\\ \forall _{y\in \hbox {Succ}_{N}^{\uparrow }{(x)}}y\rightarrow \psi _x(y)\setminus U \end{array}}\right]&=\overrightarrow{0} \end{aligned}$$

and

$$\begin{aligned} {\eta _x}\left[ {\begin{array}{l} x\rightarrow |U|{\mathop {-}\limits ^{.}}\sum _{r\in \hbox {Pred}_{N}^{\downarrow }{(x)}}|\lambda _x(r)|\\ \forall _{y\in \hbox {Succ}_{N}^{\uparrow }{(x)}}y\rightarrow \eta _x(y){\mathop {-}\limits ^{.}}|U| \end{array}}\right]&={\overrightarrow{0}}\left[ {x\rightarrow \rho (x)}\right] , \end{aligned}$$

if and only if (considering the assignments of the above modifications of $\psi _x$ and $\eta _x$ individually) (a) $D=\varnothing$, (b) $|U|{\mathop {-}\limits ^{.}}\sum _{r\in \hbox {Pred}_{N}^{\downarrow }{(x)}}|\lambda _x(r)|=\rho (x)$ (c) for each $y\in \hbox {Succ}_{N}{(x)}$,

20 $$\begin{aligned} U\supseteq \psi _x(y)=f(y)\cap f(x). \,&\text {and}&|U|\ge \eta _x(y)=|f(x)| \end{aligned}$$

In this case, the table entry is assigned the cost $|U\setminus \bigcup _{r\in \hbox {Pred}_{N}^{\downarrow }{(x)}}\lambda _x(r)| - \rho (x) = |U\setminus \bigcup _{r\in \hbox {Pred} {(x)}}f(r)| - \rho (x)$. If $x=\rho _N$, this simplifies to $|U| - 1$ and, since $|f(\rho _N)|=1$, the cost is minimized by $U=f(\rho _N)$ and the table entry equals $0 = \hbox {cost}_{f}{(\rho _N)}$. Thus, in the following, let $x\ne \rho _N$.

“$\le$”: Since (20) is satisfied for $U=f(x)$, the minimum over all *U* is at most the cost when choosing $U=f(x)$, which is $|f(x)\setminus \bigcup _{r\in \hbox {Pred}{(x)}}f(r)|=\hbox {cost}_{f}{(x)}$

“$\ge$”: Towards a contradiction, assume that there is a *U* satisfying (20) such that ${T^{\mathcal {PT}}[{x,\lambda _x,\psi _x,\eta _x}]=|U\setminus \bigcup _{r\in \hbox {Pred}{(x)}}f(r)| < |f(x)\setminus \bigcup _{r\in \hbox {Pred}{(x)}}f(r)| = \hbox {cost}_{f}{(x)}}$. We show that $f':={f}\left[ {x\rightarrow U}\right]$ has less overall cost than *f*, contradicting its optimality. Since changing *f*(*x*) to *U* only influences the cost of *x* and its children in *N*, it suffices to consider them. To this end, let *y* be any child of *x* in *N*. First, by (b), $|f'(x)|=|U|\le \sum _{r\in \hbox {Pred}_{N}{(x)}}|f'(r)|$ so $\hbox {cost}_{f'}{(x)}=|U\setminus \bigcup _{r\in \hbox {Pred}_{N}{(x)}}f(r)|<\hbox {cost}_{f}{(x)}$ by assumption. Further, $|f'(y)|=|f(y)|\le \sum _{u\in \hbox {Pred}{(y)}}|f(u)|\le \sum _{u\in \hbox {Pred}{(y)}}|f'(u)|$ since $|U|\ge |f(x)|$ by (20). Finally, for each $y\in \hbox {Succ}_{N}{(x)}$,

$$\begin{aligned} \hbox {cost}_{f}{(y)}&= |f(y)\setminus \bigcup _{v\in \hbox {Pred}{(y)}}f(v)| = |f(y)\setminus (f(x)\cup \bigcup _{v\in \hbox {Pred}{(y)}-x}f(v))|\\&{\mathop {\ge }\limits ^{(20)}} |f'(y)\setminus (U\cup \bigcup _{v\in \hbox {Pred}{(y)}-x}f'(v)))| = \hbox {cost}_{f'}{(y)} \end{aligned}$$

**Case 2:** *x* has children $v_1,v_2,\ldots ,v_t$ with $t\ge 1$ in $\Gamma$. In the following, we abbreviate $Y_i:=\bigcup _{j\le i}\hbox {YW}_{{v_j}}^{\Gamma }$ and $Z_i:=\bigcup _{j\le i}\Gamma _{v_j}$. Further, we call a lineage function $f'$ *eligible* with respect to an anti-chain *Y* in $\Gamma$ and functions $\lambda '$, $\psi '$, and $\eta '$ if, for all $w\in \bigcup _{y\in Y}\hbox {YW}_{{y}}^{\Gamma }$, we have $\lambda (w) = f'(w)$, $\psi '(w) \subseteq f'(w)\cap \bigcup _{y\in Y}\bigcup _{u\in \hbox {Pred}_{N}^{\uparrow y}{(w)}}f'(u)$ and $\eta '(w) \le \sum _{y\in Y}\sum _{u\in \hbox {Pred}_{N}^{\uparrow y}{(w)}}|f(u)|+\rho (w)$ and the cost of $f'$ is finite on $\bigcup _{y\in Y}\Gamma _y$. We first show how the table $Q_{x}^{\lambda }$ is used to distribute lineages among the $v_i$.$\square$

### Claim 8

Let $1\le i \le t$, Let $\eta _i:Y_i\rightarrow \mathbb {N}$ and let $\lambda ,\psi _i:Y_i\rightarrow 2^{C}$ with $\psi _i\trianglelefteq \lambda$. Let $f_i$ minimize $\sum _{z\in Z_i}\hbox {cost}_{{f_i}}{(z)}-\rho (x)$ among all lineage functions that are eligible with respect to $Y_i$, $\lambda$, $\psi _i$, and $\eta _i$. If no such *f* exists, then $Q_{x}^{\lambda }[{i,\psi _i,\eta _i}]=\infty$. Otherwise,

$$\begin{aligned} Q_{x}^{\lambda }[{i,\psi _i,\eta _i}] = \sum _{z\in Z_i}\hbox {cost}_{{f_i}}{(z)} - \rho (x). \end{aligned}$$

### Proof

The proof of the claim is by induction on *i*.

**Case** $i=1$: By Definition 7, $Q_{x}^{\lambda }[{0,\psi _1-\psi ',\eta _1-\eta '}]$ is finite if and only if $\psi ' = \psi _1$, $\eta ' = \eta _1$ and ${T^{\mathcal {PT}}[{v_1,{\lambda }\mid _{\hbox {YW}_{{v_1}}^{\Gamma }},\psi ',\eta '}]}$ is finite, that is, by induction hypothesis of the lemma, there is a lineage function $f'$ that is eligible for $Y_1$, $\lambda$, $\psi _1=\psi '$ and $\eta _1=\eta '$. Thus, the first part of the claim follows. Since $\psi _1$ and $\eta _1$ are the only valid choices for the minima in (8) that result in finite values, we conclude

$$\begin{aligned} {Q_{x}^{\lambda }[{1,\psi _1,\eta _1}] = -\rho (x) + T^{\mathcal {PT}}[{v_1,{\lambda }\mid _{\hbox {YW}_{{v_1}}^{\Gamma }},\psi _1,\eta _1}] {\mathop {=}\limits ^{\text {IH lemma}}} \sum _{z\in Z_1}\hbox {cost}_{{f_i}}{(z)} - \rho (x)} \end{aligned}$$

since $f_i$ is eligible with respect to $Y_1$, $\lambda$, $\psi _1$ and $\eta _1$ and minimizes $\sum _{z\in Z_1}\hbox {cost}_{{f_i}}{(z)}-\rho (x)$.

**Case** $i>1$: First, suppose that $Q_{x}^{\lambda }[{i,\psi _i,\eta _i}]\ne \infty$. By (8), there are $\psi '\trianglelefteq \psi _i$ and $\eta '\trianglelefteq \eta _i$ such that $Q_{x}^{\lambda }[{i-1,\psi _{i-1},\eta _{i-1}}]\ne \infty$ and ${T^{\mathcal {PT}}[{v_i,{\lambda }\mid _{\hbox {YW}_{{v_i}}^{\Gamma }},\psi ',\eta '}]\ne \infty }$, where $\psi _{i-1}:=\psi _i-\psi '$ and $\eta _{i-1}:=\eta _i-\eta '$. By induction hypotheses, there are functions $f_{i-1}$ and $f'$ such that $f_{i-1}$ is eligible with respect to $Y_{i-1}$, $\lambda$, $\psi _{i-1}$, $\eta _{i-1}$ and $f'$ is eligible with respect to $\{v_i\}$, $\lambda$, $\psi '$, $\eta '$. We construct a function $f^*$ by setting

$$\begin{aligned} f^*(w) := \left\{ \begin{array}{ll} f'(w) &{} \text {if}\,w\in \hbox {YW}_{{v_i}}^{\Gamma }\cup \Gamma _{v_i}\\ f_{i-1}(w) &{} \text {if}\,w\in \bigcup _{y\in Y_{i-1}}\left( \hbox {YW}_{{y}}^{\Gamma }\cup \Gamma _{v_j}\right) \setminus \hbox {YW}_{{v_i}}^{\Gamma }\\ C &{} \text {otherwise}. \end{array}\right. \end{aligned}$$

(Note that the cost of *f* on *N* might be $\infty$ but we will see that its cost on $Z_i$ is finite). First, we show that $f^*$ is eligible with respect to $Y_i$, $\lambda$, $\psi _i$, and $\eta _i$. To this end, let $w\in \hbox {YW}_{{y}}^{\Gamma }$ for any $y\in Y_i$. Then, by eligibility of $f'$ and $f_{i-1}$ and $\Gamma _{v_j}\cap \Gamma _{v_i}=\varnothing$ for all $j<i$, we have

$$\begin{aligned} f^*(w)&= \left\{ \begin{array}{ll} f'(w)={\lambda }\mid _{\hbox {YW}_{{v_i}}^{\Gamma }}(w)=\lambda (w) &{} \text {if}\,w\in \hbox {YW}_{{v_i}}^{\Gamma }\\ f^*(w)=f_{i-1}(w)={\lambda }\mid _{\bigcup _{j<i}\hbox {YW}_{{v_j}}^{\Gamma }}(w)=\lambda (w) &{} \text {otherwise} \end{array}\right. \\ \psi _i(w)&= \psi _{i-1}(w)\cup \psi '(w)\\&\subseteq \left( f_{i-1}(w)\cap \bigcup _{y\in Y_{i-1}}\bigcup _{u\in \hbox {Pred}_{N}^{\uparrow y}{(w)}}f_{i-1}(u) \right) \cup \left( f'(w)\cap \bigcup _{u\in \hbox {Pred}_{N}^{\uparrow v_i}{(w)}}f'(u) \right) \\&= f^*(w)\cap \bigcup _{y\in Y_i}\bigcup _{u\in \hbox {Pred}_{N}^{\uparrow y}{(w)}}f^*(w).\\ \eta _i(w)&= \eta _{i-1}(w) + \eta '(w) \le \sum _{y\in Y_{i-1}}\sum \limits _{u\in \hbox {Pred}_{N}^{\uparrow y}{(w)}}|f_{i-1}(u)| + \sum \limits _{u\in \hbox {Pred}_{N}^{\uparrow v_i}{(w)}}|f'(u)|\\&= \sum _{y\in Y_i}\sum \limits _{u\in \hbox {Pred}_{N}^{\uparrow y}{(w)}}|f^*(u)|. \end{aligned}$$

Finally, the cost of $f^*$ on $Z_i$ equals the cost of $f_{i-1}$ on $Z_{i-1}$ plus the cost of $f'$ on $\Gamma _{v_i}$ and is, therefore, finite. Thus, $f^*$ is eligible for $Y_i$, $\lambda$, $\psi _i$ and $\eta _i$, implying the contraposition of the first part of the lemma. For the cost equality, we consider both directions separately.

“$\le$”: Let $\psi ':\hbox {YW}_{{v_i}}^{\Gamma }\rightarrow 2^{C}$ and $\eta ':\hbox {YW}_{{v_i}}^{\Gamma }\rightarrow \mathbb {N}$ be defined on $\hbox {YW}_{{v_i}}^{\Gamma }$ as

$$\begin{aligned} \psi '(w) :=&\psi _i(w)\cap f_i(w)\cap \bigcup _{u\in \hbox {Pred}_{N}^{\uparrow v_i}{(w)}}f_i(u) \end{aligned}$$

and

$$\begin{aligned} \eta '(w) :=&\min \{\eta _i(w),\sum \limits _{u\in \hbox {Pred}_{N}^{\uparrow v_i}{(w)}}|f_i(u)|+\rho (w)\}. \end{aligned}$$

Clearly, $\psi '\trianglelefteq {\psi _i}\mid _{\hbox {YW}_{{v_i}}^{\Gamma }}$ and $\eta '\trianglelefteq {\eta _i}\mid _{\hbox {YW}_{{v_i}}^{\Gamma }}$. Furthermore, define $\psi _{i-1}$ and $\eta _{i-1}$ by, for all $w\in \bigcup _{y\in Y_{i-1}}\hbox {YW}_{{y}}^{\Gamma }$, setting

$$\begin{aligned} \psi _{i-1}(w) := \psi _i(w) - \psi '(w)&\subseteq \left( f'(w)\cap \bigcup _{y\in Y_i}\bigcup _{u\in \hbox {Pred}_{N}^{\uparrow y}{(w)}}f'(u) \right) \setminus \left( f'(w)\cap \bigcup _{u\in \hbox {Pred}_{N}^{\uparrow v_i}{(w)}}f'(u) \right) \\&\subseteq f'(w)\cap \bigcup _{y\in Y_{i-1}}\bigcup _{u\in \hbox {Pred}_{N}^{\uparrow y}{(w)}}f'(u) \end{aligned}$$

and

$$\begin{aligned} \eta _{i-1}(w) := \eta _i(w) - \eta '(w)&\le \sum _{y\in Y_i}\sum \limits _{u\in \hbox {Pred}_{N}^{\uparrow y}{(w)}}|f_i(u)| - \sum \limits _{u\in \hbox {Pred}_{N}^{\uparrow v_i}{(w)}}|f_i(u)|\\&= \sum _{y\in Y_{i-1}}\sum \limits _{u\in \hbox {Pred}_{N}^{\uparrow y}{(w)}}|f_i(u)|. \end{aligned}$$

Thus, $f_i$ is eligible with respect to $Y_{i-1}$, $\lambda$, $\psi _{i-1}$ and $\eta _{i-1}$, implying

$$\begin{aligned} Q_{x}^{\lambda }[{i,\psi _i,\eta _i}]&{\mathop {\le }\limits ^{\text {Def.~7}}} Q_{x}^{\lambda }[{i-1,\psi _i-\psi ',\eta _i-\eta '}] + T^{\mathcal {PT}}[{v_i,{\lambda }\mid _{\hbox {YW}_{{v_i}}^{\Gamma }},\psi ',\eta '}]\\&{\mathop {\le }\limits ^{\text {Lem.~9}}} Q_{x}^{\lambda }[{i-1,\psi _{i-1},\eta _{i-1}}] + T^{\mathcal {PT}} [{v_i,{\lambda }\mid _{\hbox {YW}_{{v_i}}^{\Gamma }},\psi ',\eta '}]\\&{\mathop {\le }\limits ^{\begin{array}{c} \text {IH claim}\\ \text {IH lemma} \end{array}}} \sum _{z\in Z_{i-1}}\hbox {cost}_{{f_i}}{(z)} - \rho (x) + \sum _{z\le v_i}\hbox {cost}_{{f_i}}{(z)}\\&= \sum _{z\in Z_i}\hbox {cost}_{{f_i}}{(z)} - \rho (x) \end{aligned}$$

“$\ge$”: Let $Q_{x}^{\lambda }[{i,\psi _i,\eta _i}]$ be finite as, otherwise, “$\ge$” trivially holds. By (8), there are $\psi '\trianglelefteq {\psi _i}\mid _{\hbox {YW}_{{v_i}}^{\Gamma }}$ and $\eta '\trianglelefteq {\eta _i}\mid _{\hbox {YW}_{{v_i}}^{\Gamma }}$ such that

$$\begin{aligned} Q_{x}^{\lambda }[{i,\psi _i,\eta _i}] = Q_{x}^{\lambda }[{i-1,\psi _i-\psi ',\eta _i-\eta '}] + T^{\mathcal {PT}}[{v_i,{\lambda }\mid _{\hbox {YW}_{{v_i}}^{\Gamma }},\psi ',\eta '}] \end{aligned}$$

Towards a contradiction, assume that this value is strictly smaller than $\sum _{z\in Z_i}\hbox {cost}_{{f_i}}{(z)}-\rho (x)$. By the induction hypothesis of the lemma, there is a lineage function $f'$ that is eligible with respect to $\{v_i\}$, $\lambda$, $\psi '$, and $\eta '$ with ${T^{\mathcal {PT}}[{v_i,{\lambda }\mid _{\hbox {YW}_{{v_i}}^{\Gamma }},\psi ',\eta '}] =\sum _{z\le _\Gamma v_i}\hbox {cost}_{f'}{(z)}}$. Further, by the induction hypothesis of the claim, there is a lineage function $f_{i-1}$ that is eligible with respect to $Y_{i-1}$, $\lambda$, $\psi _i-\psi '$, and $\eta _i-\eta '$ with $Q_{x}^{\lambda }[{i-1,\psi _i-\psi ',\eta _i-\eta '}]=\sum _{z\in Z_{i-1}}\hbox {cost}_{{f_{i-1}}}{(z)}-\rho (x)$. We construct a lineage function $f^*$ by setting

$$\begin{aligned} f^*(w) := \left\{ \begin{array}{ll} f_{i-1}(w) &{} \text {if}\,w\in Z_{i-1}\\ f'(w) &{} \text {if}\, w\in \Gamma _{v_i}\\ f_i(w) &{} \text {otherwise} \end{array}\right. \end{aligned}$$

By eligibility of $f_{i-1}$, $f_i$ and $f'$, we know that $f_{i-1}$, $f_i$ and $f^*$ coincide with $\lambda$ on $\bigcup _{y\in Y_{i-1}}\hbox {YW}_{{y}}^{\Gamma }$ and $f'$, $f_i$ and $f^*$ coincide with $\lambda$ on $\hbox {YW}_{{v_i}}^{\Gamma }$. To contradict optimality of *f*, it thus suffices to show that $f^*$ is eligible with respect to $Y_i$, $\lambda$, $\psi _i$, and $\eta _i$, To this end, note that, for all $w\in \bigcup _{y\in Y_i}\hbox {YW}_{{y}}^{\Gamma }$, we have

$$\begin{aligned} \psi _i(w)&= (\psi _i-\psi ')(w) \cup \psi '(w)\\&\subseteq \left( \lambda (w)\cap \bigcup _{y\in Y_{i-1}}\bigcup _{u\in \hbox {Pred}_{N}^{\uparrow y}{(w)}}f_{i-1}(u)\right) \cup \left( \lambda (w)\cap \bigcup _{u\in \hbox {Pred}_{N}^{\uparrow v_i}{(w)}}f'(u)\right) \\&= f^*(w)\cap \bigcup _{y\in Y_i}\bigcup _{u\in \hbox {Pred}_{N}^{\uparrow y}{(w)}}f^*(u) \end{aligned}$$

as well as

$$\begin{aligned} \eta _i(w)&= (\eta _i - \eta ')(w) + \eta '(w) \le \sum _{y\in Y_{i-1}}\sum \limits _{u\in \hbox {Pred}_{N}^{\uparrow y}{(w)}}|f_{i-1}(u)| + \sum \limits _{u\in \hbox {Pred}_{N}^{\uparrow v_i}{(w)}}|f'(u)| + \rho (w)\\&= \sum _{y\in Y_i}\sum \limits _{u\in \hbox {Pred}_{N}^{\uparrow y}{(w)}}|f^*(u)| + \rho (w) \end{aligned}$$

$$\square $$

Having established the equality for $Q_{x}^{\lambda }$, we can now prove the lemma for $i>1$. For the first part, suppose that ${T^{\mathcal {PT}}[{x,\lambda _x,\psi _x,\eta _x}]\ne \infty }$. By (7), there are $D\subseteq U\subseteq \phi (x)$ such that ${T^{\mathcal {PT}}[{x,\lambda _x,\psi _x,\eta _x}] = Q_{x}^{{\lambda _x}{x\rightarrow U}}\left[ {t,\psi _t,\eta _t}\right] + |U\setminus (D\cup \bigcup _{u\in \hbox {Pred}_{N}^{\downarrow }{(x)}}\lambda _x(u))|}$, where

$$\begin{aligned} \psi _t&:={\psi _x}\left[ {x\rightarrow D, \forall _{w\in \hbox {Succ}_{N}^{\uparrow }{(x)}}w\rightarrow \psi _x(w)\setminus U}\right] \text { and}\\ \eta _t&:={\eta _x}\left[ {x\rightarrow |U| {\mathop {-}\limits ^{.}}\sum \limits _{u\in \hbox {Pred}_{N}^{\downarrow }{(x)}}|\lambda _x(u)|, \forall _{w\in \hbox {Succ}_{N}^{\uparrow }{(x)}}w\rightarrow \eta _x(w){\mathop {-}\limits ^{.}}|U|}\right] . \end{aligned}$$

By Claim 8, there is a lineage function $f_t$ that is eligible for $\{v_t\}$, $\lambda _t:={\lambda _x}\left[ {x\rightarrow U}\right]$, $\psi _t$, and $\eta _t$. Without loss of generality, suppose that $f_t(w)=\lambda _t(w)$ for all $w\in (\hbox {YW}_{x}^{\Gamma }\cup \{x\})\setminus \bigcup _{y\in Y_t}\hbox {YW}_{{y}}^{\Gamma }$. In particular, $f_t(x)=\lambda _t(x)=U$ and $f_t$ has finite cost on $Z_t$. We show that $f_t$ is eligible with respect to $\{x\}$, $\lambda _x$, $\psi _x$ and $\eta _x$. First, assume that $\hbox {cost}_{{f_t}}{(x)}=\infty$, that is, either $x=\rho _N$ and $|f_t(x)|=|U|>1$ or $x\ne \rho _N$ and $|f_t(x)|>\sum _{u\in \hbox {Pred}_{N}{(x)}}|f_t(u)|$. In the first case, $n_t(x)=|U| > 1$, contradicting $\eta _t(x)\le \rho (x)$. In the second case, $n_t(x)=|U|{\mathop {-}\limits ^{.}}\sum _{u\in \hbox {Pred}_{N}^{\downarrow }{(x)}}|\lambda _x(u)|$, implying

$$\begin{aligned} |f_t(x)| > \sum \limits _{u\in \hbox {Pred}_{N}{(x)}}|f_t(u)| = \sum \limits _{u\in \hbox {Pred}_{N}^{\downarrow }{(x)}}|f_t(u)| + \sum \limits _{y\in Y_t}\sum \limits _{u\in \hbox {Pred}_{N}^{\uparrow y}{(x)}}|f_t(u)| \ge \sum \limits _{u\in \hbox {Pred}_{N}^{\downarrow }{(x)}}|\lambda _x(u)| + n_t(x) \ge |U| \end{aligned}$$

contradicting $f_t(x)=U$. Further, for each $w\in \hbox {YW}_{x}^{\Gamma }\setminus \hbox {Succ}_{N}^{\uparrow }{(x)}$,

$$\begin{aligned} \psi _x(w)&= \psi _t(w) \subseteq f_t(w)\cap \bigcup _{y\in Y_t}\bigcup _{u\in \hbox {Pred}_{N}^{\uparrow y}{(w)}}f_t(u) = f_t(w)\cap \bigcup _{u\in \hbox {Pred}_{N}^{\uparrow x}{(w)}}f_t(u)\\ \eta _x(w)&= \eta _t(w) \le \sum _{y\in Y_y}\sum \limits _{u\in \hbox {Pred}_{N}^{\uparrow y}{(w)}}|f_t(u)| = \sum \limits _{u\in \hbox {Pred}_{N}^{\uparrow x}{(w)}}|f_t(u)| \end{aligned}$$

and, for each $w\in \hbox {Succ}_{N}^{\uparrow }{(x)}$,

$$\begin{aligned} \psi _x(w)&\subseteq \psi _t(w) \cup U \subseteq f_t(w)\cap \bigcup _{y\in Y_t}\bigcup _{u\in \hbox {Pred}_{N}^{\uparrow y}{(w)}}f_t(u) \cup f_t(x) = f_t(w)\cap \bigcup _{u\in \hbox {Pred}_{N}^{\uparrow x}{(w)}}f_t(u)\\ \eta _x(w)&\le \eta _t(w) + |U| \le \sum _{y\in Y_y}\sum \limits _{u\in \hbox {Pred}_{N}^{\uparrow y}{(w)}}|f_t(u)| + |f_t(x)| = \sum \limits _{u\in \hbox {Pred}_{N}^{\uparrow x}{(w)}}|f_t(u)| \end{aligned}$$

Thus, $f_t$ is eligible with respect to $\{x\}$, $\lambda _x$, $\psi _x$ and $\eta _x$, implying the first part of the lemma. For the second part, we consider the directions seperately.

“$\ge$”: We pick up the definition of $f_t$ and show that ${T^{\mathcal {PT}}[{x,\lambda _x,\psi _x,\eta _x}]\ge \sum _{z\in \Gamma _x} \hbox {cost}_{{f_t}}{(z)}}$. Then, “$\ge$” follows from optimality of *f* on $\Gamma _x$. Indeed,

$$\begin{aligned} T^{\mathcal {PT}}[{x,\lambda _x,\psi _x,\eta _x}]&= Q_{x}^{{\lambda _x}{x\rightarrow U}}\left[ {t,\psi _t,\eta _t}\right] + |U\setminus (D\cup \bigcup _{u\in \hbox {Pred}_{N}^{\downarrow }{(x)}}\lambda _x(u))| \end{aligned}$$

and, since $$\psi _t(x) = D\subseteq f_t(x)\cap \bigcup _{u\in \hbox {Pred}_{N}^{\uparrow x}{(x)}}f_t(u)$$,

$$\begin{aligned}&\ge \sum _{z\in Z_t}\hbox {cost}_{{f_t}}{(z)} + |f_t(x)\setminus (\bigcup _{u\in \hbox {Pred}_{N}^{\uparrow }{(x)}}f_t(u)\cup \bigcup _{u\in \hbox {Pred}_{N}^{\downarrow }{(x)}}f_t(u))|\\&= \sum _{z\in Z_t}\hbox {cost}_{{f_t}}{(z)} + \hbox {cost}_{{f_t}}{(x)} = \sum _{z\in \Gamma _x}\hbox {cost}_{{f_t}}{(z)} \end{aligned}$$

“$\le$”: Let $U:=f(x)$ and let $D:=f(x)\cap \bigcup _{u\in \hbox {Pred}_{N}^{\uparrow x}{(x)}}f(u)\subseteq U$. Then, $|U\setminus (D\cup \bigcup _{u\in \hbox {Pred}_{N}^{\downarrow }{(x)}}f(u)\lambda _x(u))|=\hbox {cost}_{f}{(x)} + \rho (x)$. Further, let

$$\begin{aligned} \psi _t&:={\psi _x}\left[ {x\rightarrow D, \forall _{w\in \hbox {Succ}_{N}^{\uparrow }{(x)}}w\rightarrow \psi _x(w)\setminus U}\right] \text { and}\\ \eta _t&:={\eta _x}\left[ {x\rightarrow |U| {\mathop {-}\limits ^{.}}\sum \limits _{u\in \hbox {Pred}_{N}^{\downarrow }{(x)}}|\lambda _x(u)|, \forall _{w\in \hbox {Succ}_{N}^{\uparrow }{(x)}}w\rightarrow \eta _x(w){\mathop {-}\limits ^{.}}|U|}\right] . \end{aligned}$$

We show that *f* is eligible with respect to $Y_t$, $\lambda _x$, $\psi _t$ and $\eta _t$. Then, $Q_{x}^{{\lambda _x}{x\rightarrow D}}[{t,\psi _t,\eta _t}]=\sum _{z\in Z_t}\hbox {cost}_{f}{(z)} - \rho (x)$ by Claim 8, implying ${T^{\mathcal {PT}}[{x,\lambda _x,\psi _x,\eta _x}]\le \sum _{z\in Z_t}\hbox {cost}_{f}{(z)}-\rho (x) + \hbox {cost}_{f}{(x)} + \rho (x)=\sum _{z\in \Gamma _x}\hbox {cost}_{f}{(z)}}$ since *U* and *D* are valid choices for the minimum in (7).

To see that *f* is eligible, note that $f(w) = {\lambda _x}\left[ {x\rightarrow U}\right]$ for all $w\in \bigcup _{y\in Y_t}\hbox {YW}_{{y}}^{\Gamma }$ since $\bigcup _{y\in Y_t}\hbox {YW}_{{y}}^{\Gamma }\subseteq \hbox {YW}_{x}^{\Gamma }\cup \{x\}$. Further, for the conditions on $\psi _t$ and $\eta _t$, consider three cases for nodes in $\bigcup _{y\in Y_t}\hbox {YW}_{{y}}^{\Gamma }$. First, if $w=x$, then

$$\begin{aligned} \psi _t(x)&= D = f(x)\cap \bigcup _{u\in \hbox {Pred}_{N}^{\uparrow x}{(x)}}f(u) = f(x)\cap \bigcup _{y\in Y_t}\bigcup _{u\in \hbox {Pred}_{N}^{\uparrow y}{(x)}}f(u)\\ \eta _t(x)&= |U|{\mathop {-}\limits ^{.}}\sum \limits _{u\in \hbox {Pred}_{N}^{\downarrow }{(x)}}|\lambda _x(u)| = |f(x)|{\mathop {-}\limits ^{.}}\sum \limits _{u\in \hbox {Pred}_{N}^{\downarrow }{(x)}}|f(u)| {\mathop {\le }\limits ^{\text {Def.~4}}} \sum \limits _{u\in \hbox {Pred}_{N}^{\uparrow }{(x)}}|f(u)|\\&= \sum _{y\in Y_t}\sum \limits _{u\in \hbox {Pred}_{N}^{\uparrow }{(x)}}|f(u)| \end{aligned}$$

Second, if $w\in \bigcup _{y\in Y_t}\hbox {YW}_{{y}}^{\Gamma }\cap \hbox {Succ}_{N}^{\uparrow }{(x)}$, then

$$\begin{aligned} \psi _t(w)&= \psi _x(w)\setminus U \subseteq f(w)\cap \bigcup _{u\in \hbox {Pred}_{N}^{\uparrow x}{(w)}}f(u)\setminus f(x) = f(w)\cap \bigcup _{u\in \hbox {Pred}_{N}^{\uparrow x}{(w)}\setminus \{x\}}f(u)\\&= f(w)\cap \bigcup _{y\in Y_t}\bigcup _{u\in \hbox {Pred}_{N}^{\uparrow y}{(w)}}f(u) \end{aligned}$$

as well as

$$\begin{aligned} \eta _t(w)&= \eta _x(w){\mathop {-}\limits ^{.}}|U| \le \sum \limits _{u\in \hbox {Pred}_{N}^{\uparrow x}{(w)}}|f(u)| + \rho (x) {\mathop {-}\limits ^{.}}|f(x)|\\&= \sum \limits _{u\in \hbox {Pred}_{N}^{\uparrow x}{(w)}\setminus \{x\}}|f(u)| + |f(x)| + \rho (x) {\mathop {-}\limits ^{.}}|f(x)| = \sum \limits _{y\in Y_t}\sum \limits _{u\in \hbox {Pred}_{N}^{\uparrow y}{(w)}}|f(u)| + \rho (x) \end{aligned}$$

Otherwise, $w\in \bigcup _{y\in Y_t}\hbox {YW}_{{y}}^{\Gamma }\setminus (\hbox {Succ}_{N}^{\uparrow }{(x)}\cup \{x\})$ and we have

$$\begin{aligned} \psi _t(w)&= \psi _x(w) \subseteq f(w)\cap \bigcup _{u\in \hbox {Pred}_{N}^{\uparrow x}{(w)}}f(u) = f(w)\cap \bigcup _{y\in Y_t}\bigcup _{u\in \hbox {Pred}_{N}^{\uparrow x}{(y)}}f(u)\\ \eta _t(w)&= \eta _x(w) \le \sum \limits _{u\in \hbox {Pred}_{N}^{\uparrow x}{(w)}}|f(u)| + \rho (x) = \sum _{y\in Y_t}\sum \limits _{u\in \hbox {Pred}_{N}^{\uparrow y}{(w)}}|f(u)| + \rho (x) \end{aligned}$$

$$\square $$

## Footnotes

1 For example, networks whose “worst” biconnected component is equal to the result of glueing two copies of the same *n*-leaf tree at corresponding leaves are known to have treewidth two, but level at least $n-1$.

2 Intuitively, $\Gamma$ can be obtained from *T* by contracting all nodes in $V(T)\setminus F$ onto their respective parents and identifying all nodes $x\in F$ with the vertex $v\in V(G)\setminus B(x)$ of *G* that is forgotten in *x*.
